# Structure and Biosynthesis of Mangotoxin Produced by the Plant Pathogen *Pseudomonas syringae*

**DOI:** 10.1002/anie.2211678

**Published:** 2026-09-22

**Authors:** Alexis H. Murray, Sohan Hazra, Ryan W. Mull, Erinn M. O'Neill, Bo Li

**Affiliations:** ^1^ Department of Chemistry University of North Carolina at Chapel Hill Chapel Hill North Carolina USA

**Keywords:** alkyne biosynthesis, antibiotics, heme oxygenase‐like enzymes, phytotoxin

## Abstract

Pathogenic bacteria interfere with plant metabolism through specialized metabolites and mechanisms. Mangotoxin was identified in 2003 from *Pseudomonas syringae* pv. *syringae*, which causes devastating diseases globally in various crops, including mango. Mangotoxin was found to inhibit arginine biosynthesis in tomato and *Escherichia coli*; however, its chemical structure remained unknown. We determined the structure of mangotoxin from the native bacterial producer *P. syringae* to be an alanine‐citrulline dipeptide that contains an internal alkyne on citrulline. We showed that mangotoxin also inhibits the growth of a multidrug‐resistant human pathogen *Klebsiella pneumoniae* strain. In vitro characterization of mangotoxin biosynthesis revealed that alkyne formation is catalyzed by a heme oxygenase‐like domain‐containing oxidase with a redox partner on a tripeptide intermediate. We discovered that the mangotoxin biosynthetic enzymes can couple various canonical and noncanonical amino acids into tripeptides and install two‐ or four‐electron oxidations on several tripeptides, providing a foundation for bioengineering of the pathway. Our work answered a two‐decade‐old question on the structure of an elusive toxin and will facilitate the elucidation of the molecular mechanism used by mangotoxin to inhibit amino acid metabolism in plants and bacteria.

## Graphical abstract

The structure of mangotoxin remained elusive for over two decades. Here, mangotoxin was isolated from the native producer, *Pseudomonas syringae* pv. *syringae*, and was determined to be an alkyne‐containing dipeptide. Mangotoxin inhibited the growth of multidrug‐resistant human pathogen *Klebsiella pneumoniae*. Mangotoxin biosynthesis was reconstituted in vitro, and the biosynthetic enzymes were used to synthesize multiple non‐native peptides that contain an alkene or alkyne.

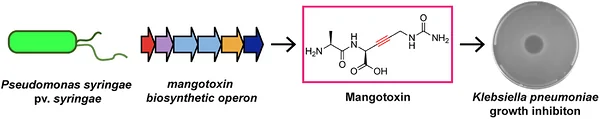

## 1 Introduction

The plant pathogen *Pseudomonas syringae* comprises over 60 pathovars, infects many crops, and causes devastating crop disease outbreaks worldwide [1]. Phytotoxins produced by *P. syringae* augment plant disease development by directly damaging plant cells or interfering with plant metabolism [2]. These phytotoxins exhibit unusual chemical structures and distinct mechanisms of action. Early examples discovered in the 1970s include the sulfodiaminophosphinyl‐containing tripeptide phaseolotoxin, which drives halo blight of beans by inhibiting ornithine carbamoyltransferase [3, 4], and the β‐lactam‐containing dipeptide tabtoxin, which promotes the highly infectious and destructive wildfire disease in tobacco by targeting glutamine synthetase [5, 6]. Structural elucidation of these toxins was crucial for understanding bacteria–plant interactions and developing disease‐resistant plants [7, 8].

*Pseudomonas syringae* pv. *syringae* (*Pss*) causes bacterial apical necrosis in mango around the globe, and also infects other crops, including beans, lemons, and tomatoes [9, 10, 11]. To prevent *Pss* infection and bacterial apical necrosis, copper‐based pesticides are commonly used; however, many *Pss* strains are copper‐resistant [12, 13]. *Pss* isolated from mango produces mangotoxin. Mangotoxin is proposed to inhibit the plant ornithine *N*‐acetyltransferase, which catalyzes the transfer of the acetyl group from *N*‐acetylornithine to glutamate, forming ornithine during arginine biosynthesis [14]. Mangotoxin also inhibited the growth of *Escherichia coli* and the growth was rescued by ornithine [14, 15]. The initial study in 2003 tracking mangotoxin activity against *E. coli* suggested that mangotoxin is a hydrophilic peptide less than 3 kilodaltons in size because it was sensitive to protease treatment and passed through filters with a 3‐kilodalton molecular weight cut‐off [14]; however, the chemical structure of mangotoxin remained unknown. De novo arginine biosynthesis in bacteria is distinct from humans and is explored as an antimicrobial target [16, 17, 18]. Knowledge of the structure and biosynthesis of mangotoxin is critical for understanding the interaction between *Pss* and plants and for discovering ways to control bacterial apical necrosis. This knowledge will also aid in the development of antimicrobial compounds that target arginine biosynthesis.

Genetic studies via transposon mutagenesis identified two operons required for mangotoxin production: the *mangotoxin generating operon* (*mgo*) and the *mangotoxin biosynthetic operon* (*mbo*) [19, 20]. We and others showed that *mgo*‐encoded proteins produce the signaling molecule leudiazen, which regulates mangotoxin production in *Pss* by inducing the expression of *mbo* (Figure 1A) [20, 21, 22, 23]. Deletion of the entire *mgo* in *Pss* (Δ*mgo*) significantly reduced virulence on tomato leaflets and decreased *mbo* expression [23]. Thus, we used Δ*mgo* as the mangotoxin‐deficient mutant in this study. The *mbo* was determined to be responsible for mangotoxin biosynthesis by heterologous expression [20]. The *mbo* is conserved in the genomes of 42 phylogenetically related strains out of 287 *P. syringae* strains, suggesting a conserved role of mangotoxin in virulence among these organisms (Figure 1B).

**FIGURE 1:**
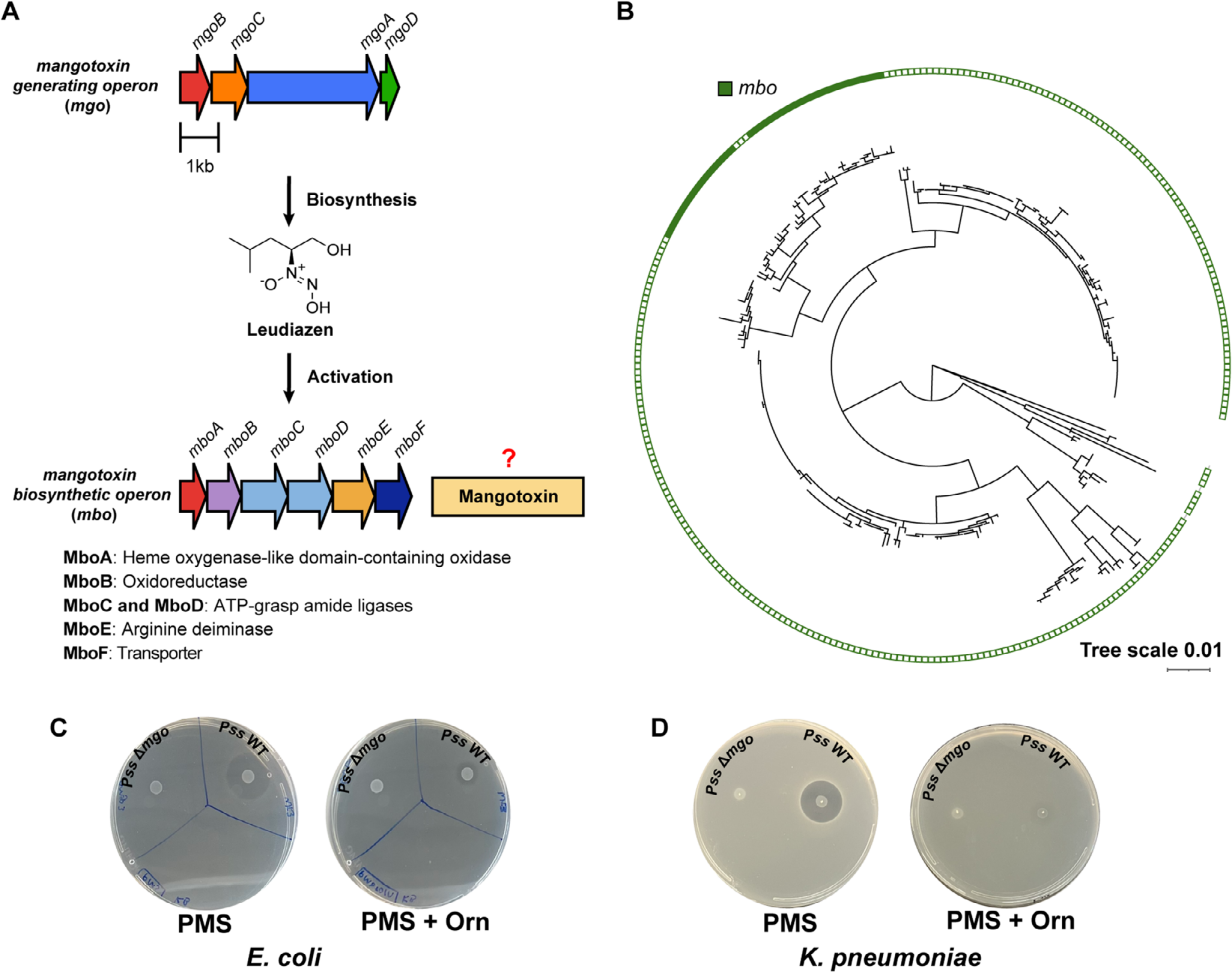
Mangotoxin production is regulated by the signaling molecule leudiazen and leads to growth inhibition of *E. coli* and *K. pneumoniae*. (A) Enzymes encoded in the *mangotoxin generating operon* (*mgo*) produce leudiazen. Leudiazen activates expression of the *mangotoxin biosynthetic operon* (*mbo*) that is responsible for producing mangotoxin. (B) Phylogenetic tree showing the distribution of *mbo* in 287 *P. syringae* strains. Solid green squares indicate organisms containing homologs of the *mbo* genes. The scale represents the number of amino acid substitutions per site. Reproduced, by permission, from Q. Guo et al., 2024, A diazeniumdiolate signal in *Pseudomonas syringae* upregulates virulence factors and promotes survival in plants, *Molecular Plant–Microbe Interactions*, 37:11, 776–783. (C) *E. coli* BL21(DE3) and (D) *K. pneumoniae* ATCC BAA‐2146 were grown as indicator strains on *Pseudomonas* minimal salts (PMS) agar without (left plates) or with ornithine (Orn, right plates). Cultures of *Pss* Δ*mgo* (5 µL, left of each plate) and *Pss* WT (5 µL, right of each plate) were spotted on top of the agar.

The *mbo* is composed of six genes (*mboA‐F*), including those encoding a putative heme oxygenase‐like domain‐containing oxidase (*mboA*), oxidoreductase (*mboB*), two ATP‐grasp amide ligases (*mboC* and *mboD*), an arginine deiminase (*mboE*), and a transporter (*mboF*) (Figure 1A). Transposon mutants of *mboA*, *mboB*, *mboC*, and *mboD* no longer displayed inhibitory activity against *E. coli* that was reversed by ornithine, and mutants of *mboE* and *mboF* exhibited lower inhibitory activity [20]. We and others showed that ATP‐grasp enzymes catalyze amide bond formation in the biosynthesis of various peptide natural products, including phaseolotoxin, tabtoxin, and phevamine A that we identified from *P. syringae* pv. *tomato* [24, 25, 26]. We proposed that the ATP‐grasp enzymes MboC and MboD catalyzed peptide bond formation in the biosynthesis of mangotoxin, which is consistent with the reported sensitivity of mangotoxin to proteases [14]. Indeed, a recent, independent report on the in vitro reconstitution of homologous Mbo enzymes from *P. syringae* PDD‐32b‐74 demonstrated that MboC and MboD catalyze the formation of a tripeptide species containing arginine and that MboA and MboB catalyze alkyne formation on an unactivated C─C bond in arginine [27]. While this work demonstrates that Mbo enzymes catalyze the formation of an alkyne‐containing tripeptide in vitro, the biological activity of the product was not reported, and the structure and biosynthesis of mangotoxin in the native producer remained unknown.

Here, we compared the metabolomes of *Pss* wildtype (WT) with the mutant deficient in inducing mangotoxin production (Δ*mgo*) to identify compounds corresponding to mangotoxin activity. This approach enabled us to isolate mangotoxin from the native producer *Pss* and to determine that mangotoxin is an alanine–citrulline dipeptide containing an internal alkyne moiety. The isolated dipeptide inhibits the growth of *E. coli*, consistent with the reported bioactivity of mangotoxin. Additionally, we demonstrate that mangotoxin inhibits the growth of multidrug‐resistant human pathogen *Klebsiella pneumoniae*. In vitro characterization of the substrate specificity of the ATP‐grasp enzymes MboC and MboD revealed the roles of these enzymes in producing a tripeptide biosynthetic intermediate, and we propose a complete biosynthetic pathway in the native producer. Further, we demonstrate that the MboA and MboB enzyme pair can catalyze the oxidation of unactivated C─C bonds to alkenes and alkynes in non‐native substrates, shedding light on the substrate scope and catalytic mechanism of the enzyme pair.

## 2 Results and Discussion

### 2.1 Mangotoxin Inhibits the Growth of Multidrug‐Resistant *Klebsiella pneumoniae*

We demonstrated that *E. coli* growth is inhibited by *Pss* WT, but not the mangotoxin‐deficient Δ*mgo* strain, and inhibition is mostly reversed by supplementing the media with ornithine (Figures 1C and S1). This activity confirmed that *Pss* produces mangotoxin as reported [14]. Using *Pss* WT and Δ*mgo* strains, we tested the antimicrobial activity of mangotoxin against another member of the *Enterobacteriaceae* family, *K. pneumoniae*. Carbapenem‐resistant *E. coli* and *K. pneumoniae* are among the most critical antimicrobial‐resistant global health threats [28, 29]. We found that the mangotoxin producer *Pss* strongly inhibited the growth of *K. pneumoniae* ATCC BAA‐2146 that is resistant to multiple antibiotics including carbapenem [29], and the growth inhibition was mostly rescued by ornithine (Figure 1D). Ornithine reversal of inhibition indicates that mangotoxin inhibits *K. pneumoniae* via a similar antimicrobial mechanism as *E. coli*.

### 2.2 Comparative Metabolomics Revealed the Potential Chemical Formula of Mangotoxin

To identify mangotoxin, we compared the metabolomes of *Pss* WT and Δ*mgo*. Cultures of both strains were tested for *E. coli* inhibition to confirm production of mangotoxin in the WT but not the Δ*mgo* mutant. Cell‐free culture supernatants of these strains were then extracted and analyzed by liquid chromatography coupled with high‐resolution mass spectrometry (LC‐HRMS). Data analysis via XCMS [30] did not yield obvious metabolites present in WT and absent in Δ*mgo*. Based on our hypothesis that mangotoxin is a hydrophilic peptide, we derivatized the extract using fluorenylmethyloxycarbonyl chloride (Fmoc‐Cl) and further analyzed the samples using LC‐HRMS. Comparative analysis revealed several ions unique to the WT, which corresponded to two compounds that eluted at different retention times (Figures 2A, B, S2, and Table S1). Targeted tandem MS (MS/MS) analyses were performed on the main ions, and the molecular formulae of the compounds were assigned using Sum formula Identification by Ranking Isotope patterns Using Mass Spectrometry (SIRIUS) [31] as C_9_H_14_N_4_O_4_ (**1**, *m*/*z* 465.1769, [Fmoc‐M+H]^+^) and C_9_H_15_N_5_O_3_ (**2**, *m/z* 464.1928, [Fmoc‐M+H]^+^). The formulae of **1** or **2** do not match any combinations of canonical amino acids. Instead, MS/MS analyses suggest that **1** and **2** are alanine–citrulline (Ala‐Cit) and alanine–arginine (Ala‐Arg) dipeptides, respectively, which had lost four hydrogen atoms (Figure S3).

**FIGURE 2:**
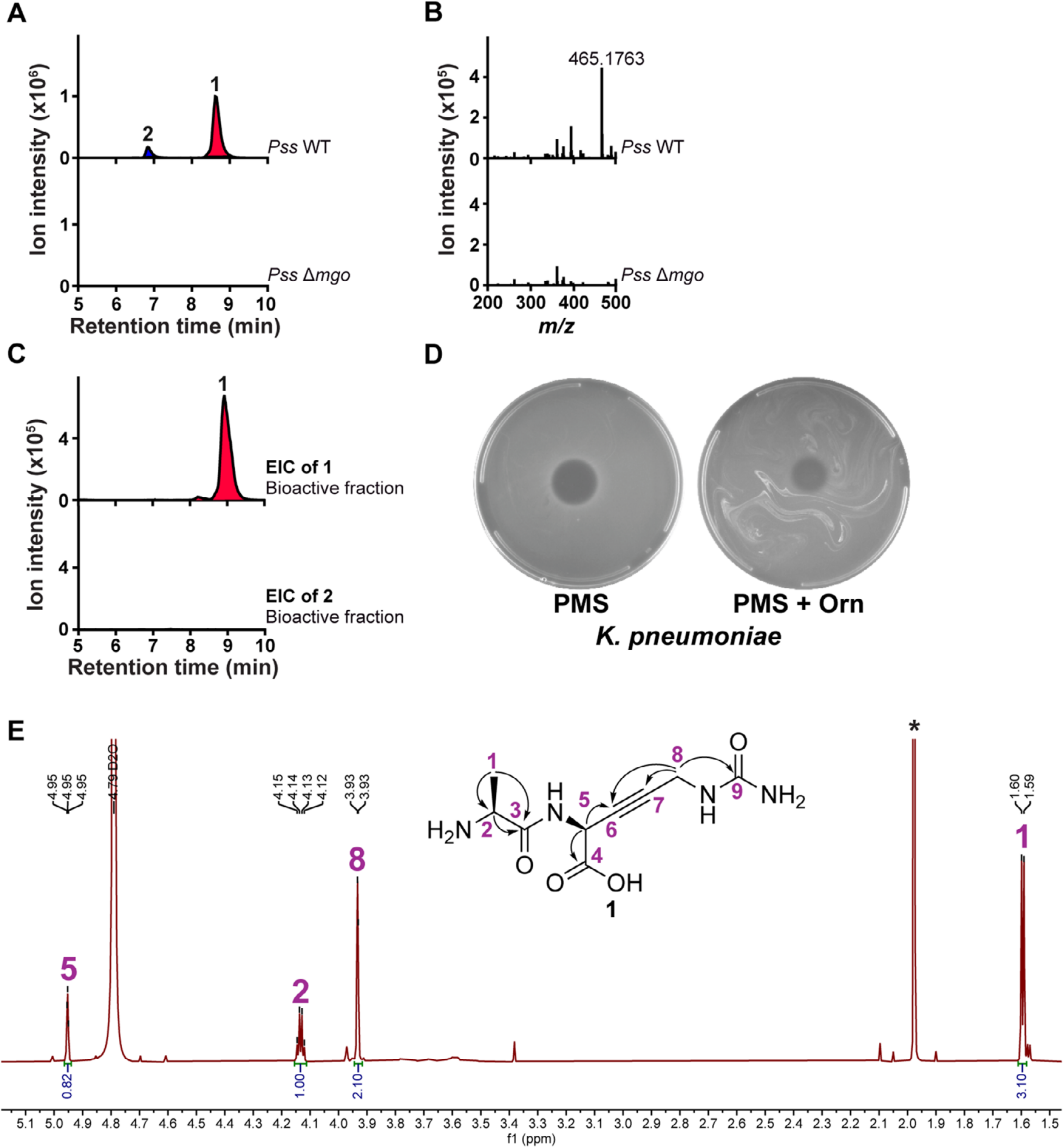
Mangotoxin is an alkyne‐containing alanine–citrulline dipeptide (Ala‐Cit_alkyne_). (A) Fmoc derivatization and LC‐HRMS analysis revealed the presence of two compounds of molecular masses 242.1015 and 241.1175 daltons in the supernatant of the mangotoxin‐producer *Pss* WT but not that of Δ*mgo*. Overlaid extracted ion chromatograms (EICs) for **1** (*m/z* 465.1769 [Fmoc‐M+H]^+^, red) and **2** (*m/z* 464.1928 [Fmoc‐M+H]^+^, blue) (B) Mass spectra at the retention time of 8.6 min showed that **1** (*m/z* 465.1769 [Fmoc‐M+H]^+^) was produced by *Pss* WT but not Δ*mgo*. (C) LC‐HRMS analysis of the bioactive fraction purified from the *Pss* supernatant demonstrated the presence of Ala‐Cit_alkyne_ (**1**, top) and absence of Ala‐Arg_alkyne_ (**2**, bottom). EICs of **1** and **2** are shown. (D) Isolated Ala‐Cit_alkyne_ inhibited the growth of *K. pneumoniae*, and the inhibition was partially reversed by supplementing the media with ornithine. Ala‐Cit_alkyne_ isolated from *Pss* was spotted on PMS agar plates where *K. pneumoniae* was grown in the absence (left) or presence (right) of ornithine. (E) Structure of the isolated Ala‐Cit_alkyne_ determined by one‐ and two‐dimensional NMR. Proton NMR is shown. (^1^H, ^13^C) HMBC correlations are depicted as arrows on the structure. The asterisk indicates acetate.

### 2.3 Isolation and Structure Elucidation of Mangotoxin

We isolated mangotoxin from *Pss* culture supernatant using bioactivity‐guided fractionation and determined its chemical structure. Culture supernatant was separated using reversed‐phase chromatography followed by hydrophilic interaction liquid chromatography (HILIC). Fractions from each purification were tested for antimicrobial activity in the *E. coli* inhibition assay, and the active fractions were analyzed using LC‐HRMS, pooled, and further purified. Consistently, **1** was identified as the dominant species in the active fractions (Figure 2C). Purified **1** inhibited the growth of *K. pneumoniae* and *E. coli*, and this inhibition was partially reversed by ornithine (Figures 2D and S4). The compound was analyzed using one‐ and two‐dimensional NMR spectroscopy (Figures 2E, S5–S8, and Table S2). A triplet proton peak at 4.95 ppm (H5) is shifted downfield from the α‐proton of a canonical amino acid, indicative of a modification at the β‐position (C6). The ^13^C NMR chemical shifts at 80.27 ppm (C6, β‐position) and 83.06 ppm (C7, γ‐position), and HMBC correlation of H5 to C6 indicate the presence of an alkyne between C6 and C7. The splitting pattern of H5 as a triplet is consistent with known long‐range homopropargyl coupling [32, 33]. Further, a doublet proton peak at 3.93 ppm (H8) is correlated to C6, C7, and a urea carbon at C9 in the HMBC spectrum; thus, the alkyne moiety is positioned in the citrulline side chain (Figures 2E and S8). The structural and activity data demonstrate that mangotoxin is an alanine–citrulline dipeptide containing an alkyne at the C_β_─C_γ_ position of citrulline (Ala‐Cit_alkyne_).

### 2.4 In Vitro Characterization of Mangotoxin Biosynthesis

Having determined the structure of mangotoxin from the native producer, we sought to elucidate its biosynthesis and characterize the functions of biosynthetic enzymes in vitro. We determined the substrate preference of the putative ATP‐grasp amide ligases MboC and MboD, which likely catalyze peptide bond formation in mangotoxin biosynthesis. ATP‐grasp enzymes activate the carboxylate of one substrate using ATP by forming an acyl phosphate intermediate [34, 35]. Nucleophilic attack on the acyl phosphate by the amine of the second substrate results in formation of an amide bond. Unlike adenylation domains in nonribosomal peptide synthetases, it is difficult to predict the substrate specificity of ligases based on their sequence or structure [36]. Thus, we developed a method to identify preferred substrates of purified MboC and MboD and their peptide products by LC‐HRMS in a one‐pot reaction containing the 20 canonical amino acids (Figures S9, S10, and S11). Analysis of the one‐pot MboC reaction mixture identified an alanine‐arginine dipeptide as the main product (Figure S10). Subsequent MS/MS analysis of this product confirmed its identity and demonstrated that alanine is on the N‐terminus (Figure S12). Similar analysis for MboC and MboD together in a one‐pot reaction using a mixture of amino acids as substrates showed that the main product contained an arginine, alanine, and leucine or isoleucine (Figure S11).

Formation of the alkyne likely results from four‐electron oxidation of a compound containing arginine or citrulline. To identify the enzymes responsible for catalyzing alkyne formation and understand the catalytic mechanism, we evaluated *mbo*‐encoded enzymes with predicted redox activities, namely MboA and MboB. MboA was initially annotated as a protein of unknown function [20]. Our query of the MboA amino acid sequence using InterProScan revealed that MboA is a member of the heme oxygenase‐like domain‐containing oxidases (HDOs) (IPR016084) [37]. AlphaFold 3 [38] model of MboA and alignment of the sequence with known HDOs suggest the presence of a canonical E‐H‐E‐H‐(D/E)‐H motif that binds a dinuclear metal cofactor (Figure S13). MboB harbors multiple domains capable of electron transfer: a [2Fe‐2S] ferredoxin‐like domain, a nicotinamide adenine dinucleotide (phosphate) (NAD(P)H)‐binding domain, and a flavin‐binding domain. An AlphaFold 3 [38] model of MboB was generated and used as a query for a Dali [39] search to identify structural homologs in the Protein Data Bank (PDB). A top hit was the crystal structure of phthalate dioxygenase reductase (PDB: 2PIA) (Figure S14). Phthalate dioxygenase reductase is the redox partner of a Rieske‐type dioxygenase, constituting a two‐component oxygenase involved in the degradation of aromatic compounds [40, 41]. The presence of similar domains in MboB as phthalate dioxygenase reductase suggests that MboB facilitates catalysis by MboA as the redox partner.

We tested the ability of MboA and MboB to catalyze alkyne formation in mangotoxin biosynthesis. Both proteins were overexpressed in *E. coli* and purified using a hexahistidine (MboA) or a maltose‐binding protein (MBP) tag (MboB) (Figure S9). Since Ala–Arg is the main product of the MboC reaction, the activity of MboA was tested toward Ala–Arg as a substrate. Incubation of MboA with synthetic Ala–Arg in the presence of ferrous iron and ascorbate yielded products that were 2 daltons less in mass than Ala–Arg (Figures 3A, 3B, S15, S16, and Table S3). These products eluted as a double peak by liquid chromatography and likely contained more than one species that resulted from a two‐electron oxidation of Ala–Arg (**3**). Inclusion of MboB and reduced nicotinamide adenine dinucleotide (NADH) in the MboA reaction led to the formation of a four‐electron oxidation product of Ala–Arg (Figure 3A, B). The product exhibited the same retention time as **2** extracted from *Pss* culture (Figure 3B).

**FIGURE 3:**
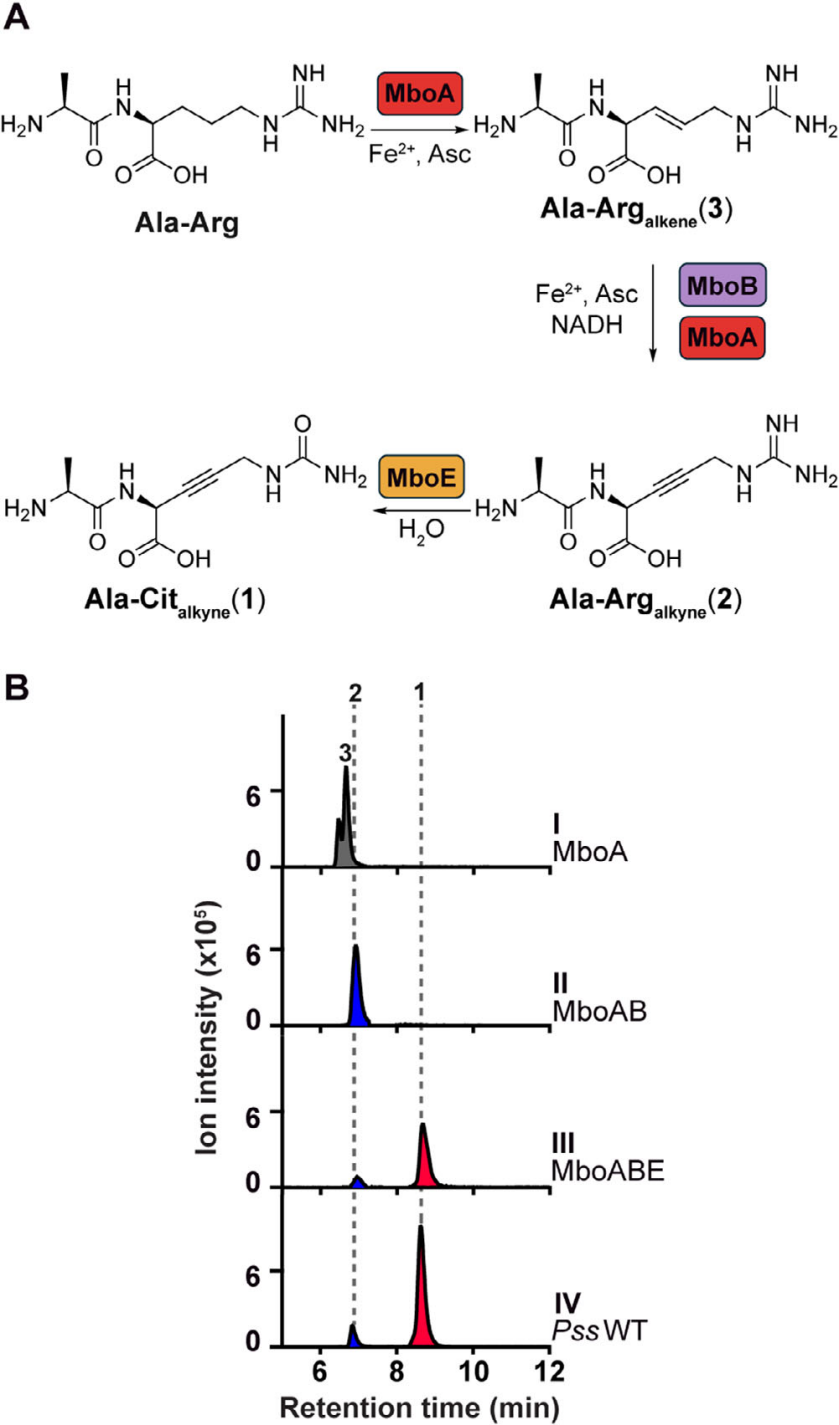
In vitro reconstitution demonstrates that MboA and MboB catalyze alkyne formation and MboE catalyzes deimination in the biosynthesis of Ala‐Cit_alkyne_ (**1**). (A) Reaction scheme for the conversion of Ala–Arg to Ala–Arg_alkene_ (**3**) by MboA and generation of Ala‐Arg_alkyne_ (**2**) in the presence of MboB. Compound **2** is subsequently transformed to **1** by MboE. Ferrous iron (Fe^2+^), ascorbate (Asc), and reduced nicotinamide adenine dinucleotide (NADH) were included in the reaction. (B) Fmoc derivatization and LC‐HRMS analysis of the in vitro reactions containing Mbo enzymes and EICs of the products. (I) Incubation of MboA with Ala–Arg formed a two‐electron oxidation product that likely corresponds to Ala–Arg_alkene_ (**3**, *m/z* 466.2085 [Fmoc‐M+H]^+^). (II) Incubation of both MboA and MboB with Ala–Arg generated **2** (*m/z* 464.1928 [Fmoc‐M+H]^+^). (III) Addition of MboE to the MboA and MboB reaction converted **2** to **1**. (IV) Compounds **1** and **2** were detected in *Pss* WT supernatant. Overlaid EICs for **1** (*m/z* 465.1769 [Fmoc‐M+H]^+^, red) and **2** (*m/z* 464.1928 [Fmoc‐M+H]^+^, blue) are shown in (III) and (IV).

We used deuterium‐labeled arginine ([2,3,3,4,4,5,5‐D_7_]‐l‐Arg) to further characterize the oxidation products and the mechanism of the MboA and MboB reaction. The Ala‐[D_7_]‐Arg dipeptide was first generated using MboC, then the reaction mixture was incubated with MboA and MboB. Two‐ and four‐electron oxidation of Ala‐[D_7_]‐Arg resulted in the loss of two and four deuterium atoms, respectively (Figure S17). The loss of deuterium demonstrates that the oxidations occur on the carbon chain of arginine, resulting in an alkene (Ala‐[D_5_]‐Arg_alkene_, **3**) and an alkyne (Ala‐[D_3_]‐Arg_alkyne_, **2**) in a stepwise manner. The observation that five deuterium atoms were retained in **3** and three deuterium atoms were retained in **2** indicates that no isomerization steps involving water have occurred during the reaction and that both the alkene and alkyne are positioned at the C_β_─C_γ_ of the arginine side chain.

The structure of mangotoxin (**1**) differs from that of **2** at the side chain of citrulline. Modeling of MboE using AlphaFold and search for structural homologs using Dali identified conserved residues involved in arginine binding and catalytic residues involved in arginine deimination (Figure S18). While most known bacterial deiminases modify free arginine, peptidylarginine deiminase from the human pathogen *Porphyromonas gingivalis* catalyzes citrullination of proteins and peptides [42]. We proposed that MboE converts **2** to mangotoxin (**1**). Indeed, incubation of **2** produced from the MboA and MboB reaction with MboE led to formation of **1**, which exhibited the same retention time as mangotoxin extracted from *Pss* (Figure 3A, B).

The recent report on the in vitro reconstitution of homologous Mbo enzymes from *P. syringae* PDD‐32b‐74 showed that MboC and MboD catalyzed the formation of the Leu–Ala–Arg tripeptide [27]. The tripeptide was modified by MboA and MboB to Leu–Ala–Arg_alkyne_ (**4**) and further converted to Leu–Ala–Cit_alkyne_ by MboE [27]. Our substrate screen also showed that the amide ligases MboC and MboD together preferentially generated a peptide containing alanine, arginine, and leucine or isoleucine (Figure S11). Therefore, we also tested synthetic Leu–Ala–Arg as a substrate for MboA, MboB, and MboE (Figures S19, S20, and Table S4). The Leu–Ala–Cit_alkyne_ product was detected in the three‐enzyme reaction (Figure S21). Further, minimal Leu–Ala–Arg_alkyne_ intermediate was accumulated in the reaction, suggesting that Leu–Ala–Arg_alkyne_ was fully converted to Leu–Ala–Cit_alkyne_ by MboE. However, Leu–Ala–Cit_alkyne_ was not detected in the native producer *Pss* under the culturing conditions. Thus, the complete biosynthesis of mangotoxin in the producer likely involves the assembly of a tripeptide by MboC and MboD, subsequent alkyne formation catalyzed by MboA and MboB, followed by MboE‐catalyzed deimination of the C‐terminal arginine to citrulline and the proteolytic removal of the N‐terminal amino acid to yield the Ala‐Cit_alkyne_ dipeptide (Figure 4A).

**FIGURE 4:**
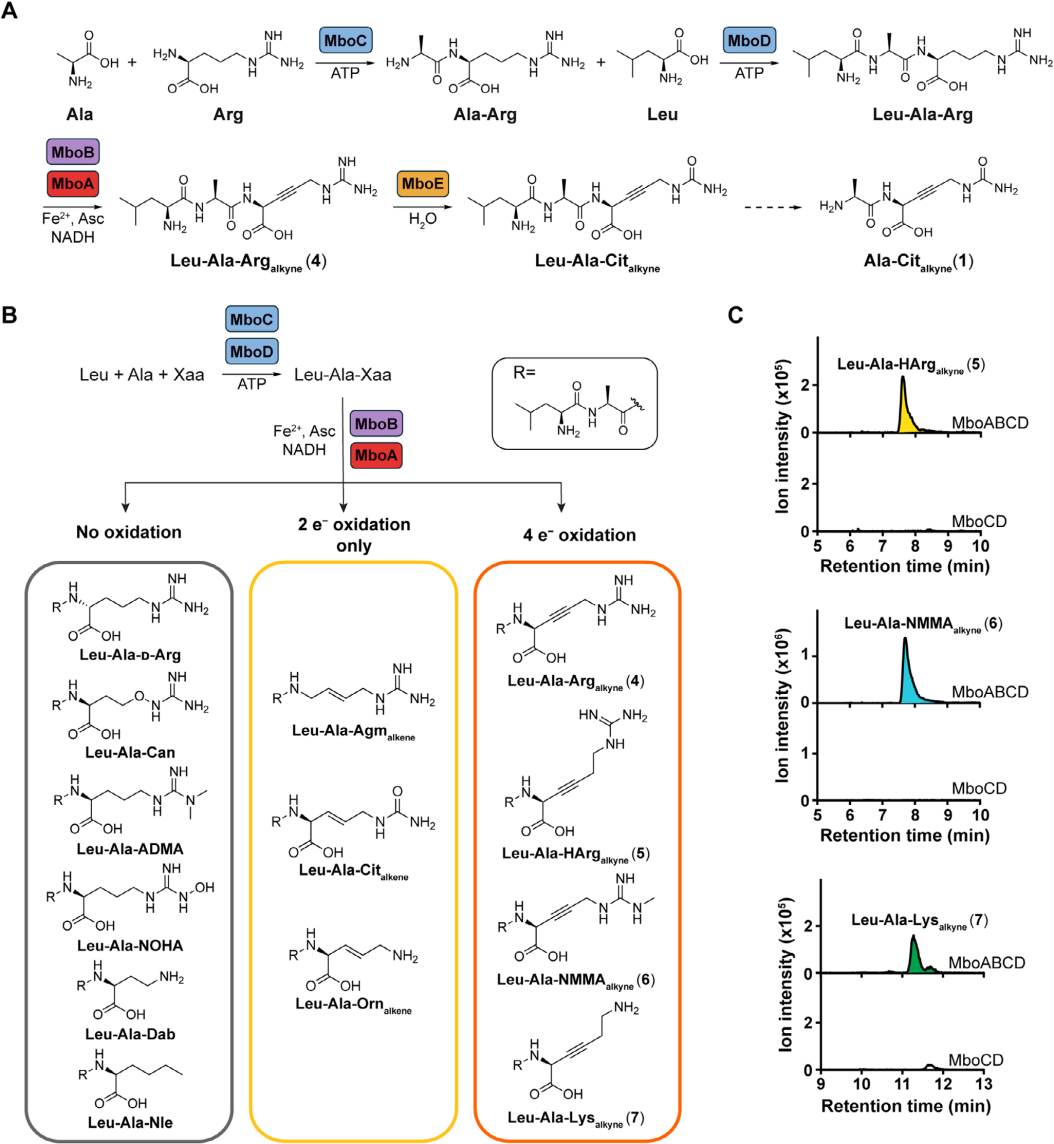
Mbo enzymes can catalyze the formation of various oxidized Leu–Ala–Xaa tripeptides in vitro that likely contain an alkene or alkyne. (A) Proposed mangotoxin biosynthesis pathway in the native producer *P. syringae*. (B) Reaction scheme catalyzed by Mbo enzymes in vitro and proposed structures of non‐native products from these reactions. d‐Arg, d‐arginine; Can, l‐canavanine; ADMA, *N* ^G^, *N* ^G^‐dimethyl‐l‐arginine (asymmetric dimethylarginine); NOHA, *N* ^G^‐hydroxy‐l‐arginine; Dab, l‐2,4‐diaminobutyric acid; Nle, l‐norleucine; Agm, agmatine; Cit, l‐citrulline; Orn, l‐ornithine; Arg, l‐arginine; HArg, l‐homoarginine; NMMA, *N* ^G^‐monomethyl‐l‐arginine; Lys, l‐lysine. Adenosine triphosphate (ATP), iron (Fe^2+^), ascorbate (Asc), and reduced nicotinamide adenine dinucleotide (NADH) were included in the reactions. (C) Fmoc derivatization and LC‐HRMS analysis of the MboC and MboD reactions subsequently incubated with MboA and MboB (MboABCD) or of the MboC and MboD control reactions (MboCD). EICs of the four‐electron oxidation products that likely correspond to the alkyne are shown, including Leu–Ala–HArg_alkyne_ (**5**, *m/z* 591.2926 [Fmoc‐M+H]^+^, yellow), Leu–Ala–NMMA_alkyne_ (**6**, *m/z* 591.2926 [Fmoc‐M+H]^+^, blue), and Leu–Ala–Lys_alkyne_ (**7**, *m/z* 771.3388 [(bis‐Fmoc)‐M+H]^+^, green). Reactions and analysis were repeated on a separate day with similar results.

### 2.5 Mbo Enzymes Can Catalyze the Formation of Multiple Non‐Native Tripeptides and Oxidation of the C‐Terminal Amino Acid

We investigated the substrate scope of MboA and MboB using the amide ligases MboC and MboD to generate a panel of tripeptides that contain different amino acids at the C‐terminus (Leu–Ala–Xaa; Xaa, variable amino acid). The variable amino acid included *N* ^G^, *N* ^G^‐dimethyl‐l‐arginine (asymmetric dimethylarginine, ADMA), *N* ^G^‐hydroxy‐l‐arginine (NOHA), *N* ^G^‐monomethyl‐l‐arginine (NMMA), d‐arginine (d‐Arg), l‐citrulline (Cit), agmatine (Agm), l‐canavanine (Can), l‐homoarginine (HArg), l‐ornithine (Orn), l‐lysine (Lys), l‐2,4‐diaminobutyric acid (Dab), and l‐norleucine (Nle). The variable amino acid, l‐leucine, and l‐alanine were incubated with MboC and MboD and reactions were analyzed by LC‐HRMS after derivatization using Fmoc‐Cl. The expected Leu–Ala–Xaa tripeptide products were detected for all tested substrates for MboC and MboD (Figures S22, S23).

The Leu–Ala–Xaa tripeptides produced in the MboC and MboD reactions were then incubated with MboA and MboB. LC‐HRMS analysis showed that MboA and MboB catalyzed the conversion of Leu–Ala–HArg, Leu–Ala–NMMA, and Leu–Ala–Lys to the four‐electron oxidation products (**5**, **6**, and **7**, respectively) (Figure 4B, C). MS fragmentation analysis confirmed the oxidation to be located on the variable C‐terminal amino acid, HArg, NMMA, or Lys (Figures S24–S26). The retention time shift of the oxidized product from the substrate was similar to the difference between Leu–Ala–Arg_alkyne_ and Leu–Ala–Arg and consistent with alkyne formation (Figures 4C, S22, and S23). Only the two‐electron oxidized species for the tripeptides, including Leu–Ala–Agm, Leu–Ala–Cit, and Leu–Ala–Orn, were detected, which likely correspond to the alkene (Figures 4B and S23). No MboA and MboB‐dependent oxidation products were observed for Leu–Ala‐d–Arg, Leu–Ala–Can, Leu–Ala–ADMA, Leu–Ala–NOHA, Leu–Ala–Dab, or Leu–Ala–Nle (Figure 4B).

Since the initial report of mangotoxin bioactivity over 20 years ago, we elucidated the chemical structure of this phytotoxin from the native producer, *Pss* UMAF0158. Identification of mangotoxin was achieved by comparative metabolomic analysis using high‐resolution mass spectrometry and bioactivity‐guided fractionation. We determined that mangotoxin is an alanine–citrulline dipeptide containing an alkyne at C_β_─C_γ_ of citrulline. Its inhibition of *E. coli* growth and reversal of inhibition by ornithine is consistent with the reported bioactivity of the producing bacterium *Pss* [14]. In vitro reconstitution of mangotoxin biosynthesis further supports mangotoxin being a product of *mbo*‐encoded enzymes. While an alkyne‐containing alanine–arginine dipeptide (Ala–Arg_alkyne_) was also detected in the *Pss* culture supernatant and the in vitro biosynthetic reaction, this metabolite was a minor species in the supernatant and eluted at a different retention time from the fraction exhibiting mangotoxin activity; therefore, Ala–Arg_alkyne_ is likely a biosynthetic intermediate or proteolytic product. Other minor species observed in *Pss* include ions that correspond to standalone Arg_alkyne_ and Cit_alkyne_ (Figure S27 and Table S1), which may be proteolytic products of Ala–Arg_alkyne_ and mangotoxin, respectively.

The structure of mangotoxin is unique among peptide toxins produced by plant‐associated bacteria. These toxins are usually di‐ or tri‐peptides containing various noncanonical amino acids or other moieties, such as sulfodiaminophosphinyl‐ornithine in phaseolotoxin [43], β‐lactam in tabtoxin [5], phosphinothricin in bialophos [44], epoxysuccinate in dapdiamides [45], and amidinospermidine in phevamine A [26]. These noncanonical moieties enable inhibition of bacterial or plant enzymes by directly reacting with active site residues or mimicking enzyme substrates. The acetylene moiety in mangotoxin may react with nucleophilic residues in the active sites of target enzymes. Mangotoxin has been proposed to inhibit the ornithine‐forming step in arginine biosynthesis that is catalyzed by the ornithine *N*‐acetyltransferase *in planta* or the *N*‐acetylornithine deacetylase in *E. coli* [14]. In addition to inhibiting *E. coli*, we found that mangotoxin also inhibits multidrug‐resistant *K. pneumoniae*. Our expanded analysis of *mbo* genes in all *Pseudomonas* showed that homologs of *mboA*‐*F* are present in 335 organisms (10%) within the *Pseudomonas syringae* group (taxon ID: 136849) but are present in few other *Pseudomonas* species (Figure S28, Supporting File 2). This observation suggests that mangotoxin is primarily distributed in plant pathogenic bacteria. Our identification and structural elucidation of mangotoxin will facilitate efforts to understand the mode of action of this phytotoxin and antimicrobial.

Alkyne formation in mangotoxin biosynthesis requires a novel enzyme pair. Alkyne‐containing natural products have attracted significant attention due to their diverse biological activities and potential use of their biosynthetic enzymes to install alkyne groups in natural products and proteins for bioorthogonal chemistry [46, 47]. We show that a heme oxygenase‐like domain‐containing oxidase (HDO), MboA, catalyzes the oxidation of a C_β_─C_γ_ single bond to a triple bond with the aid of a multidomain protein, MboB. Our work corroborates the findings from the recent report on in vitro reconstitution of MboA and MboB from a different *P. syringae* strain [27]. This enzyme pair differs from other alkyne‐forming enzymes, such as membrane‐bound desaturases in fatty acid and polyketide biosynthesis [47], a pyridoxal phosphate‐dependent enzyme in terminal alkyne amino acid biosynthesis [48], and cytochrome P450 enzymes in meroterpenoid biosynthesis [49, 50].

HDOs catalyze diverse oxidative transformations on amino acids [51]. MboB is required for full four‐electron oxidation by MboA of Leu–Ala–Arg to Leu–Ala–Arg_alkyne_, which is further converted to mangotoxin. The presence of a [2Fe‐2S] ferredoxin domain, an FAD‐binding domain, and an NAD(P)H‐binding domain in MboB is reminiscent of the multi‐component Rieske oxygenase systems, which contain two to four components comprising a flavin reductase, a ferredoxin, the Rieske oxygenase, and an optional effector subunit [52, 53]. The biosynthetic gene clusters of streptozotocin and azomycin encode ferredoxin‐like proteins that are putative electron‐transfer proteins for the HDOs SznF and RohS, respectively [54, 55, 56]. While functions of these ferredoxin‐like proteins have not been characterized, synthetic electron transfer systems containing phenazine methosulfate and NADH were sufficient to enable multiple turnovers by most HDOs characterized to date, including SznF and RohS. A few other HDOs, such as FlcD and FlcE in fluopsin C biosynthesis, catalyze multiple turnovers without the need for external reductants [57, 58]. An estimated 40% of HDO‐containing biosynthetic gene clusters encode a putative electron‐transfer protein [51]. Further characterization of the interaction between MboA and MboB will aid in understanding how the metal cofactors of HDOs are regenerated under physiological conditions and in identifying the functions of HDOs that require dedicated redox partners for activity.

Mangotoxin isolated from the native producer *Pss*, Ala–Cit_alkyne_, does not contain the amino acid ligated by MboD. However, genetic studies showed that *mboD* is required in *Pss* for mangotoxin‐dependent antimicrobial activity [20]. This observation suggests mangotoxin biosynthesis involves a tripeptide intermediate, which is supported by the in vitro biosynthesis of Leu–Ala–Cit_alkyne_ tripeptide using purified Mbo enzymes in this study and the recent, parallel report [27]. Our findings suggest that the N‐terminal amino acid of the modified tripeptide is likely removed in *Pss* to generate the mature mangotoxin dipeptide. Removal of an N‐terminal amino acid was also reported in the biosynthesis of the antibiotic, l‐2‐amino‐4‐methoxy‐*trans*‐3‐butenoic acid (AMB), by *Pseudomonas aeruginosa*, where an Ala‐AMB dipeptide is made en route to the single amino acid AMB [59]. The role of the amino acid ligated by MboD in mangotoxin biosynthesis and its relation to bioactivity remains to be elucidated. Removal of the N‐terminal amino acid may occur before or after transport by the putative transporter MboF.

ATP‐grasp enzymes exhibit a wide substrate scope and have been used to couple non‐natural carboxylates and amines in peptide bonds [60, 61, 62]. We showed that the amide ligases MboC and MboD can accommodate various canonical and noncanonical amino acids, including arginine derivatives such as homoarginine, agmatine, and canavanine, as well as other positively charged amino acids containing an amine group such as lysine, ornithine, and 2,4‐diaminobutyric acid. The substrate permissiveness of MboC and MboD was leveraged to synthesize Leu–Ala–Xaa tripeptides for characterizing the substrate scope of the alkyne‐forming enzyme pair MboA and MboB.

Our substrate scope studies revealed key moieties of the Xaa side chain required for alkyne formation by MboA and MboB. The enzyme pair can accommodate small variations to the guanidine moiety, such as a single *N* ^G^‐methyl group (*N*‐monomethylarginine) or chain length extension to homoarginine. However, changing the steric or electronic properties of the guanidine, as in asymmetric dimethylarginine, *N*‐hydroxyarginine, citrulline, or changing to the hydrophobic residue norleucine, is not preferred. The inability to convert tripeptides containing agmatine, which is missing the carboxylate, or containing d‐Arg, indicates that the presence and stereochemistry of the carboxylate are important for catalysis. In fact, the recently published substrate‐ and Fe (II)‐bound crystal structure of MboA (PDB: 9YPM) showed that the Arg carboxylate at the C‐terminus of the tripeptide substrate binds iron, supporting a key role of the carboxylate in cofactor binding and catalysis. Swapping the guanidine group for an amine group in the Xaa side chain is also tolerated, but the four‐carbon chain in lysine is preferred over the three‐carbon chain in ornithine or the two‐carbon chain in 2,4‐diaminobutyric acid. Introducing a heteroatom at C_δ_ (canavanine) stops the reaction, suggesting that the catalysis starts at the Cγ of the substrate. Overall, the ability of MboA and MboB to catalyze alkyne formation on various substrate analogues render them attractive candidates for engineering to generate novel alkyne‐containing molecules.

## 3 Conclusion

We demonstrated that mangotoxin produced by the plant pathogenic bacterium *P. syringae* is a rare alkyne‐containing alanine–citrulline dipeptide. The citrulline‐alkyne moiety likely plays a key role in the inhibition of arginine biosynthesis in plants infected by *Pss* and of other bacteria in the plant microbiome. Our work will enable efforts to elucidate the role of mangotoxin in the development of plant diseases caused by various *P. syringae* strains. Activity of mangotoxin against a multidrug‐resistant human pathogen highlights the potential of phytotoxins that target metabolic pathways as antimicrobial leads. Complete biosynthesis of mangotoxin in the producer likely involves two ATP‐grasp enzymes for peptide assembly, an enzyme pair for alkyne formation, deiminase for citrullination, and an unknown proteolytic enzyme to remove the N‐terminal leucine. Together with the recent functional and structural characterization of a homologous enzyme pair in a parallel study, our work sets the foundation for understanding the catalytic mechanism of MboA and MboB for acetylenation of an unactivated carbon‐carbon bond. Our findings on the substrate scope of the mangotoxin biosynthetic enzymes expand the biocatalytic toolbox for peptide coupling and alkyne formation and provide a blueprint for engineering Mbo enzymes to generate non‐native alkyne‐containing molecules. Mangotoxin analogues generated by the Mbo enzymes will facilitate structure–activity relationship studies to understand mangotoxin mode of action and to improve antimicrobial activity. MboA and MboB may also be used as a guide to discover other alkyne‐containing natural products.

## Author Contributions

**Alexis H. Murray**: conceptualization, methodology, validation, writing – review and editing, writing – original draft, investigation, data curation, visualization. **Sohan Hazra**: methodology, validation, writing – review and editing, investigation, data curation, visualization. **Ryan W. Mull**: methodology, writing – review and editing, data curation. **Erinn M. O'Neill**: methodology, writing – review and editing, data curation. **Bo Li**: conceptualization, funding acquisition, writing – original draft, investigation, writing – review and editing, supervision, project administration, resources, methodology.

## Conflicts of Interest

The authors declare no conflicts of interest.

## Supporting information




**Supporting File 1**: anie74663‐sup‐0001‐SuppMat.pdf.

**Supporting File 2**: anie74663‐sup‐0002‐SuppMat.xlsm.

## Data Availability

The data that supports the findings of this study are available in the supplementary material of this article
