# Supplementary material for: Structure and Biosynthesis of Mangotoxin Produced by the Plant Pathogen Pseudomonas syringae
Source: Angew Chem Int Ed Engl. 2026 Sep 22;65(45):e2211678. doi: 10.1002/anie.2211678 (PMC13725569; doi:10.1002/anie.2211678)
Supplement: Supplementary file 1 [file ANIE-65-e2211678-s001.pdf]

Supplemental information

Structure and Biosynthesis of Mangotoxin Produced by the Plant Pathogen *Pseudomonas syringae*

Alexis H. Murray, Sohan Hazra, Ryan W. Mull, Erinn M. O'Neill, Bo Li

Department of Chemistry, University of North Carolina at Chapel Hill, Chapel Hill, NC 27599

Corresponding author: Bo Li (boli@email.unc.edu)

Experimental Procedures:

Materials 4
General information and instrumentation 4
General method for liquid chromatography-mass spectrometry analysis 4
Fmoc derivatization for liquid chromatography-mass spectrometry analysis 5
Strain and culture conditions for mangotoxin production 5
Inhibition assay for mangotoxin detection 5
Comparative metabolomics analysis of *Pss* WT and Δ*mgo* 5
Bioactivity-guided fractionation 5
Isolation of Ala-Cit$_{alkyne}$ from *Pss* 6
Accession codes of Mbo enzymes 7
Cloning of *mboA*, *mboC*, *mboD*, and *mboE* 7
Cloning of *mboB* 7
Expression and purification of MboA, MBP-MboB, MboC, MboD, and MboE 7
Bioinformatic analysis of MboA, MboB, and MboE 8
Reconstitution of MboC and MboD *in vitro* 8
Synthesis of Leu-Ala-Arg and Ala-Arg 9
MboA and MboC one-pot reaction 10
MboC reaction of Ala and D_7-Arg followed by MboA/MboB reaction 10
One-pot reaction containing MboA, MboB, MboC, and MboE 10
Testing the substrate scope of MboA and MboB 11
Phylogenetic analysis of the *Pseudomonas syringae* group and identification of *mboA-F* genes in *Pseudomonas* 11

Supplementary Tables:

Table S1. Top comparative metabolomics hits of ions present in *Pss* WT and not Δ*mgo* with proposed structures. 12
Table S2. NMR shifts and 2D correlations of isolated Ala-Cit$_{alkyne}$. 14
Table S3. NMR shifts of synthesized Ala-Arg. 14
Table S4. NMR shifts of synthesized Leu-Ala-Arg. 15
Table S5. Gene sequences of *mboA*, *mboC*, *mboD*, *mboE*, and codon-optimized *mboB*. 15
Table S6. List of primers used in this study. 18

Supplementary Figures:

Figure S1. *E. coli* MG1655 grown on agar plates was inhibited by cultures of *Pss* WT but not Δ*mgo*. 18
Figure S2. Multiple ions were identified in *Pss* WT but not the Δ*mgo* control. 19
Figure S3. Tandem mass spectrum of Fmoc-Ala-Cit$_{alkyne}$ fragmented at 10 eV collision energy. 19
Figure S4. Inhibition of *E. coli* BL21 by isolated mangotoxin (Ala-Cit$_{alkyne}$). 20
Figure S5. ^{1}H NMR spectrum (850 MHz) of Ala-Cit$_{alkyne}$ isolated from *Pss* UMAF0158 in D_2O 21
Figure S6. ^{13}C NMR spectrum (850 MHz) of Ala-Cit$_{alkyne}$ isolated from *Pss* UMAF0158 in D_2O 22
Figure S7. ^{1}H, ^{13}C-HSQC NMR spectrum (850 MHz) of Ala-Cit$_{alkyne}$ isolated from *Pss* UMAF0158 in D_2O. 23

Figure S8. ^{1}H, ^{13}C-HMBC NMR spectrum (850 MHz) of Ala-Cit_{alkyne} isolated from *Pss* UMAF0158 in D_2O. 24
Figure S9. SDS-PAGE gel of purified MboA, MBP-MboB, MboC, MboD, and MboE at 5 μM. 25
Figure S10. MboC preferentially catalyzes the formation of the alanine and arginine-containing dipeptide. 25
Figure S11. MboC and MboD preferentially catalyze the formation of a tripeptide consisting of alanine, arginine, and leucine or isoleucine. 26
Figure S12. Tandem mass spectrum of Fmoc-Ala-Arg fragmented at 10 eV collision energy. 27
Figure S13. AlphaFold 3 model of MboA complexed with two Fe^{3+} (orange, left). 28
Figure S14. AlphaFold 3 model of MboB complexed with Fe^{3+} and flavin adenine dinucleotide (FAD) (left). 28
Figure S15. ^{1}H NMR spectrum (600 MHz) of Ala-Arg in D_2O. 29
Figure S16. ^{13}C NMR spectrum (600 MHz) of Ala-Arg in D_2O. 30
Figure S17. Oxidation of L-Ala-[D_7]-Arg by MboA and MboB resulted in the loss of two or four deuteriums that is consistent with alkene or alkyne products, respectively. 31
Figure S18. Dali search for structural homologs of MboE and sequence alignment. 32
Figure S19. ^{1}H NMR spectrum (600 MHz) of Leu-Ala-Arg in D_2O. 33
Figure S20. ^{13}C NMR spectrum (600 MHz) of Leu-Ala-Arg in D_2O. 34
Figure S21. The synthetic Leu-Ala-Arg tripeptide is also a substrate of MboA, MboB, and MboE. 35
Figure S22. MboC and MboD catalyzed the formation of different Leu-Ala-containing tripeptides. 36
Figure S23. Two-electron oxidation products were detected in the MboA and MboB reactions containing different tripeptide substrates. 37
Figure S24. Tandem mass spectrum of the proposed Fmoc-Leu-Ala-Lys_{alkyne} product. 38
Figure S25. Tandem mass spectrum of the proposed Fmoc-Leu-Ala-$HArg_{alkyne}$ product. 39
Figure S26. Tandem mass spectrum of the proposed Fmoc-Leu-Ala-$NMMA_{alkyne}$ product. 40
Figure S27. Mass spectrum of purified Ala-Cit_{alkyne} after Fmoc derivatization from LC-MS analysis.. 40
Figure S28. The distribution of the *mangotoxin biosynthetic operon* (*mbo*) in 3071 organisms of *Pseudomonas syringae* group (taxon ID: 136849). 41

<u>Supporting References</u>

Experimental Procedures:

Materials

Chemicals were purchased from commercial suppliers and used without further purification. 2-[4-(2-Hydroxyethyl)piperazin-1-yl]ethanesulfonic acid (HEPES) was purchased from GoldBio. Deuterium oxide (D_2O) was purchased from Cambridge Isotopes Laboratories. 1-[Bis(dimethylamino)methylene]-1H-1,2,3-triazolo[4,5-b]pyridinium 3-oxide hexafluorophosphate (HATU) was purchased from Combi-Blocks. Ammonium iron(II) sulfate hexahydrate [$(NH_4)_2Fe(SO_4)_2 \cdot 6H_2O$] and dichloromethane (DCM) were purchased from Acros Organics. Imidazole and dimethylformamide (DMF) were purchased from Thermo Scientific. Chloroform was purchased from VWR. Ammonium phosphate monobasic ($NH_4H_2PO_4$), potassium chloride (KCl), spectinomycin dihydrochloride pentahydrate (Sm), methanol, ornithine monohydrochloride (Orn), trifluoroacetic acid (TFA), adenosine 5′-triphosphate disodium salt hydrate (ATP), nicotinamide adenine dinucleotide reduced dipotassium salt (NADH), thionyl chloride, triethylsilane (TES), piperidine, *N*,*N*-diisopropylethylamine (DIPEA), 9-fluorenylmethyl chloroformate (Fmoc), and amino acids were purchased from Sigma-Aldrich. L-Citrulline and agmatine sulfate were purchased from Alfa Aesar. L-Canavanine sulfate was purchased from ChemCruz. L-Norleucine and D-arginine were purchased from MedChemExpress. Magnesium sulfate heptahydrate ($MgSO_4 \cdot 7H_2O$), iron(III) chloride hexahydrate ($FeCl_3 \cdot 6H_2O$), potassium phosphate dibasic (K_2HPO_4), dextrose, Bacto Proteose Peptone No. 3, glycerol, acetonitrile, formic acid, Luria broth (LB), ampicillin sodium salt (Amp), chloramphenicol (Cm), kanamycin sulfate (Kan), sodium chloride (NaCl), isopropylthio-D-galactoside (IPTG), sodium ascorbate, sodium hydroxide (NaOH), and Fmoc-L-Arg(Pbf)-OH were purchased from Fisher Scientific. Restriction enzymes and chemically competent *E. coli* cells were purchased from New England Biolabs. L-Arginine-[2,3,3,4,4,5,5-D_7] monohydrochloride was obtained from Cayman Chemicals.

General information and instrumentation

Reversed-phase chromatography was performed on C18-bonded silica columns, including Gemini (Phenomenex) for analytical high-performance liquid chromatography (HPLC) and Luna (Phenomenex) for preparative HPLC (prep HPLC). Prep HPLC was performed on a Varian Prostar with 330 PDA variable wavelength detector. Semi-preparative HPLC was performed on a Luna (Phenomenex) hydrophilic interaction liquid chromatography (HILIC) column on a Shimadzu HPLC equipped with an SPD-20A UV/Vis detector. NMR spectra were collected on a Bruker Avance III HD 850 MHz NMR spectrometer or the 600 MHz Bruker AVIII spectrometer, and data were analyzed in MestReNova. High-resolution mass spectra (HRMS) were obtained on an Agilent 1290/6546 ultra high-performance liquid chromatography-quadrupole time-of-flight mass spectrometer (UHPLC-QToF-MS).

General method for liquid chromatography-mass spectrometry analysis

Samples were injected on the Gemini (Phenomenex) C18 column (5 µm, 50 x 2.00 mm) and analyzed by liquid chromatography coupled to high-resolution mass spectrometry (LC-HRMS). Samples were run at 0.2 mL/min using mobile phase A (water with 0.1% formic acid) and mobile phase B (acetonitrile with 0.1% formic acid). The column was equilibrated at 2% B for 2 min, and the analytes were separated using a gradient of 2–98% B from 2 to 12 min. The column was cleaned at 98% B for 2 min, switched to 2% B over a minute, and re-equilibrated at 2% B for 2 min. Mass spectra were obtained in positive electrospray ionization (ESI) mode using a gas temperature of

320 °C, drying gas flow rate of 12 L/min, capillary voltage of 3500 V, nebulizer pressure of 35 psi, fragmentor voltage of 175 V, and skimmer voltage of 65 V.

Fmoc derivatization for liquid chromatography-mass spectrometry analysis

For samples that were derivatized using Fmoc before LC-MS analysis, 50 µL of sample was mixed with 25 µL of 200 mM sodium borate at pH 10.4 and 25 µL of 4.5 g/L Fmoc-Cl in acetonitrile. The reactions were incubated at room temperature for 10 min and centrifuged for 10 min at 20,000 relative centrifugal force (rcf) to separate precipitants. From the supernatant of Fmoc-derivatized reactions, 10 µL samples were injected and analyzed using the general LC-MS analysis method.

Strain and culture conditions for mangotoxin production

The *mgo* markerless knockout in *Pseudomonas syringae* pv. *syringae* UMAF0158 (*Pss* hereafter) was generated previously and validated by PCR.[1] *Pss* WT and Δ*mgo* were grown in King's Broth (KB) (20 g/L Bacto Proteose Peptone No. 3, 1.5 g/L K_2HPO_4, 1.5 g/L $MgSO_4 \cdot 7H_2O$, 1% (v/v) glycerol, pH 7.0) at 30 °C with shaking at 225 rpm overnight. A sample of 500 µL of overnight seed culture was used to inoculate 500 mL *Pseudomonas* minimal salts (PMS) media (1 g/L $NH_4H_2PO_4$, 0.2 g/L KCl, 0.2 g/L $MgSO_4 \cdot 7H_2O$, 100 µM $FeCl_3 \cdot 6H_2O$, 2 g/L dextrose, pH 7.0) and cultures were grown with shaking at 225 rpm at 22 °C for 3 days.

Inhibition assay for mangotoxin detection

The *Escherichia coli* inhibition assay was adapted for detecting *Pseudomonas* antimetabolite toxins.[2] Cultures of *E. coli* or *Klebsiella pneumoniae* ATCC BAA-2146 were prepared in 5 mL of LB and were grown at 37 °C with shaking at 225 rpm overnight. Overnight cultures were centrifuged at 4,000 rcf, resuspended in PMS media, and added to PMS molten agar (15 g/L agar) supplemented with or without 30 mM L-ornithine at a density of 10^6 cells/mL. The *E. coli*-containing molten agar was poured into plates. Test samples were added to plates, and plates were incubated at 30 °C overnight.

Comparative metabolomics analysis of *Pss* WT and Δ*mgo*

Pss WT and Δ*mgo* cultures were prepared in triplicate and centrifuged at 4,000 rcf, and the supernatant was filtered through a 0.45 µm syringe filter to remove residual cells. The filtered supernatant was tested directly for *E. coli* inhibition or Fmoc-derivatized for LC-MS analysis. Data were exported and triplicate samples were uploaded to the XCMS server for pairwise analysis comparing WT and Δ*mgo* samples.[3] Ions unique to the WT samples were analyzed by MS/MS using the LC parameters in the general LC-MS method. Targeted MS/MS analyses were performed at collision energies of 10, 20, and 40 eV.

Bioactivity-guided fractionation

Cultures of *Pss* WT and Δ*mgo* in 50 mL PMS were centrifuged at 4,000 rcf, and each supernatant was filtered using a 0.45 µm syringe filter. An equal volume of acetonitrile was added to the filtered supernatant, samples were centrifuged at 4,000 rcf, and the resulting supernatant was transferred to a round bottom flask and dried by rotary evaporation at 30 °C. Samples were resuspended in 2% acetonitrile in water and spun at 20,000 rcf, then purified using a prep HPLC on the Luna C18 column (10 µm, 250 x 21.2 mm). The prep HPLC flow rate was 12 mL/min, and the mobile phases included A (water with 0.1% formic acid) and B (acetonitrile with 0.1% formic acid). In the LC method, 2% B was held for 5 min followed by a gradient of 2–20% B over 5 min, and 20% B was

held for 20 min. Then a gradient of 20–98% B over 15 min was used, and 98% B was held for 5 min before the column was re-equilibrated from 98% to 2% B over 7 min. All prep HPLC fractions were collected in 1-min intervals, dried down overnight in a Genevac benchtop evaporator (Scientific Products), resuspended in 100 µL of water, and 5 µL of each concentrated fraction was tested for *E. coli* inhibition. Aliquots of 50 µL of the active sample were derivatized using Fmoc and analyzed using the LC-MS general method. The active fraction identified via the inhibition assay was dried down again by rotary evaporation at 30 °C, resuspended in 80% acetonitrile/20% water, and purified on the Luna HILIC column (5 µm, 250 x 10 mm) at a flow rate of 5 mL/min. Metabolites in the active fraction were separated using mobile phase A (20 mM ammonium acetate) and mobile phase B (acetonitrile). Mobile phase was held at 90% B for 2 min followed by a gradient of 90–40% B over 18 min. Then 40% B was held for 5 min before the column was re-equilibrated to 90% B over 1 min. Fractions were collected every minute, and each 5 mL fraction was dried down overnight in the Genevac evaporator. Dried samples were resuspended in 100 µL of water, and the active fraction was identified using the *E. coli* inhibition assay.

Isolation of Ala-Cit$_{alkyne}$ from *Pss*

Pss WT cultures were centrifuged at 4,000 rcf, and the supernatant was extracted with an equal volume of 2:1 v/v methanol/chloroform. The aqueous extract from 1 L of culture was transferred to a round bottom flask and dried by rotary evaporation at 30 °C. The dry extract was resuspended in 40 mL of methanol, transferred to a 50 mL conical tube, and centrifuged at 4,000 rcf. The methanol supernatant was decanted into a fresh round bottom flask, dried by rotary evaporation at 30 °C, resuspended in 2% acetonitrile in water, and filtered using a 0.45 µm syringe filter for prep HPLC purification on the Luna C18 column (10 µm, 250 x 21.2 mm). The prep HPLC flow rate was 12 mL/min and the mobile phases included A (water) and B (acetonitrile). Mobile phase was held at 2% B for 10 min followed by a gradient of 2–98% B over 5 min, held at 98% B for 5 min, then re-equilibrated to 2% B over 3 min. Fractions were derivatized using Fmoc and analyzed using the general method for LC-MS analysis. Ala-Cit$_{alkyne}$ eluted within the first 10 min, and fractions containing Ala-Cit$_{alkyne}$ were pooled and dried by rotary evaporation at 25–30 °C. Dried prep HPLC fractions containing Ala-Cit$_{alkyne}$ were resuspended in a solution of 14% water/28% methanol/58% acetonitrile, and samples were centrifuged at 20,000 rcf for 10 min. The supernatant was purified on the Luna HILIC column (5 µm, 250 x 10 mm) at a flow rate of 5 mL/min using mobile phase A (20 mM ammonium acetate) and mobile phase B (acetonitrile) in two rounds of purification. For the first round, mobile phase was held at 90% B for 2 min followed by a gradient of 90–40% B over 18 min, held at 40% B for 5 min, and re-equilibrated to 90% B over 1 min. Elution peaks were identified by monitoring absorbance at 220 nm. Pooled fractions containing Ala-Cit$_{alkyne}$ were dried by rotary evaporation at 25–30 °C. Dried samples were resuspended in 14% water/28% methanol/58% acetonitrile and subjected to a second round of HILIC purification using a shallow gradient. Mobile phase was held at 90% B for 2 min, changed to a gradient of 90–60% B over 23 min followed by a gradient of 60–40% B over 5 min, and re-equilibrated to 90% B over 1 min. Purified fractions were pooled and dried via rotary evaporation at 25–30 °C before analysis by LC-MS and nuclear magnetic resonance (NMR) spectroscopy (Figures S5–8). The purified Ala-Cit$_{alkyne}$ was lyophilized, stored at −20 °C, and the sample was checked by NMR before use.

Ala-Cit_{alkyne} ($C_9H_{14}N_4O_4$). ^{1}H NMR (850 MHz, D_2O): δ 4.95 (t, J = 2.1 Hz, 1H), 4.13 (q, J = 7.1 Hz, 1H), 3.93 (d, J = 2.2 Hz, 2H), 1.59 (d, J = 7.1 Hz, 3H). HRMS (ESI): m/z $[M+H]^+$ calculated, 243.1088; observed, 243.1090.

Accession codes of Mbo enzymes

Accession numbers are MboA (ALE00418.1), MboB (ALE00417.1), MboC (ALE00416.1), MboD (ALE00415.1), MboE (ALE00414.1), and MboF (ALE00413.1).

Cloning of *mboA*, *mboC*, *mboD*, and *mboE*

Pss UMAF0158 genomic DNA was obtained as a template for cloning. *Pss* overnight culture was grown in 5 mL of KB at 30 °C with shaking at 225 rpm overnight. An aliquot of 1 mL overnight culture was centrifuged at 4,000 rcf, and the cell pellet was washed with phosphate-buffered saline (PBS) and resuspended in 200 µL water. Cells were boiled at 95 °C for 5 min and were frozen at −80 °C for 15 min. After thawing, the samples were centrifuged 20,000 rcf for 10 min, and the supernatant containing the genomic DNA was used as template.

Genes for *mboA*, *mboC*, *mboD*, and *mboE* were cloned directly from the *Pss* UMAF0158 genome (GenBank: CP005970.1) (Table S5). Each *mbo* gene was amplified by PCR using the Q5 high fidelity kit (New England Biolabs) from *Pss* genomic DNA (primers listed in Table S6). The amplified fragments were purified by gel extraction kit (Zymo Research). Fragments for *mboC*, *mboD*, and *mboE* were digested with BamHI and HindIII and ligated into pCDFDuet-His_6 (*mboC*), pACYCDuet-His_6 (*mboD*), or pRSFDuet-His_6 (*mboE*) using T4 DNA ligase. The *mboA* fragment was cloned into pLIC-His_6 vector as described.[4] Chemically competent *E. coli* NEB 5α cells (New England Biolabs) were transformed with each construct. Plasmids were extracted using the GeneJET Plasmid Miniprep Kit (Fisher Scientific) and confirmed by DNA sequencing.

Cloning of *mboB*

The *mboB* gene was codon-optimized for expression in *E. coli* and obtained in pET28a(+) from Twist Bioscience (Table S5). Codon-optimized *mboB* was amplified from pET28a(+)-*mboB*, and ligation independent cloning was used as described to generate the pLIC-His_6-MBP-*mboB* plasmid.[4] The resulting plasmid was confirmed by DNA sequencing.

Expression and purification of MboA, MBP-MboB, MboC, MboD, and MboE

E. coli BL21(DE3) were transformed with pLIC-His_6-*mboA*, pLIC-His_6-MBP-*mboB*, pCDFDuet-His_6-*mboC*, pACYCDuet-His_6-*mboD*, or pRSFDuet-His_6-*mboE*. Seed cultures were started in 5 mL LB supplemented with 100 µg/mL ampicillin (pLIC-His_6 vectors), 50 µg/mL spectinomycin (pCDFDuet-His_6-*mboC*), 34 µg/mL chloramphenicol (pACYCDuet-His_6-*mboD*), or 50 µg/mL kanamycin (pRSFDuet-His_6-*mboE*), and grown overnight at 37 °C. Overnight seed cultures were inoculated 1:200 into 1 L of fresh LB supplemented with respective antibiotics. Cultures were grown at 37 °C with shaking at 225 rpm until an optical density at 600 nm (OD_{600}) of 0.4–0.8 was reached. Protein expression was induced using either 0.5 mM IPTG (MBP-MboB expression) or 1 mM IPTG (MboA, C, D, E expression), and cells were grown at 16 °C with shaking at 225 rpm overnight. Cells were harvested at 4,000 rcf and cell pellets were resuspended in ~20 mL storage buffer per 1 L of culture (50 mM HEPES at pH 7.5, 150 mM NaCl, and 10% (v/v) glycerol). Cells were lysed by sonication on ice for 3 min (30% amplitude, 1 sec on, 1 sec off cycles) using the Sonic Dismembrator (Fisher Scientific Model 505). Cell lysate was clarified by centrifugation at

15,000 rcf for 45 min at 4 °C, and the supernatant was filtered with a 0.45 µm syringe filter. Mbo proteins were purified by nickel affinity chromatography on an AKTA Pure FPLC or on benchtop using a 5 mL HisTrap column (GE). The proteins were loaded onto the column equilibrated with wash buffer (50 mM HEPES at pH 7.5, 150 mM NaCl, 25 mM imidazole, and 10% (v/v) glycerol) and then the column was washed with 4 column volumes of wash buffer. Proteins were eluted with elution buffer (50 mM HEPES at pH 7.5, 150 mM NaCl, 250 mM imidazole and 10% (v/v) glycerol). Fractions were analyzed using sodium dodecyl sulfate-polyacrylamide (SDS-PAGE) gel electrophoresis, and fractions containing the protein of interest were pooled. Imidazole was removed and purified proteins were exchanged into storage buffer using a desalting column (GE PD-10). The purity of each protein was determined by SDS-PAGE (Figure S9). Purified proteins were flash frozen in cryotubes using liquid N_2 and stored at −80 ℃.

Bioinformatic analysis of MboA, MboB, and MboE

MboA (ALE00418.1) was aligned with FlcE (AAG06907.1), SznF (QBA82042.1), UndA (AAY93576.2), BesC (WP_014151496.1), CADD (O84616.1), AetD (WP_208344496.1), and RohS (WP_025211972.1).

MboE (ALE00414.1) was aligned with arginine deiminase (WP_004416214.1), dimethylarginine dimethylamino hydrolase (NP_249886.1), succinylarginine dihydrolase (NP_416259.1), and amidinotransferase SxtG (ACG63830.1).

Primary sequence alignment was performed using Clustal Omega, and the alignments were visualized in JalView.[5, 6] Amino acid sequences for MboA, MboB, or MboE were uploaded to the AlphaFold server for structural prediction.[7] MboA was modeled with two Fe^{3+} ions, and MboB was modeled with FAD and two Fe^{3+} ions. Output structures were visualized in PyMOL, exported as protein databank (PDB) files, and uploaded to the Dali server to search for structural homologs against the PDB.

Reconstitution of MboC and MboD *in vitro*

MboC and MboD reactions were prepared in 100 µL of 50 mM HEPES or PBS at pH 7.5 containing 1 mM of each of the 20 canonical amino acids, 5 mM ATP, and 10 mM $MgCl_2$. Reactions were initiated with the addition of 10 µM MboC or 10 µM MboD and incubated for 1 h at 25 °C. The negative control did not contain enzymes. Reactions were quenched using an equal volume of acetonitrile, derivatized using Fmoc, and analyzed using the general LC-MS method. A reference library was created to contain all possible masses of positively charged ([M+H]+) Fmoc-derivatized di-, tri-, and tetra-peptides. Amino acid combinations that resulted in the same *m/z* were combined into one entry. This library was used as query masses with MassHunter Quantitative Analysis for MS 10.0 software for data analysis. Potential peptide products in the reaction were identified from LC-MS spectra based on retention times from 5 min to 13 min and errors within 20 ppm of calculated *m/z* values in the library. Integrated peak areas of the potential peptide products were exported as a table and further filtered. Each peak in a reaction was compared with the same peak in the no enzyme control, and peaks were only considered if they exhibited more than 20-fold higher abundance in the reaction than the no enzyme control. Further, any peaks in the enzyme reactions with an integrated area below 10^5 were not considered. When multiple peptides exhibited the same *m/z* values, the peak areas for these *m/z* were divided by the total number of possible combinations. The peak area for each amino acid in an identified peptide

product was calculated and graphed. The sum of these peak areas for each amino acid from different peptide products was plotted in a pie chart (Figures S10, S11).

Synthesis of Leu-Ala-Arg and Ala-Arg

All peptide analogues were synthesized using standard Fmoc-based solid-phase peptide synthesis procedures on 2-chlorotrityl chloride resin (0.9 mmol/g) (Aaptec). Resin (150 mg) was first swelled in 2 mL anhydrous DCM for 20 min at room temperature (RT) and then drained. The resin was then regenerated using a 2 mL solution of 25% thionyl chloride in anhydrous DCM, and the reaction was mixed with constant agitation for 40 min at RT. The solution was then drained into a conical flask containing 1 M NaOH for neutralization, and the regenerated resin was washed five times with 1 mL anhydrous DCM, discarding each wash into the neutralization conical flask. Following regeneration, the C-terminal amino acid was loaded onto the 2-chlorotrityl chloride resin. A solution of 3 equivalents (equiv.) of the C-terminal amino acid (Fmoc-L-Arg(Pbf)-OH) was prepared in 2 mL anhydrous DCM, with minimal DMF added to promote complete dissolution of the amino acid. Subsequently, 9 equiv. of DIPEA was added, and the solution was mixed with the resin for 2 h at RT with constant agitation. The loading solution was discarded, and un-loaded sites of the resin were capped using a solution of 100 µL 100% methanol in 2 mL anhydrous DCM for 30 min at RT with constant agitation. After capping, the solution was discarded and the resin was washed twice with 2 mL anhydrous DCM, and twice with 2 mL DMF. The N-terminal Fmoc protecting group was removed by treating the resin with 2 mL of 20% piperidine in DMF for 7.5 min at RT with constant agitation. This process was repeated once more, for a total of 15 min deprotection time. The resin was then washed twice with 2 mL DMF. Coupling solutions were prepared using 4 equiv. of the desired amino acid, 4 equiv. of HATU, and 8 equiv. of DIPEA in 2.5 mL DMF. The coupling solution was added to the resin and reacted for approximately 1 h with constant agitation. The resin was washed twice with 2 mL DMF. The deprotection and coupling process was repeated until the desired peptide was made, and the final N-terminal Fmoc-protecting group was deprotected as described above.

Following deprotection of the N-terminal amino acid, the resin was washed three times with 2 mL DCM. The resin was then washed with hexane for 5 min, drained, and allowed to dry under air for 10 min. A 5 mL solution of 2.5% water and 2.5% TES in TFA was prepared in a 15 mL polypropylene centrifuge tube. Dried resin was transferred from the reaction vessel to the 15 mL tube containing the cleavage cocktail, and peptide cleavage was conducted for 3 h with constant agitation. The resin was filtered through a cotton plug in a polypropylene syringe and washed with a small amount of TFA. The filtrate was collected in a pre-weighed 15 mL glass scintillation vial, and the cleavage solution was dried by rotary evaporation. Peptide was resuspended in a minimal amount of 50% acetonitrile in water. A small aliquot was removed to verify successful synthesis and relative purity by LC-MS, and the peptide was flash frozen and lyophilized for a minimum of 24 h. Dried peptide was stored at −20 °C until purification.

The crude peptide was dissolved in 5% acetonitrile in water to a final concentration of 25 mg/mL and the resuspension was filtered through a 0.45 µm syringe filter. Crude peptide was purified using prep HPLC in 5 mL portions on a Luna C18 column (10 µm, 250 x 21.20 mm) and separated using mobile phase A (water, 0.1% TFA) and mobile phase B (acetonitrile, 0.1% TFA) and a gradient of 2% to 20 % B over 30 min at a flow rate of 12 mL/min. All absorbances in the range of 190−600 nm were recorded. Fractions containing the peptide of interest were determined by

LC-HRMS. Semi-purified peptides were then pooled, dried by rotary evaporation, and further purified using the Luna HILIC column (5 µm, 250 x 10 mm) and separated using mobile phase A (10 mM ammonium formate, pH 3.0) and mobile phase B (acetonitrile) and a gradient of 90% to 40% B over 18 min at a flow rate of 5 mL/min. These conditions were typically sufficient to yield peptides with at least 90–95% purity. Fractions containing pure peptide were pooled, flash frozen, and lyophilized for a minimum of 24 h. Dried purified peptide was stored at −20 °C. Following purification, peptide purity was assessed by LC-HRMS and NMR (Figures S15-16, S19-20).

Ala-Arg ($C_9H_{19}N_5O_3$): ^{1}H NMR (600 MHz, D_2O): δ 4.10 (dd, *J* = 8.1, 5.4 Hz, 1H), 4.04 (q, *J* = 7.1 Hz, 1H), 3.15 (t, *J* = 6.9 Hz, 2H), 1.79 (tt, *J* = 10.3, 6.5 Hz, 1H), 1.68 (dq, *J* = 14.3, 7.8 Hz, 1H), 1.57 (p, *J* = 7.4 Hz, 2H), 1.49 (d, *J* = 7.1 Hz, 3H). HRMS (ESI) *m/z* $[M+H]^+$ calculated, 246.1561; observed, 246.1516.

Leu-Ala-Arg ($C_{15}H_{30}N_6O_4$): ^{1}H NMR (600 MHz, D_2O) δ 4.37 (q, *J* = 7.1 Hz, 1H), 4.11 (dd, *J* = 8.1, 5.1 Hz, 1H), 3.96 (t, *J* = 7.3 Hz, 1H), 3.15 (td, *J* = 6.9, 1.8 Hz, 2H), 1.79 (dtt, *J* = 10.0, 7.1, 3.7 Hz, 1H), 1.70 (m, 1H), 1.63 (m, 2H), 1.56 (p, *J* = 7.3 Hz, 2H), 1.36 (d, *J* = 7.2 Hz, 3H), 0.91 (dd, *J* = 11.9, 6.3 Hz, 6H). HRMS (ESI) *m/z* $[M+H]^+$ calculated, 359.2401; observed, 359.2415.

MboA and MboC one-pot reaction

Reactions were prepared in 100 µL total volume containing 50 mM HEPES pH 7.5, 1 mM L-Arg, 1 mM L-Ala, 5 mM ATP, 10 mM $MgCl_2$, 100 µM $(NH_4)_2Fe(SO_4)_2 \cdot 6H_2O$, 1 mM ascorbate, 100 µM MboA, and 10 µM MboC. Reactions were incubated for 17 h at 25 °C with shaking at 300 rpm. All reactions were derivatized using Fmoc-Cl and analyzed using the general LC-MS method.

MboC reaction of Ala and D_7-Arg followed by MboA/MboB reaction

Reactions were prepared in 100 µL total volume containing 1 mM L-Arg or L-D_7-Arg, 1 mM L-Ala, 5 mM ATP, and 10 mM $MgCl_2$ in 50 mM HEPES pH 7.5. Reactions were initiated with the addition of 10 µM MboC and incubated for 12 h at 25 °C. These reactions were further incubated with 100 µM $(NH_4)_2Fe(SO_4)_2$, 1 mM ascorbate, 5 mM NADH, 10 µM MboA, and 1 µM MBP-MboB for 1.5 h at 25 °C with shaking at 300 rpm. All reactions were derivatized using Fmoc-Cl and analyzed using the general LC-MS method.

One-pot reaction containing MboA, MboB, MboC, and MboE

Reactions were prepared in 100 µL total volume containing 1 mM L-Arg, 1 mM L-Ala, 100 µM $(NH_4)_2Fe(SO_4)_2$, 1 mM ascorbate, 5 mM NADH, 5 mM ATP, 10 mM $MgCl_2$, 10 µM MboA, 1 µM MBP-MboB, 1 µM MboC, and 1 µM MboE in 50 mM HEPES pH 7.5. MboE was added after the reaction proceeded for 1.5 h, and reactions were incubated for 3 h total. For reactions containing synthetic Leu-Ala-Arg or Ala-Arg as substrates, 1 mM peptide substrate were first incubated with 100 µM $(NH_4)_2Fe(SO_4)_2$, 1 mM ascorbate, 5 mM NADH, 10 µM MboA, and 1 µM MBP-MboB for 1.5 h, and then MboE was added and the reaction was incubated for another 1.5 h. All reactions were Fmoc-derivatized and analyzed using the general LC-MS method.

Testing the substrate scope of MboA and MboB

Reactions were prepared in 100 µL total volume containing 50 mM HEPES pH 7.5, 1 mM L-Leu, 1 mM L-Ala, 1 mM of the variable amino acid (Xaa), 5 mM ATP, 10 mM $MgCl_2$, 10 µM MboC, and 10 µM MboD. The variable amino acid included L-canavanine, N^G, N^G-dimethyl-L-arginine, N^G-hydroxy-L-arginine, L-2,4-diaminobutyric acid, agmatine, L-citrulline, L-ornithine, L-arginine, D-arginine, L-homoarginine, N^G-methyl-L-arginine, and L-lysine. Reactions were incubated for 17 h at 25 ℃, then separated into two 50 µL aliquots. MboA and MBP-MboB were added to one aliquot and the resulting 60 µL reaction mixture contained 100 µM $(NH_4)_2Fe(SO_4)_2$, 1 mM ascorbate, 5 mM NADH, 10 µM MboA, and 1 µM MBP-MboB. The other aliquot was used for the control reaction that contained everything except for MboA and MBP-MboB. These reactions were incubated for 3 h at 25 ℃ with shaking at 300 rpm. All reactions were derivatized using Fmoc-Cl and analyzed using the general LC-MS method.

Phylogenetic analysis of the *Pseudomonas syringae* group and identification of *mboA-F* genes in *Pseudomonas*

The 16S rRNA sequences of all organisms in the *Pseudomonas syringae* group (taxon ID: 136849) were downloaded and aligned using MAFFT.[8] The alignment was then used to create a phylogenetic tree using the unweighted pair group method with arithmetic mean (UPGMA) on the MAFFT web server (https://mafft.cbrc.jp/alignment/server/phylogeny.html).[8] The tree was downloaded in Newick format and visualized on iTOL (https://itol.embl.de/).

Homologs of *mboA-F* genes were identified using a multi-gene BLAST. All *Pseudomonas* genomes (taxon ID: 286) available in NCBI (~74,000 genomes) were downloaded and BLAST was performed against these genomes using *mboA-F* as queries and an e-value cutoff of at least 150 for each gene. The organisms that had all six genes were then extracted from the BLAST results (Supporting File 2). The organisms that belong to the *Pseudomonas syringae* group (taxon ID: 136849) were mapped with their corresponding nodes in the phylogenetic tree and represented using a red circle (Figure S28).

Supplementary Tables

Table S1. Top comparative metabolomics hits of ions present in *Pss* WT and not Δ*mgo* with proposed structures. Ions around 8.6 min are likely in-source fragments or dimers of Fmoc-Ala-Cit$_{alkyne}$ or Fmoc-Cit$_{alkyne}$, respectively.

**Fold upregulation**	**Observed *m/z*$_{median}$**	**Difference between observed and calculated *m/z***	**Retention time (min)**	**Proposed species and calculated *m/z***
30982	465.1763	1.3 ppm	8.6 min	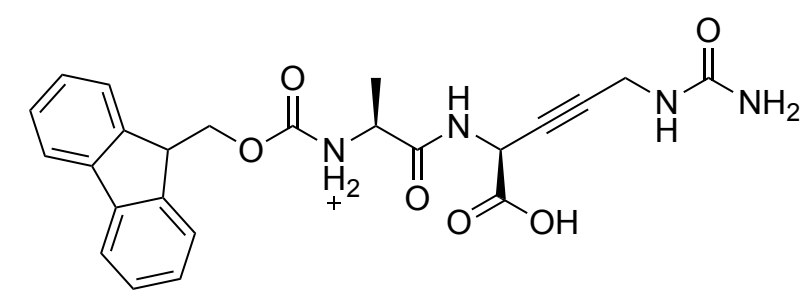<br>Chemical formula: $C_{24}H_{25}N_4O_6^+$<br>Exact mass: 465.1769<br>[M+H]$^+$ Fmoc-Ala-Cit$_{alkyne}$
4787	394.1392	1.3 ppm	8.6 min	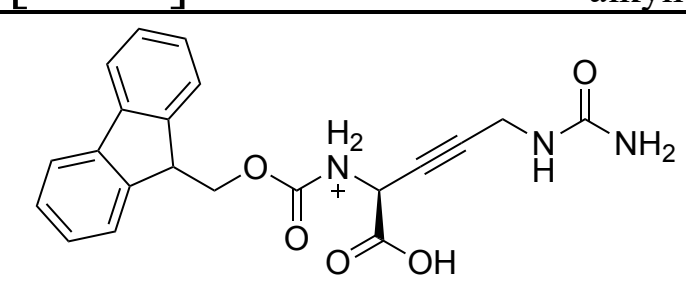<br>Chemical formula: $C_{21}H_{20}N_3O_5^+$<br>Exact mass: 394.1397<br>[M+H]$^+$ Fmoc-Cit$_{alkyne}$
2687	416.1211	1.4 ppm	8.6 min	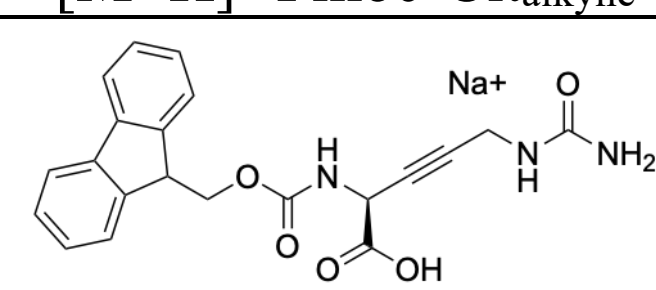<br>Chemical formula: $C_{21}H_{19}N_3NaO_5^+$<br>Exact mass: 416.1217<br>[M+Na]$^+$ Fmoc-Cit$_{alkyne}$
2405	787.2710	1.5 ppm	8.6 min	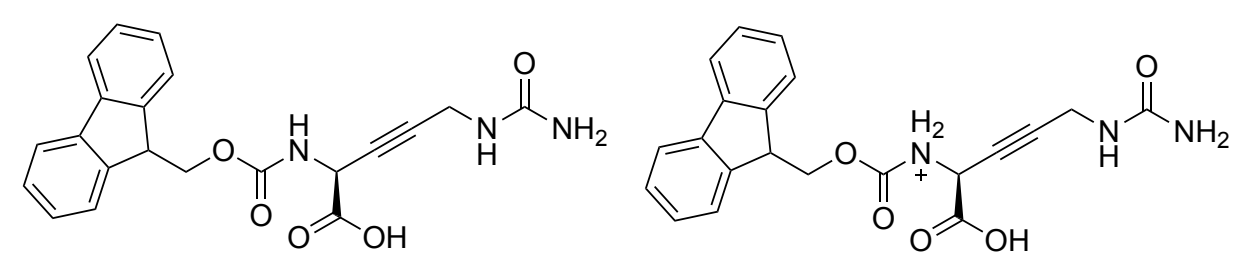<br>Chemical formula: $C_{42}H_{39}N_6O_{10}^+$<br>Exact mass: 787.2722<br>[2M+H]$^+$ Fmoc-Cit$_{alkyne}$
2163	929.3452	1.3 ppm	8.6 min	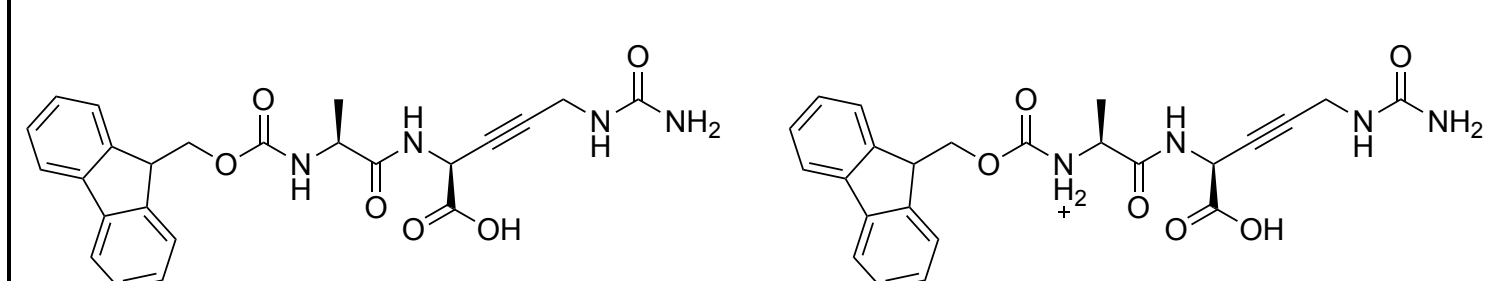<br>Chemical formula: $C_{48}H_{49}N_8O_{12}^+$<br>Exact mass: 929.3464<br>[2M+H]$^+$ Fmoc-Ala-Cit$_{alkyne}$

1701	422.1704	1.4 ppm	8.6 min	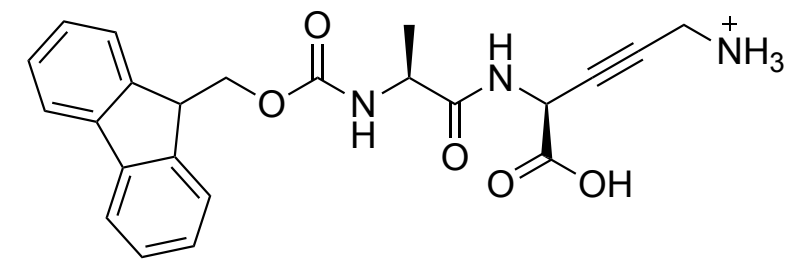<br>Chemical formula: $C_{23}H_{24}N_3O_5^+$<br>Exact mass: 422.1710<br>$[M+H]^+$ Ala-Cit$_{alkyne}$<br>In-source fragment
625	464.1925	0.6 ppm	6.7 min	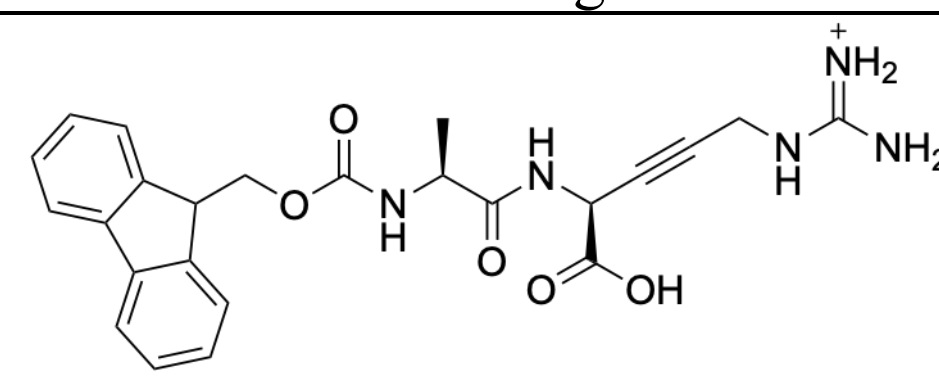<br>Chemical formula: $C_{24}H_{26}N_5O_5^+$<br>Exact mass: 464.1928<br>$[M+H]^+$ Fmoc-Ala-Arg$_{alkyne}$
128	393.1555	0.5 ppm	6.7 min	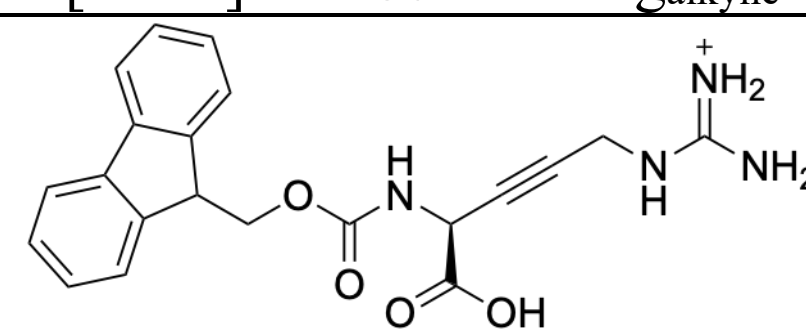<br>Chemical formula: $C_{21}H_{21}N_4O_4^+$<br>Exact mass: 393.1557<br>$[M+H]^+$ Fmoc-Arg$_{alkyne}$

Table S2. NMR shifts and 2D correlations of isolated Ala-Cit$_{alkyne}$. Arrows indicate the observed heteronuclear multiple bond correlation (HMBC) correlations.

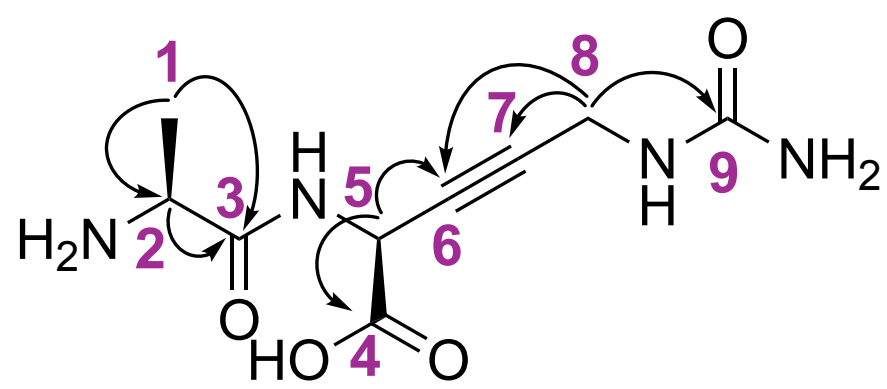

Position	δ$_H$ (ppm)	Mult. (*J* in Hz)	δ$_C$ (ppm)	HMBC (ppm)
1	3H, 1.59	d (7.1)	18.88	C2, 51.68<br>C3, 172.30
2	1H, 4.13	q (7.1)	51.68	C3, 172.30
3			172.30	
4			175.36	
5	1H, 4.95	t (2.1)	49.67	C4, 175.36<br>C6, 80.27
6			80.27	
7			83.06	
8	2H, 3.93	d (2.2)	32.29	C6, 80.27<br>C7, 83.06<br>C9, 163.80
9			163.80	

Table S3. NMR shifts of synthesized Ala-Arg.

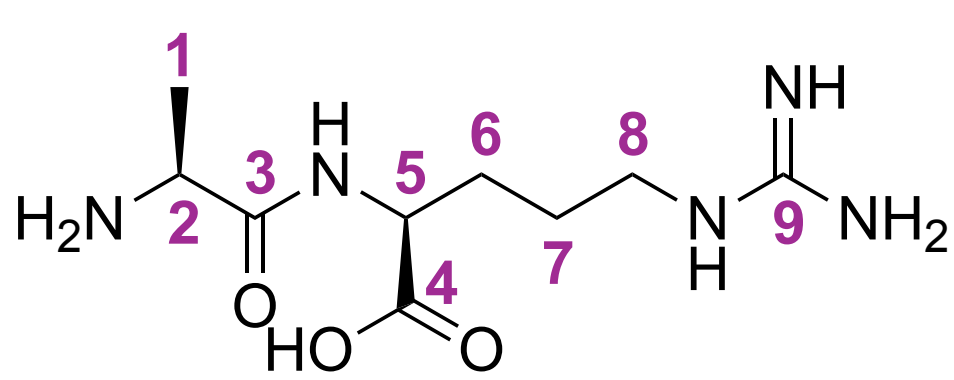

Position	δ$_H$ (ppm)	Mult. (*J* in Hz)	δ$_C$ (ppm)
1	1.49	d (7.1)	16.44
2	4.04	q (7.1)	48.92
3			170.17
4			178.20
5	4.10	dd (8.1, 5.4)	55.11
6a	1.68	dq (14.3, 7.8)	28.41
6b	1.79	tt (10.3, 6.5)	
7	1.57	p (7.4)	23.19
8	3.15	t (6.9)	40.56
9			156.67

Table S4. NMR shifts of synthesized Leu-Ala-Arg.

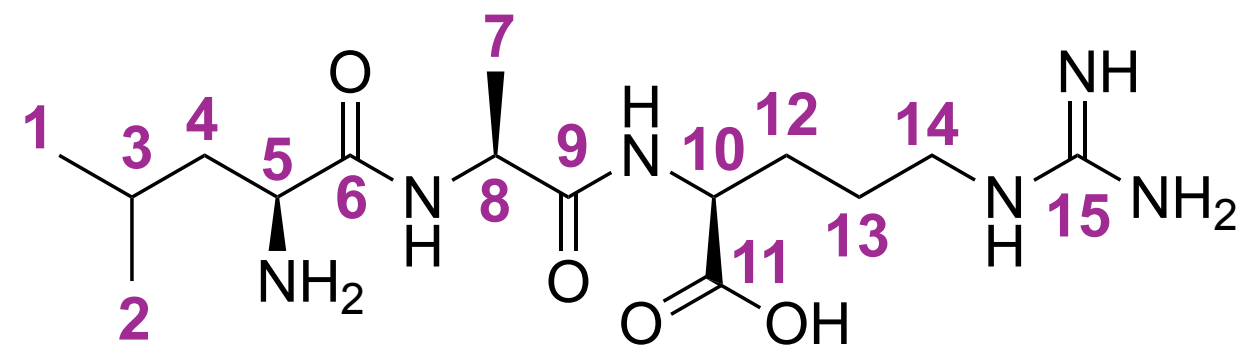

Position	δ_H (ppm)	Mult. (*J* in Hz)	δ_C (ppm)
1, 2	0.91	dd (11.9, 6.3)	21.60, 21.12
3	1.70	m	23.12
4	1.63	m	39.87
5	3.96	t (7.3)	51.60
6			170.07
7	1.36	d (7.2)	16.36
8	4.37	q (7.1)	49.67
9			173.42
10	4.11	dd (8.1, 5.1)	54.61
11			178.18
12a	1.70	m	28.82
12b	1.79	dtt (10.0, 7.1, 3.7)	
13	1.56	p (7.3)	23.77
14	3.15	td (6.9, 1.8)	40.62
15			156.63

Table S5. Gene sequences of *mboA*, *mboC*, *mboD*, *mboE*, and codon-optimized *mboB*.

mboA
ATGATATCCACCGCTGAGATGCTAAAGACGCTTGAAAGCACCGTTGACGCCTGCCTG
GAAGCAGAATTCAAAAAAGACACTGTCATGGGCGAGTTCCATAAAGGCGACTGGTTC
AATGAGTCTTATTACAAGCGTCATATCCTGGAGTGCGTGATCCGCATTCACATGAACA
ACGAACTGGACGCACGGGCTGTACAGGTTGCGGCGGTCGATAACATTTCCGCGGCCA
AACAATTGTCCTACTATTTGTATGATGAGTTCGGCCACGACGAAATGTTCGGTCAGGA
CCTCGCTACGTACGGCTATTCCCCTTCCGATATTCAGGCGCAGTTCGCCTTTCCGGAA
ACCTGGAAACTCATGGGTTACCTGAACTTCTGCGTCAGCAAGTTCGGGCCATTGTCCT
CGATCACCTGGGACTGGTTCCTCGAATACTACGGCGACAAGTACAGCTCGTTCATCAC
TCAGAAGGCCAGCGCCAGCATGGGCCAGCCAGCGGTCAGCGGAGCGGCTTCGCACG
TGGCTTTCGACGAAGCCGAGGACCACAGCGGCATGATGAACAACATGCTGGCGTCG
GTTATCAAGGACGAGAAGGATCTGCAAAAGGCTGTCGTTCATATCAAGGCATTCGTG
CCGATGGTCGGTGAGTACTTCCAGGCCCTGCGTGCAGCAACGCTTTAA

***mboB* (codon optimized for *E. coli*)**
ATGGCCATTTATTCTGTCCGCATTGGGGATCAAACGTATACAGCGGAATCCCAACATCC
ATTAACGGACGCTCTGCCACACGAGGCATTAGTGCGGGGCTGCCTGAAGGGTGTTTG
TCGTGTTTGTAAATGTACGCTGGTCTCTGGCAGAGTGTTGGAGCAAGGCAAGGTGGT
CGCGCTGAACGAGGCATTCCTGCCTTGTGTTTCCCGAGCCGAGACAGACATCGACAT

TCGGGTGGCTATTTCGACATTCCACAAAGCGCGTTTGAAGAGTAAGCGCATCCTCTCG
GGCCAGATTATGGAAGTTGTCTTGGAAGTGAAGAAAGTATTCTACAATGCGAAAAGC
GTTATTTCTTTAAAGTATCCGGAAGGAAGTGCTGTCCGTTCGTACAGTGTCGTTACAC
TGGGCGGTAAAGACTATGATTCACTCACGTGTCACGTAAAATTACGCGAAGGCGGGC
TCTTTTCAGGTTTGCTGGTCCAATGGCCCATCGGTGAGCCACTCGAATACTCCATCGC
CTCGCCGGCACTCCCCGTATACGAGCAAAGCTTGTCAAGATTAAACGTGGTTTCGGG
CGGTTCTGGCATGGGCGCAGCCTTAAGTCGCGCCCAAGAGTTAGTTGCTAAATATGGG
ATCTCCGAGGTAGCGATATATGCGGTAAACCGCGCTGGGTTGTCCGACTACCATGCTG
GTTGCATTGAAGTGTTCCGCAAAACCGTGGAGTGTGATGTGCAAGTAACGAATTTCC
CGTTCTCTGAGTGGACGCATCCCGATTTTGCTATCGGAGAGCATCTGATGCATGATACT
CTGACAATTGGTGTTGGCTCAGACGGTGTAATTGGTAAACTGAAGAATCTGCCGTTAT
GCGAGCTGGAGTCCTTCGGTTAA

mboC
ATGTCCATCGTTGTCATTGATCCTGTTTCATCCGGCATTAACTATATCAATGCGGCGCA
GCAACTGGGCGTCGATGTCTATGTGTTTTCCTGTGACGGTGGCGAGCAGGAGCTCAA
CGCTGAGTTGCGGGCCAAGGTGCGTCAGGTCATCAAAATCGACACCGGTGATTTCGA
TGCACAGCTAGAGAAAATCAATGAGTTGGGAAACATCCGCGCCGTTCTTCCCGGTGT
GGAGTACGCCGTGCCCATGGCCGCGCGCCTGGGGGCTGCGTCAGGCACAACGCATCT
GCACGACAGTGCAGTACAGAAAGTGCGCAACAAATTCAATTTCCGCAGTCGGCTGAC
TGACGCGGGGCTGAGCACTATCGGCTTTTTCCTTCTGGATGCCCACGCCCCGGTATCG
GTGCCGGAGGGTTTCAGCTTTCCGGCTGTGGTCAAGCCGATCGATATGGCGGGCAGC
ATCGGTGTGCGCAAGCTCTACAGCCATGAAGAGTTGGTCGAAGCGGTGGAGACATTC
CGTCAATCATTGCCCGACGACATCGGCTTTACTGCCAGTGGCCGGCTGATTGTCGAAG
CGTACATTCCCGGCACGGAATACAGTGTCGAAGGCATCGTGCGCAGCGATGGCAGCG
TCACCCTTGCGTCCATCACGGAAAAACTGCTCGGCTCGGAGCCGTACTTCGTCGAAG
TCGGTCACATTGTGGGCCAGGGCTATGACGAAGGGTTTCGCAAGGTGCTGACCGAAT
ACTCGCTGGCGGTGCTTGAGGCCATAAAGCTCAACGTCGGGCCGTTCCATCTGGAGC
TGCGTGTAACGCCACAAGGCCAGCCGGTGGCGGTTGAACTGGCGGCCCGCCTTCCA
GGCGACAATATCGTCGAGTTGATCAGGCGTGCCAGCGGCATTGACCTGGCCAAAGAG
ACGCTGTGTGAGTACCTGGACATCGACAGTCGCGCCGTGCCATGTGCGAACGGCGTC
AGTGCCATCGCCTTCATCCCCCGAGGCGACAAGAGCGAATTTACCGAACTGCAGGGG
CTCTCCGACATTTTCGACAGTCCGTATTACCTCTCGCATCAGGTGTATTACCGGTCCGG
GGATGCTCTGGGCTCCGATCAGGACTGGACCTCAAGAATTGGCTACGTCATGTTCGGC
GGCGCCGACGAGGCGCTTGTAAGAAGCCTCGTCGGGCGGGTACACACAGAGGTTGG
AATCGTATGA

mboD
ATGAAGCATGTCCTGATCATCAACCGCTATGACGACGAGCTTTCGGACTATCGCAACT
ACATCGATCACTCGCAGGTCGATGTCTCCTACATATCACTGGCAGGGCACTCGCGACT
GATTGATCCGCAGGTCAGCGCCAATGTGGTCGAAGTCAGCGTACTGGACGCCGATCT
GATACTTCAGGAAGCGCGGGCCATTCATCGTCGTCAGCCCGTCGATTTCGTGATCGCC
TTTTCGGAATACGATCTGGATGCCGCTGCGCTGGTGCGTACCGAATTCAACATACCCG
GTGCCAAGGTCGCCGATAACCTGCTGTGCCGTAACAAGGCGAGCATGAAGGAAGCCT
TGACCGGTTCTTCGGTTCGTTATCCTCAGTACCGGAATGTAGCGTCGCGCAGCGGCGT
CGAAGCCTTCTGTCGCGAGCACGGCTTTCCGGTCATCCTGAAACCCCAGGTGGGCGC

CGCCTCGGACGGCGTGGTGAAGATCGAACGCCTGGAAGACATACCCGACCTGCCGG
ATTTCAATGGCTACGAAGTCGAAGAATATATAGAAGGCGATATTTATCATGTCGACGCG
ATACTGGCGGCCGACAGCATGCCGTACTTCAAGGTGTCCAAGTACCTGAACACCTGC
CTGGACTTTCGCAACGGTCTGCCGCTCGGCTCGGTCACAGTCGATGACCGGGCATTC
ATCGCGCGGGTTCGCGCGTTCACCGAAGAAGTCTGCCTGCGTCTCAACCTGCACAAC
CAGGCCATTCACCTGGAAATCATCGAGCGTCGTGGCGAGCTGGTGTTTCTGGAAATC
GGCGGTCGCGTCGGTGGCGGCGAGATTCCGTTTGTCGCGTTGCGCAGCGAGGGGATC
GACCTTTATGAGCTCTGGACCCGCGCCGCGCTCGAAGTGGCTATCGCTCCGCTAGACA
CCCGAATCACCGGTTTTCTGATGATGCCCAATCCGTTCCCCGGCGGGTTCACCTTCGA
TCCGCAGATGGAGCTCTCCCATCCGCTGCTGACGTATAGCAGCGTGCAGTCTCAGGG
CGATTCAAGTGCGTTCAGCTACGAGGATATCCCCGCCAGACTGCACTTCGCCGCCGA
CAGCCAGGCTGCGATAGAGCAGGCCATTGTGCAGTGCATGAATATTTTGCAGACGTCT
ATTCGCGCAACCTGA

mboE
ATGAAAGTGCTCCTGCAGCTTCCCTCTGTCGTGCAGCCTGTTTCCATGTCGGTATTCAT
GGAATCGCAGCAGCCGTATTCGCTTCGGCAGGCAGATGCCGATGTCATGGCTATGGCC
AGTTCGTTCAATGCAGCGGGTTTCCCGACCGTCTTCCTGTCCGATGCGCTGTTCAGCG
AGGACGGTCCGCATGTCGCCATCGAGGATATGCGGCGGTTGGCAAGGCATGCATTGC
GCTTTGCCGACAACGTCGAACACGGGCGCCAGCAGCTTGCTCACCAGCAATTGTTCC
GCTGCAGTCGGCAGGAGTTGGCCGAAATCATCATCAACCAGCCTGAGCTGCGGCTGC
ATGACGATGACGAGTTGGGCCGGATCAGTCCGGACGCTACCTACGAGAGCTACCGGA
TTCAACCGCTGTACGGACTTCTGTTTCCGCGTGACCACTTCATGAACGTAGGTGGACA
GCCGGTTTTCGGGCGTCTGAAGCGTCAGGACAGGGCGCGTGAGGTTGATGTGGTGA
AGCAGGTGGTCGCTGCACATGGTCACGTTCCCGTCGAACTCGATCAGGTGCTCGAGG
GCGGCGACTATCTGGAAAATGAACGCGTATGCGTGATCAACACCGGCTTTCGTACCG
AGCTCCCGGTGCTGGAGTTCTTGCTGGCTTCAAACCTGTTCAAGGGGGAAGTTGTGC
TGGCGGTGCAGGACGTATGGCGCAATCCGGAACAGTTTCACCTGGATCACTACGCCT
GCCTGCTGCGCGACGCCCTGCTGGTCGACGCGAAAAGAGCGGACGCAGCCGACCGA
TCCCTGGTCAGCGTTTATCGGCGCGTGTCATCGGGCTGGCAACTGACTGACAAGGAC
CTGACGCTTCGCCAGGCAGCACAGGTTGCGGGCATCGGGCTGATACCGCTGGACGAA
GAAATGATGGCTGCATGGTGCGCAAATGCATTGTCATTGGGCAACCATGTCTGGATCG
ATCAGAACGCACCGGTGCGCCTGCTTGATCAGTTGGCTGAGCATGACTGTCATGTTCA
TCTGATCGCCTGTACAGAGCATCAAAAGCAGTTCGGCGGCATTCATTGCGCCACTCAA
CTCCTATAA

Table S6. List of primers used in this study.

Primer description	Sequence
mboA fwd	TACTTCCAATCCAATATGATATCCACCGCTGAGATG
mboA rev	TTATCCACTTCCAATTTAAAGCGTTGCTGCACGCAG
mboB fwd	TACTTCCAATCCAATATGGCCATTTATTCTGTCCG
mboB rev	TTATCCACTTCCAATTTAACCGAAGGACTCCAGCT
mboC fwd	GACAACAGGGATCCGATGTCCATCGTTGTCATTGATCCT
mboC rev	GACAACAGAAGCTTTCATACGATTCCAACCTCTGTGTG
mboD fwd	GACAACAGGGATCCGATGAAGCATGTCCTGATCATCAACCG
mboD rev	GACAACAG AAGCTTTCAGGTTGCGCGAATAGACGTCTG
mboE fwd	GACAACAGGGATCCGATGAAAGTGCTCCTGCAGCTT
mboE rev	GACAACAGAAGCTTTTATAGGAGTTGAGTGGCGCAATG

Supplementary Figures:

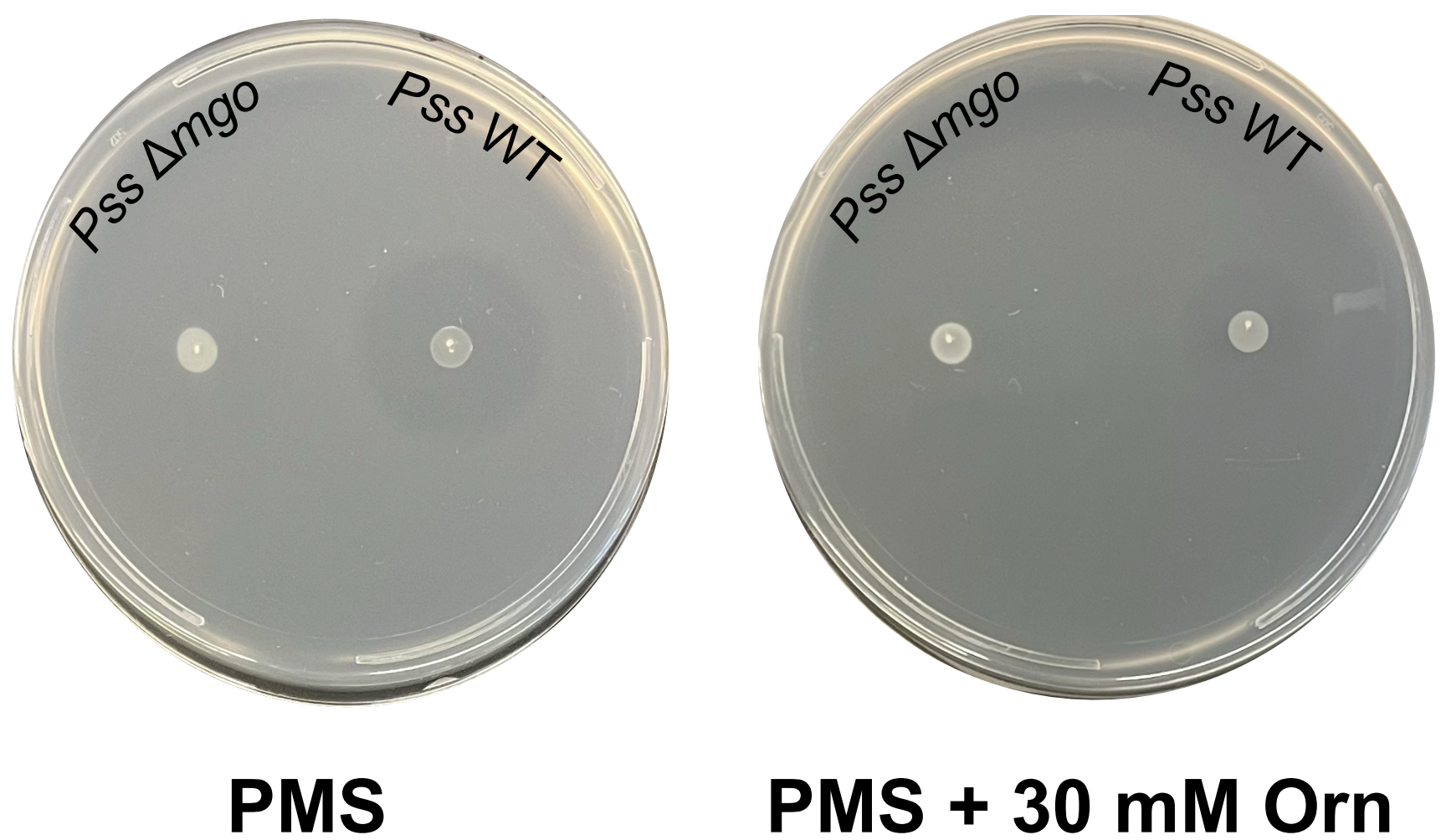

Figure S1. *E. coli* MG1655 grown on agar plates was inhibited by cultures of *Pss* WT but not Δ*mgo*. Inhibition was mostly reversed by ornithine (Orn). Inhibition of MG1655 is like that of BL21 (main text Figure 1B). In each plate, *Pss* Δ*mgo* is plated on the left, while *Pss* WT is plated on the right.

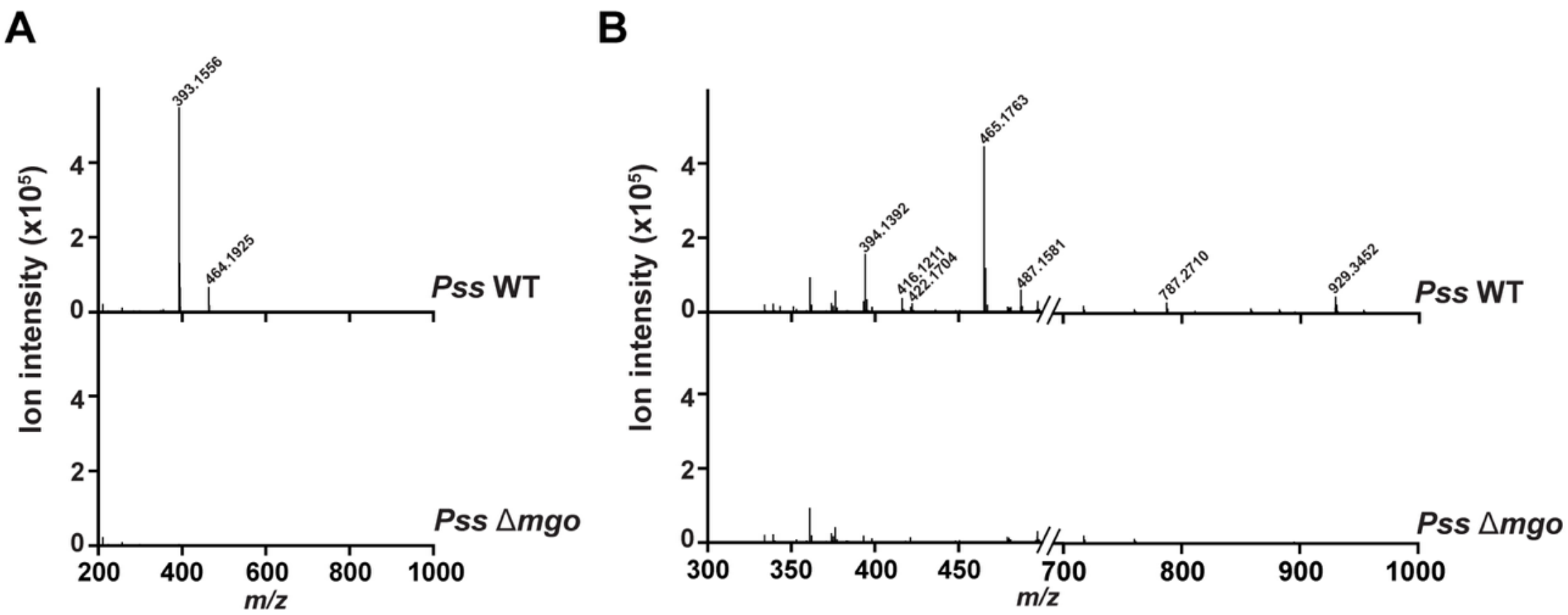

Figure S2. Multiple ions were identified in *Pss* WT but not the Δ*mgo* control. Mass spectra of the fraction eluted at the retention time of (A) 6.7 min and (B) 8.6 min in the *Pss* WT (top panels) and Δ*mgo* (bottom panels). Proposed structures of these ions are listed in Table S1.

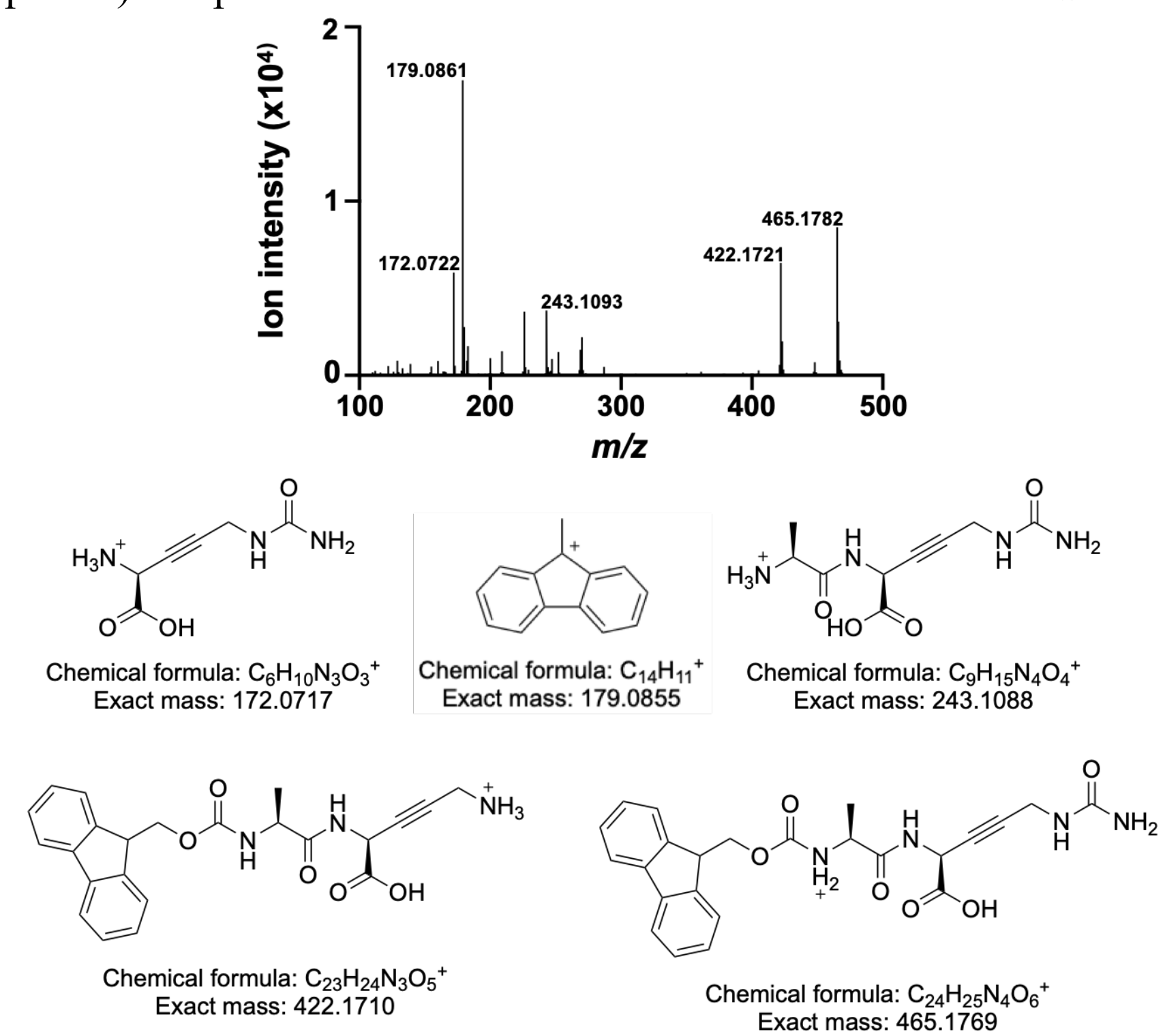

Figure S3. Tandem mass spectrum of Fmoc-Ala-Cit$_{alkyne}$ fragmented at 10 eV collision energy. Parent ion: Fmoc-Ala-Cit$_{alkyne}$ (calculated *m/z* 465.1769 $[M+H]^+$).

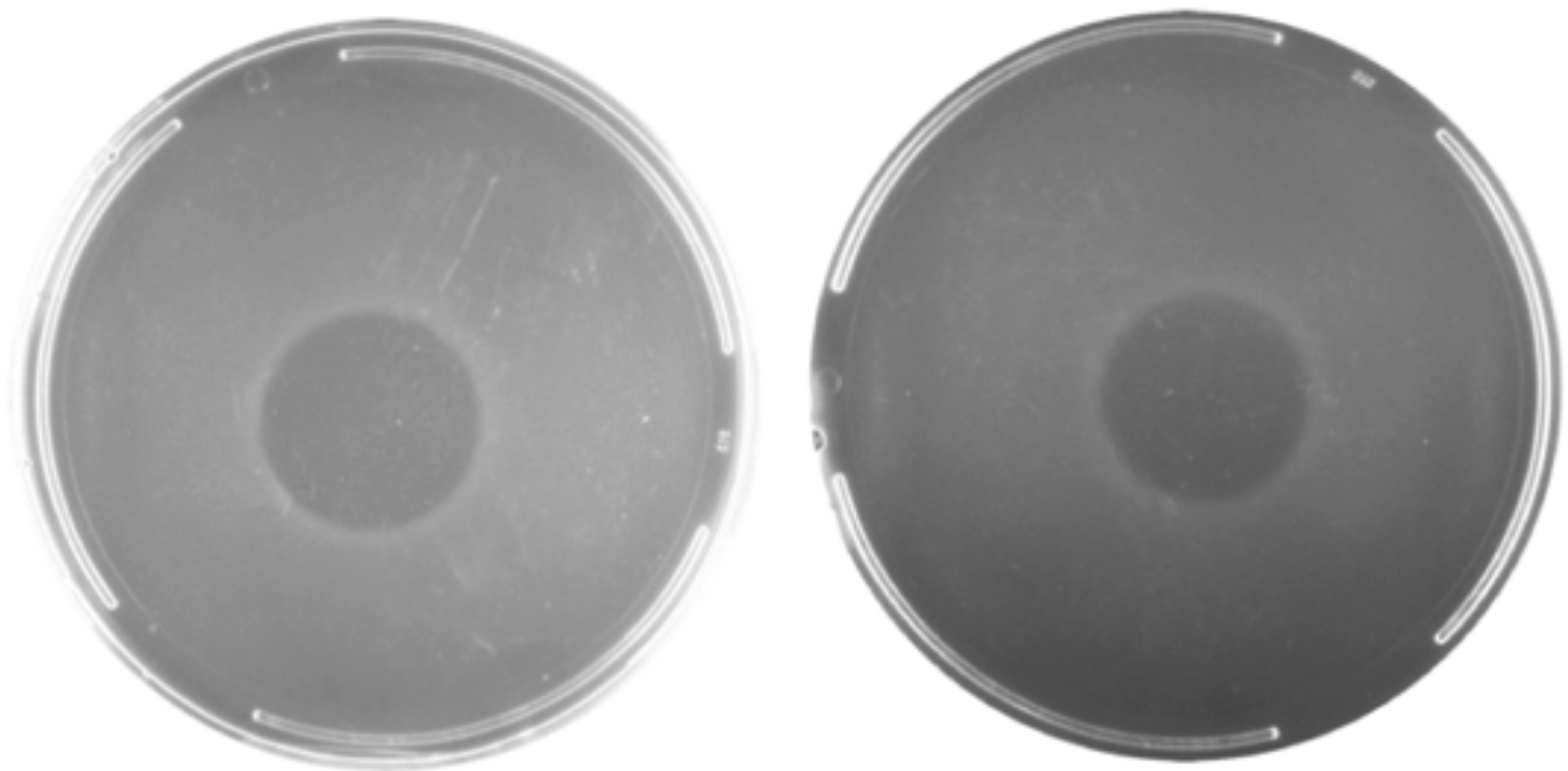

Figure S4. Inhibition of *E. coli* BL21 by isolated mangotoxin (Ala-Cit$_{alkyne}$). *E. coli* was grown in the PMS plate. A high concentration of mangotoxin (400 µM, 5 µL) was spotted on the plates. Zone of inhibition was observed (left) and was slightly reversed when ornithine was supplemented (right).

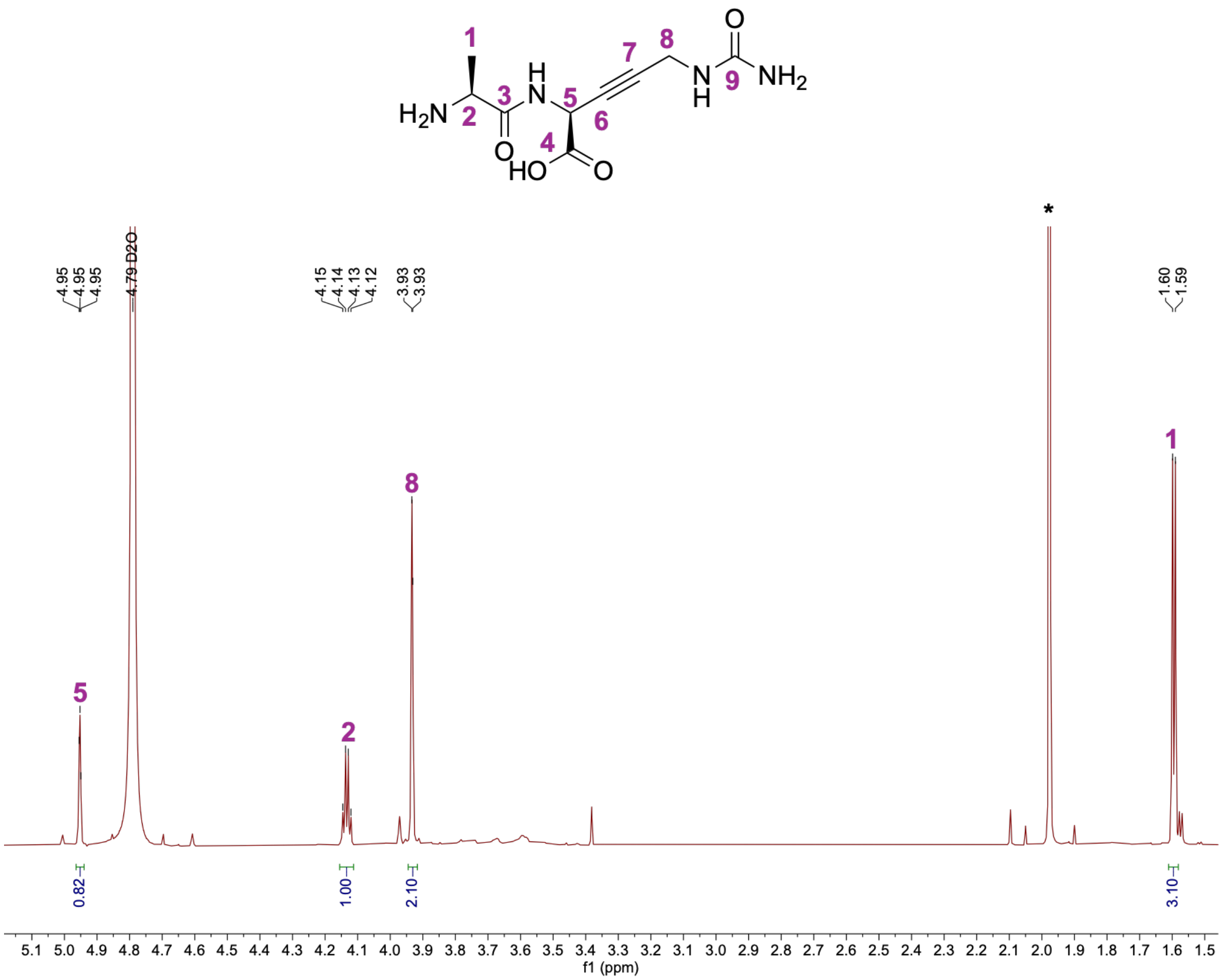

Figure S5. ^{1}H NMR spectrum (850 MHz) of Ala-Cit$_{alkyne}$ isolated from *Pss* UMAF0158 in D_2O. Asterisk (*) indicates acetate.

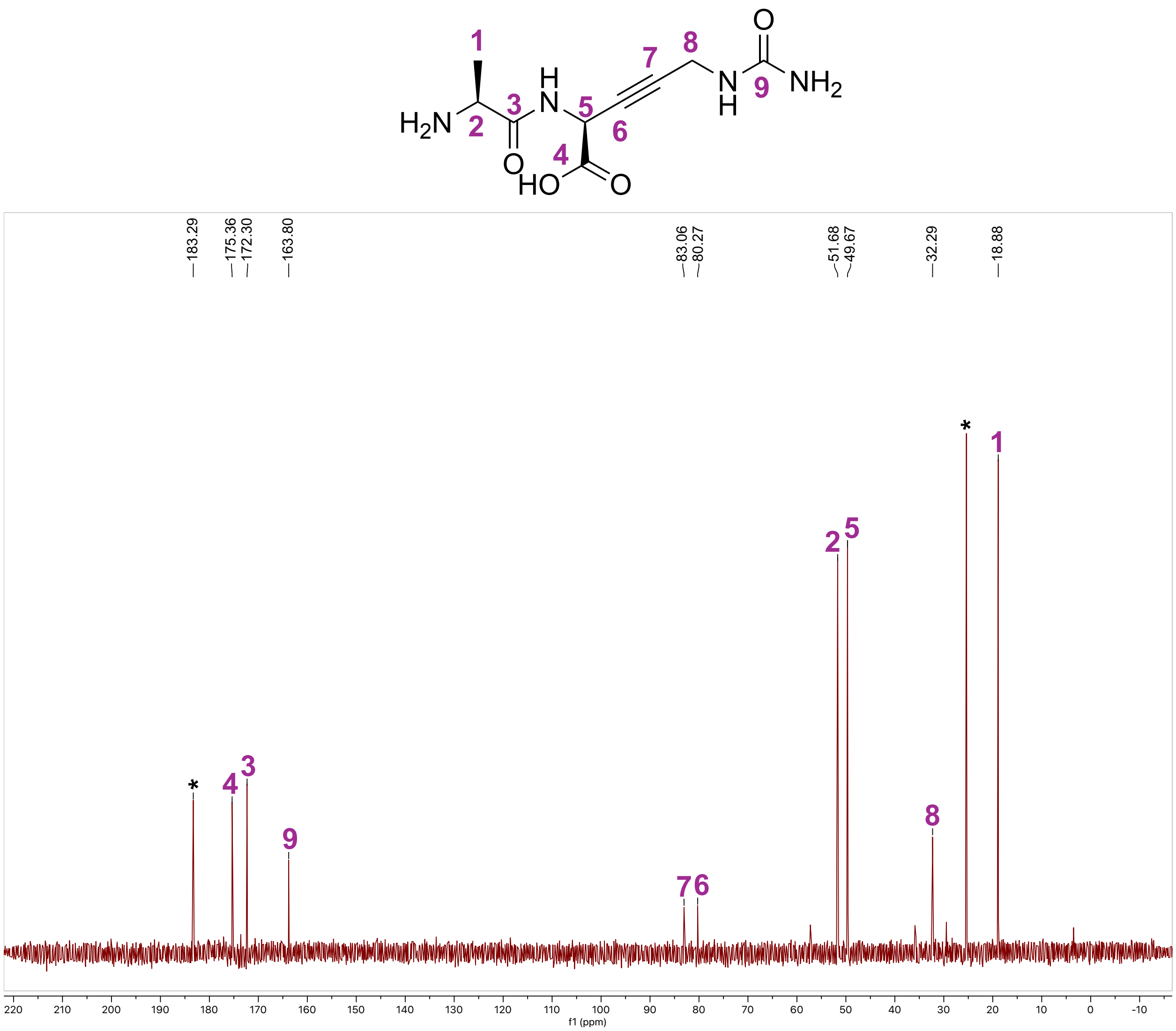

Figure S6. ^{13}C NMR spectrum (850 MHz) of Ala-Cit$_{alkyne}$ isolated from *Pss* UMAF0158 in D_2O. Asterisk (*) indicates acetate.

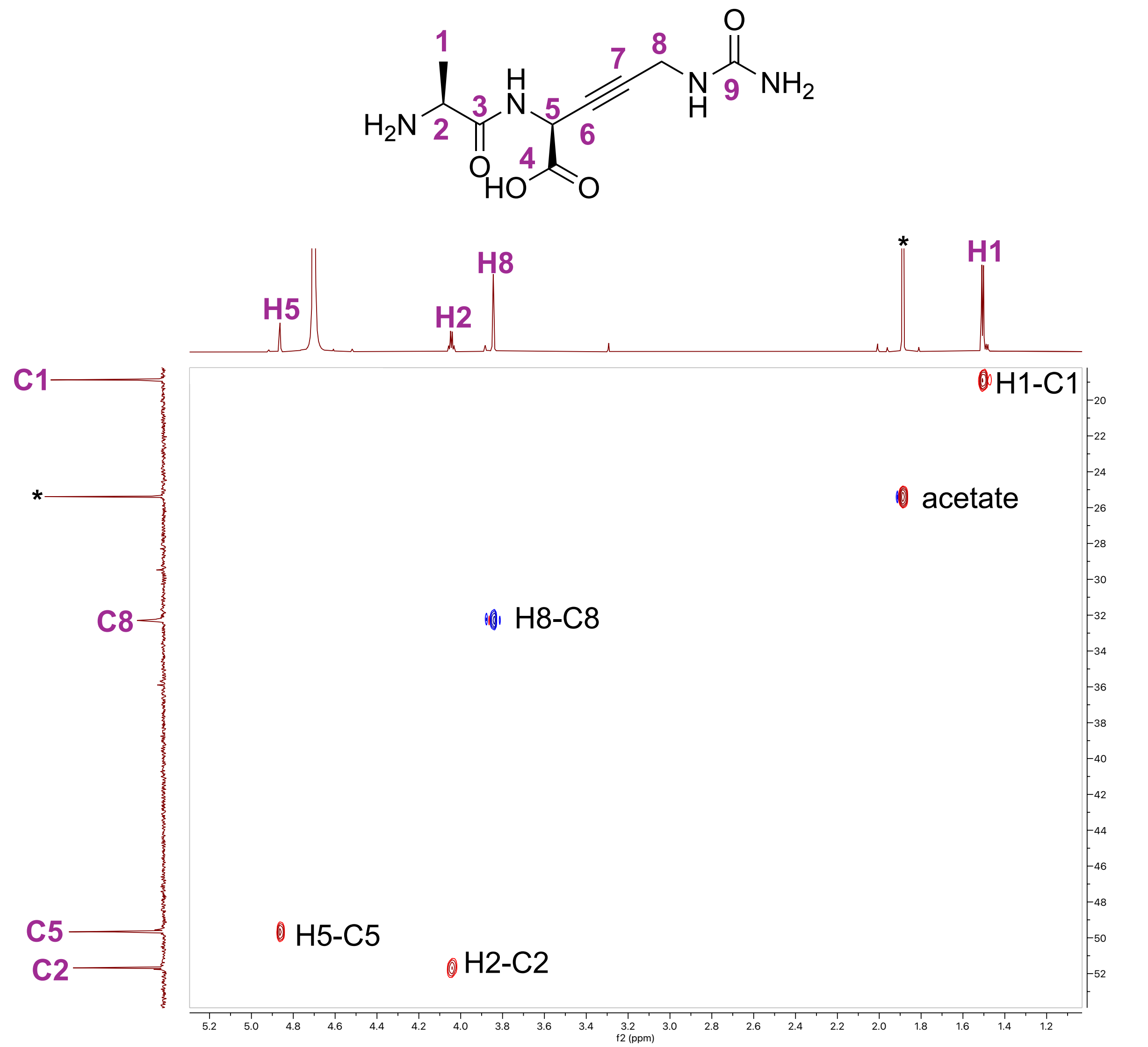

Figure S7. ^{1}H, ^{13}C-HSQC NMR spectrum (850 MHz) of Ala-Cit$_{alkyne}$ isolated from *Pss* UMAF0158 in D_2O. Asterisk (*) indicates acetate.

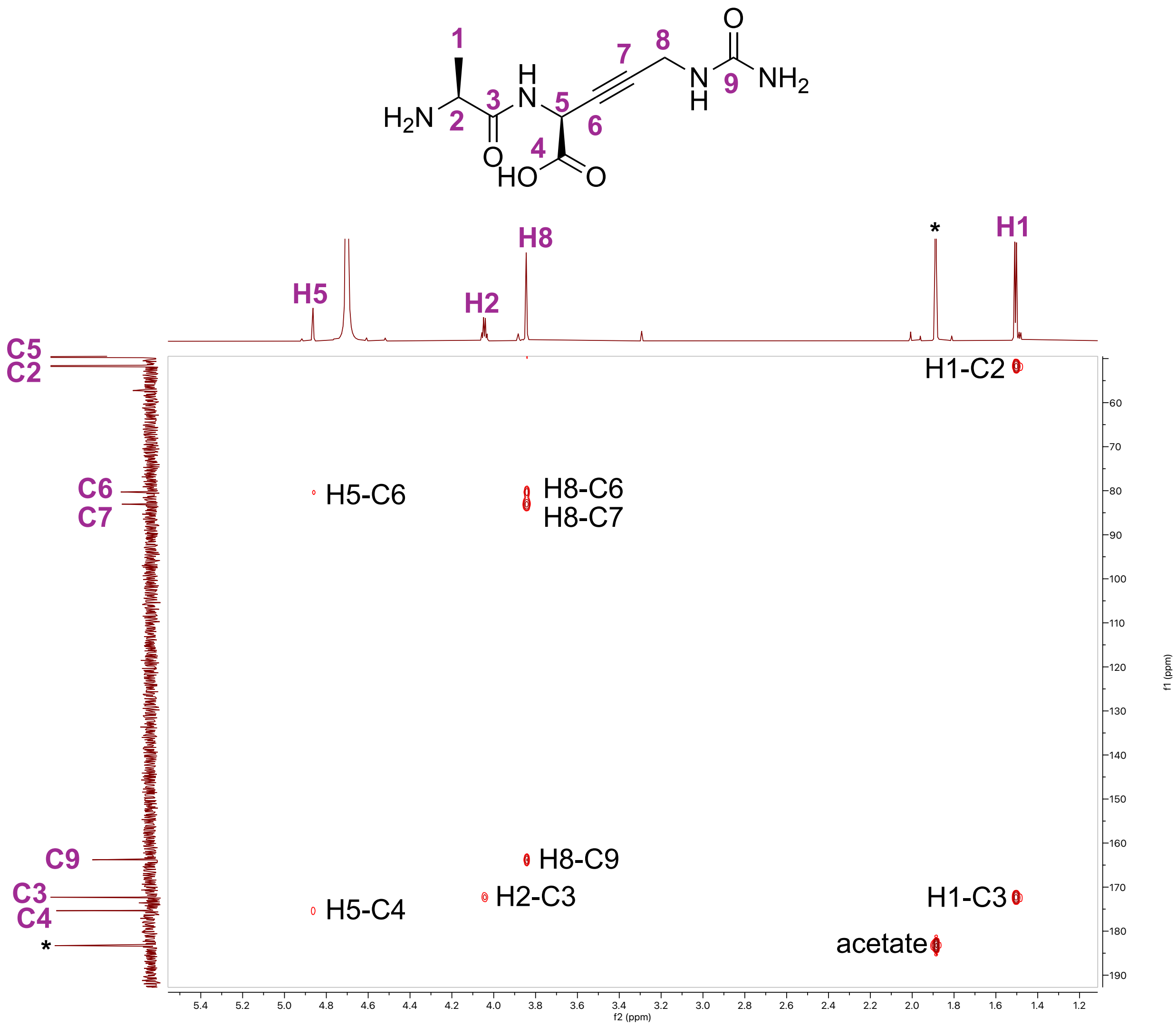

Figure S8. ^{1}H, ^{13}C-HMBC NMR spectrum (850 MHz) of Ala-Cit$_{alkyne}$ isolated from *Pss* UMAF0158 in D_2O. Asterisk (*) indicates acetate.

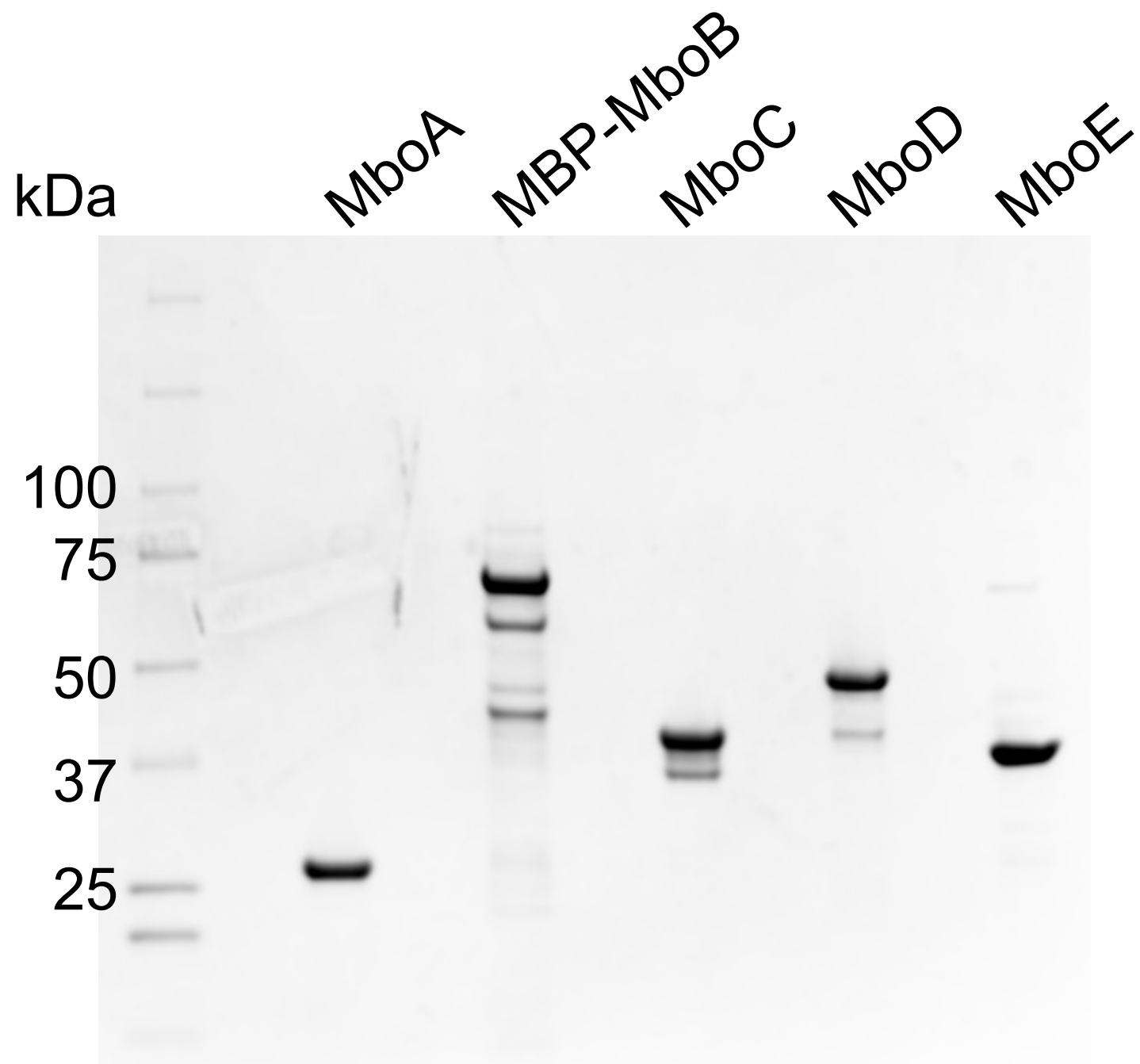

Figure S9. SDS-PAGE gel of purified MboA, MBP-MboB, MboC, MboD, and MboE at 5 µM. Expected sizes are: MboA, 25 kDa; MBP-MboB, 74 kDa; MboC, 44 kDa; MboD, 43 kDa; MboE, 41 kDa.

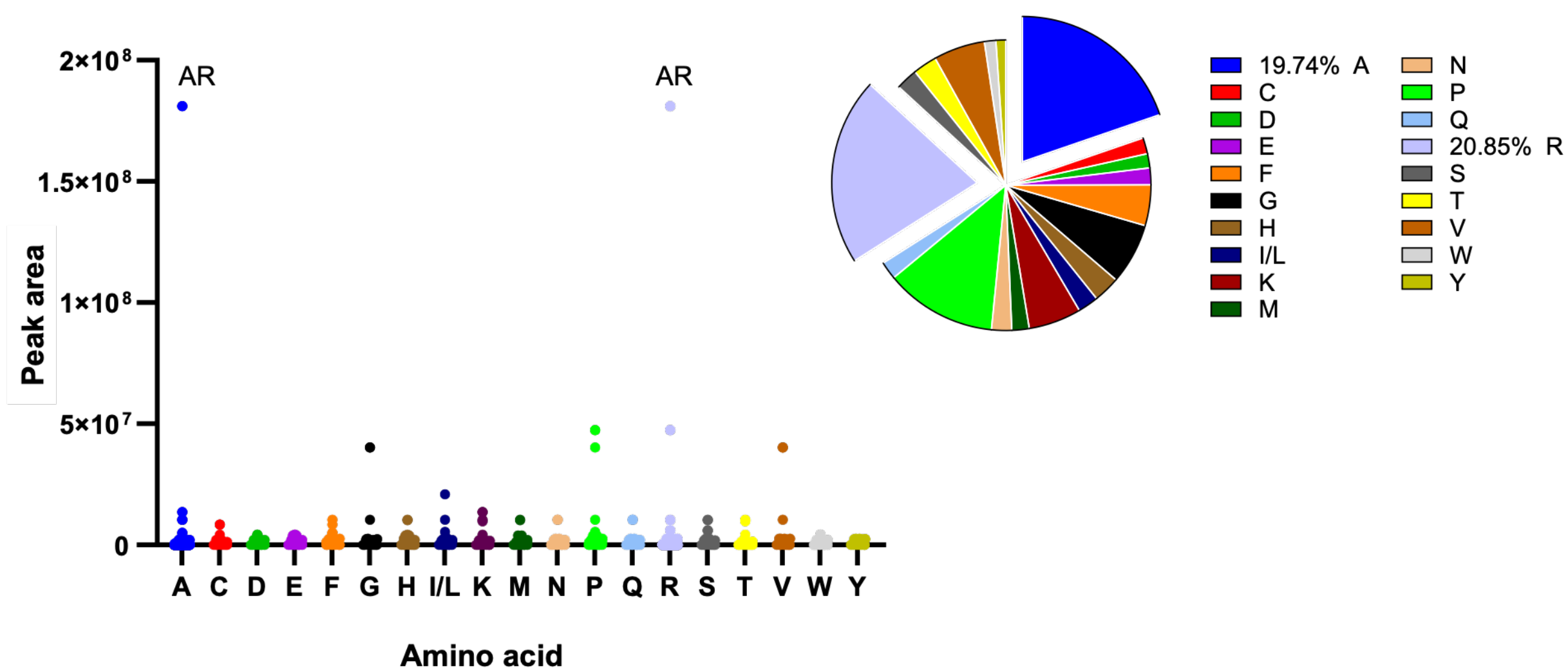

Figure S10. MboC preferentially catalyzes the formation of the alanine and arginine-containing dipeptide. Each amino acid is shown as the single letter amino acid code. The alanine and arginine dipeptide had the highest peak area. The pie chart shows percent incorporation for each amino acid in dipeptide products. Arginine and alanine were incorporated at the highest percentage.

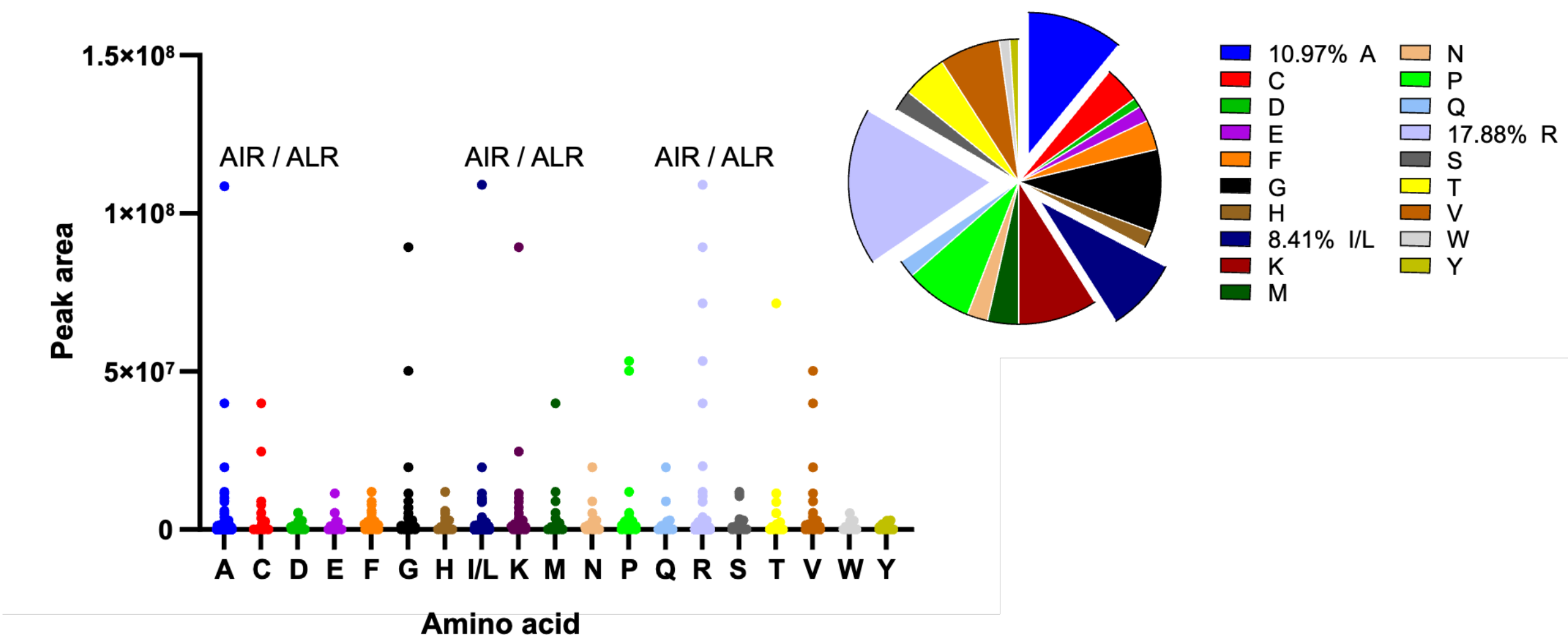

Figure S11. MboC and MboD preferentially catalyze the formation of a tripeptide consisting of alanine, arginine, and leucine or isoleucine. The pie chart shows the percent incorporation of amino acids in the tripeptide product. Arginine, alanine, and leucine or isoleucine were incorporated at the highest percentage.

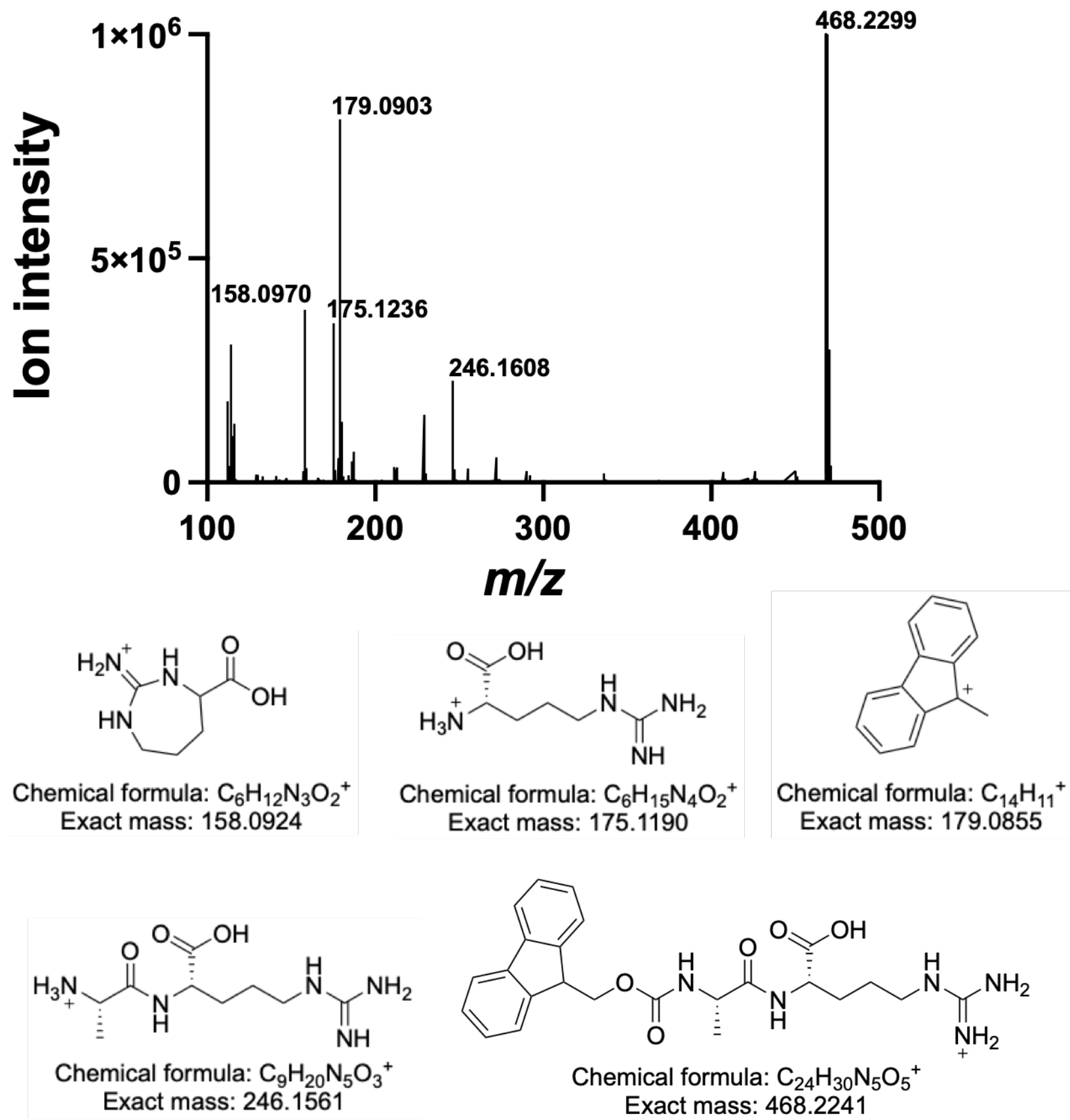

Figure S12. Tandem mass spectrum of Fmoc-Ala-Arg fragmented at 10 eV collision energy. Parent ion: Fmoc-Ala-Arg (calculated *m/z* 468.2241 $[M+H]^+$).

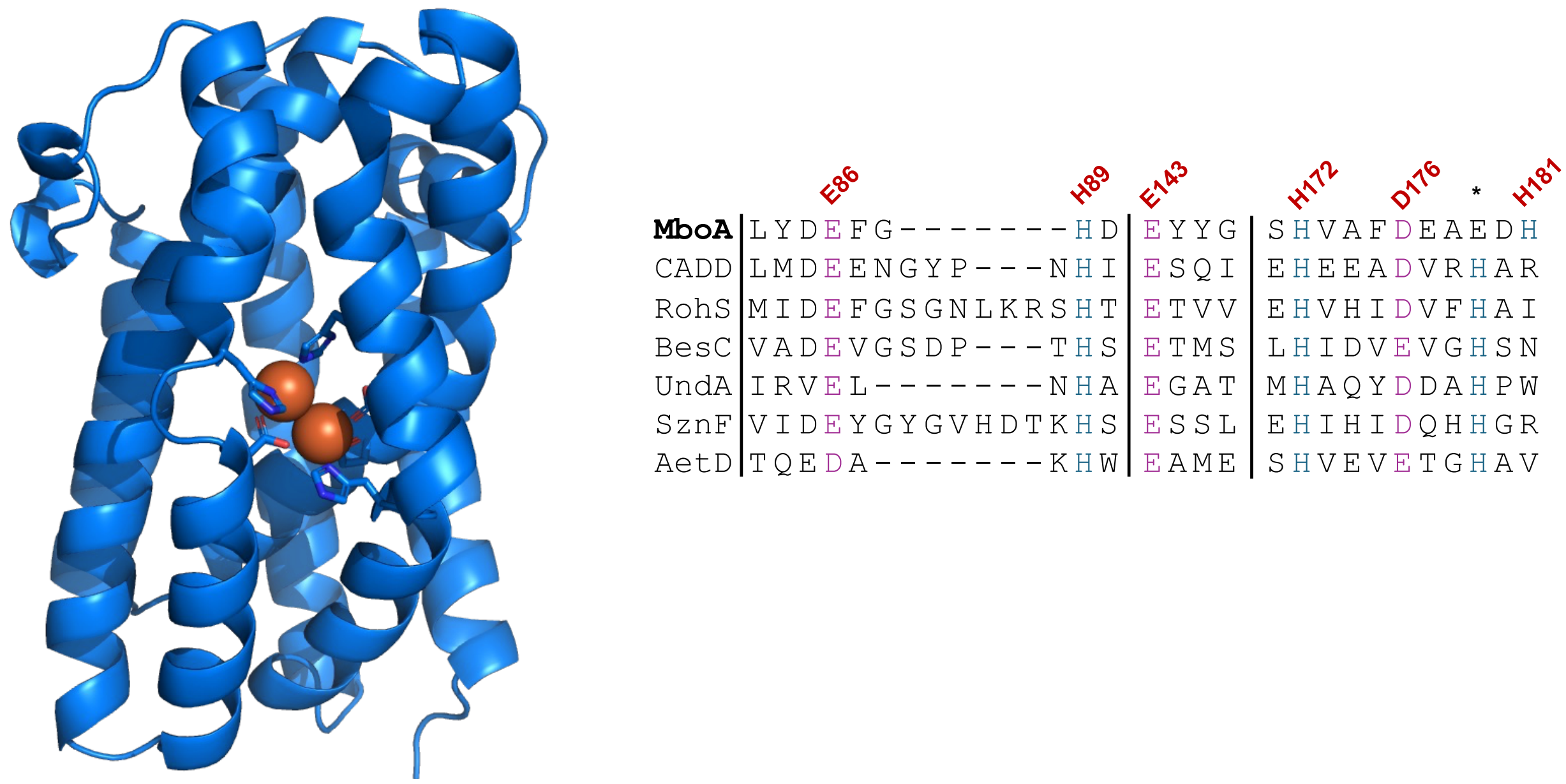

Figure S13. AlphaFold 3 model of MboA complexed with two Fe^{3+} (orange, left). Alignment of the predicted MboA dinuclear metal-binding motif with those of the canonical motif in characterized HDO enzymes (right). Putative MboA iron-binding residues are labeled in red. The asterisk on E179 indicates that H181 instead of E179 was predicted to bind iron, which is consistent with the recent reported structure of MboA.[9]

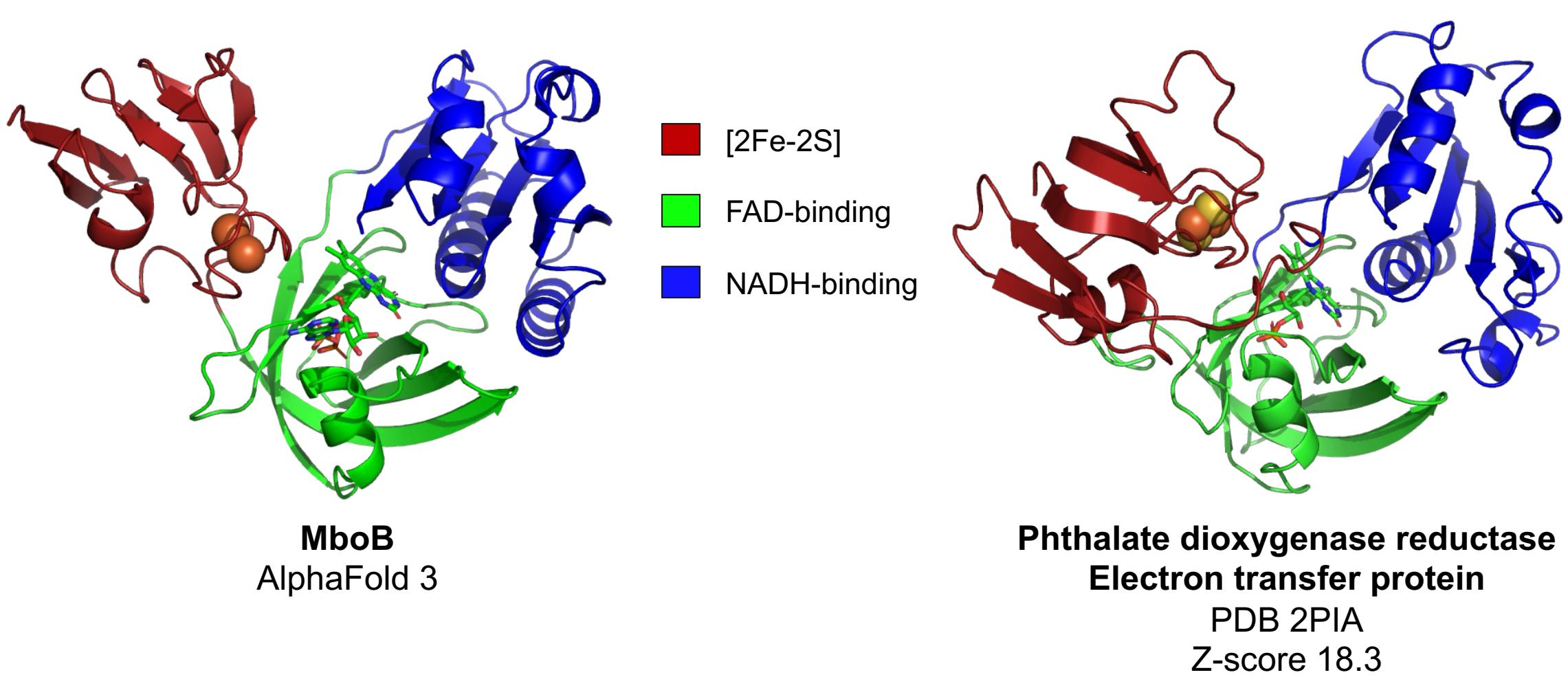

Figure S14. AlphaFold 3 model of MboB complexed with Fe^{3+} and flavin adenine dinucleotide (FAD) (left). The top hit from Dali search is phthalate dioxygenase reductase (right).

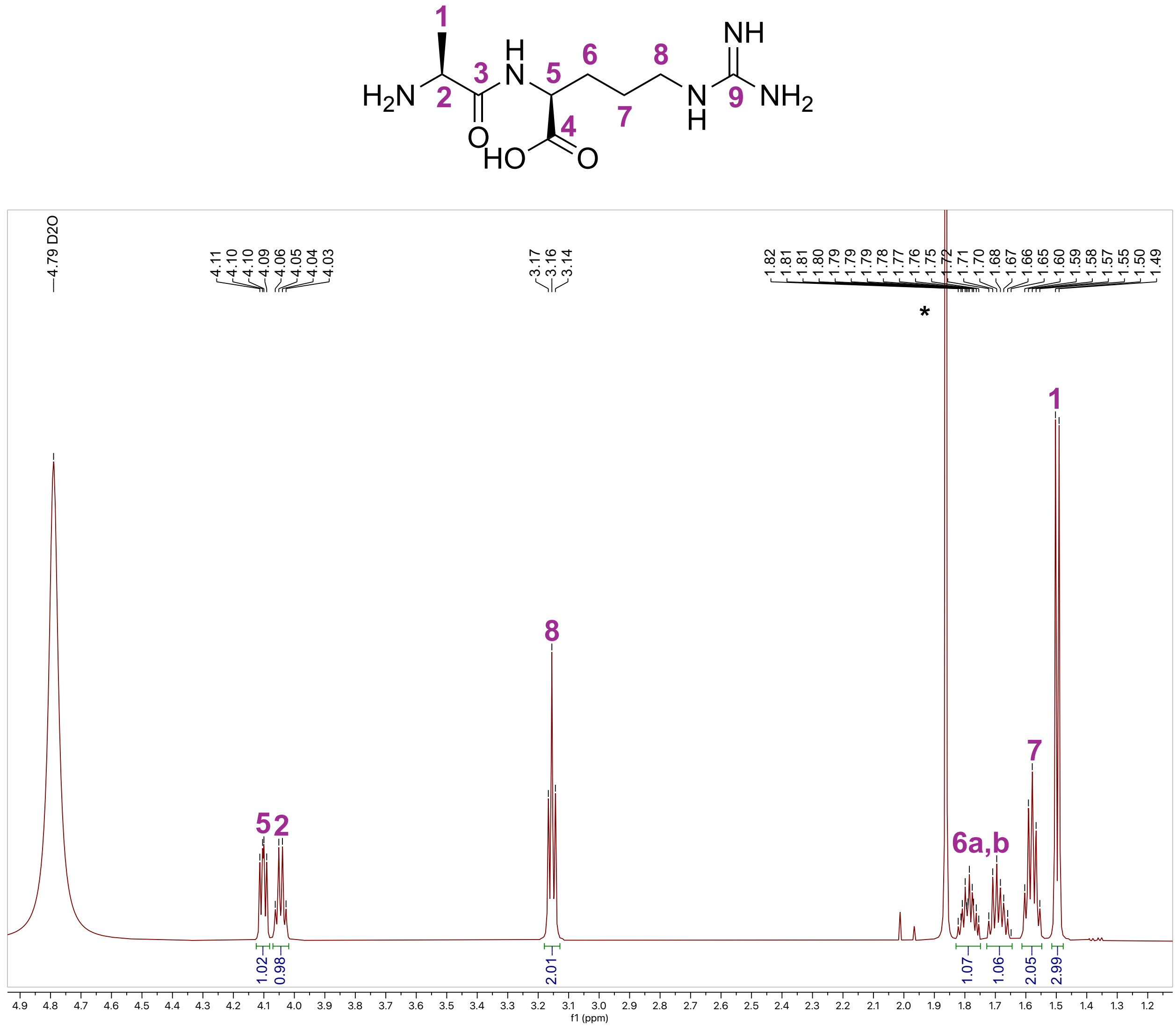

Figure S15. ^{1}H NMR spectrum (600 MHz) of Ala-Arg in D_2O. Asterisk (*) indicates acetate.

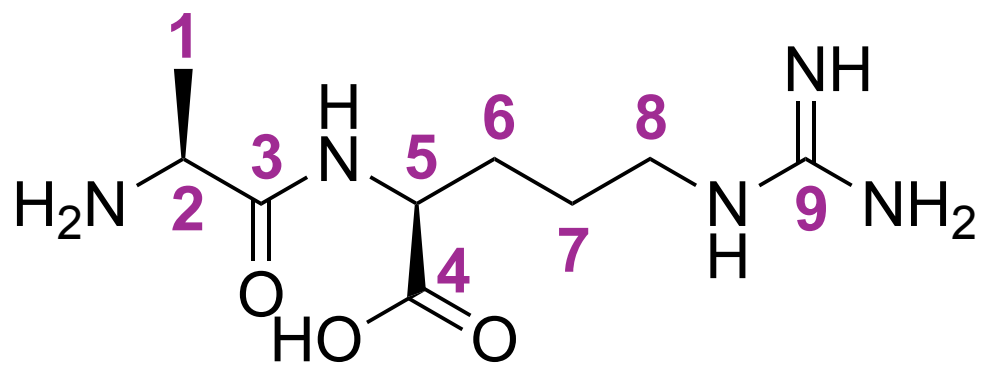

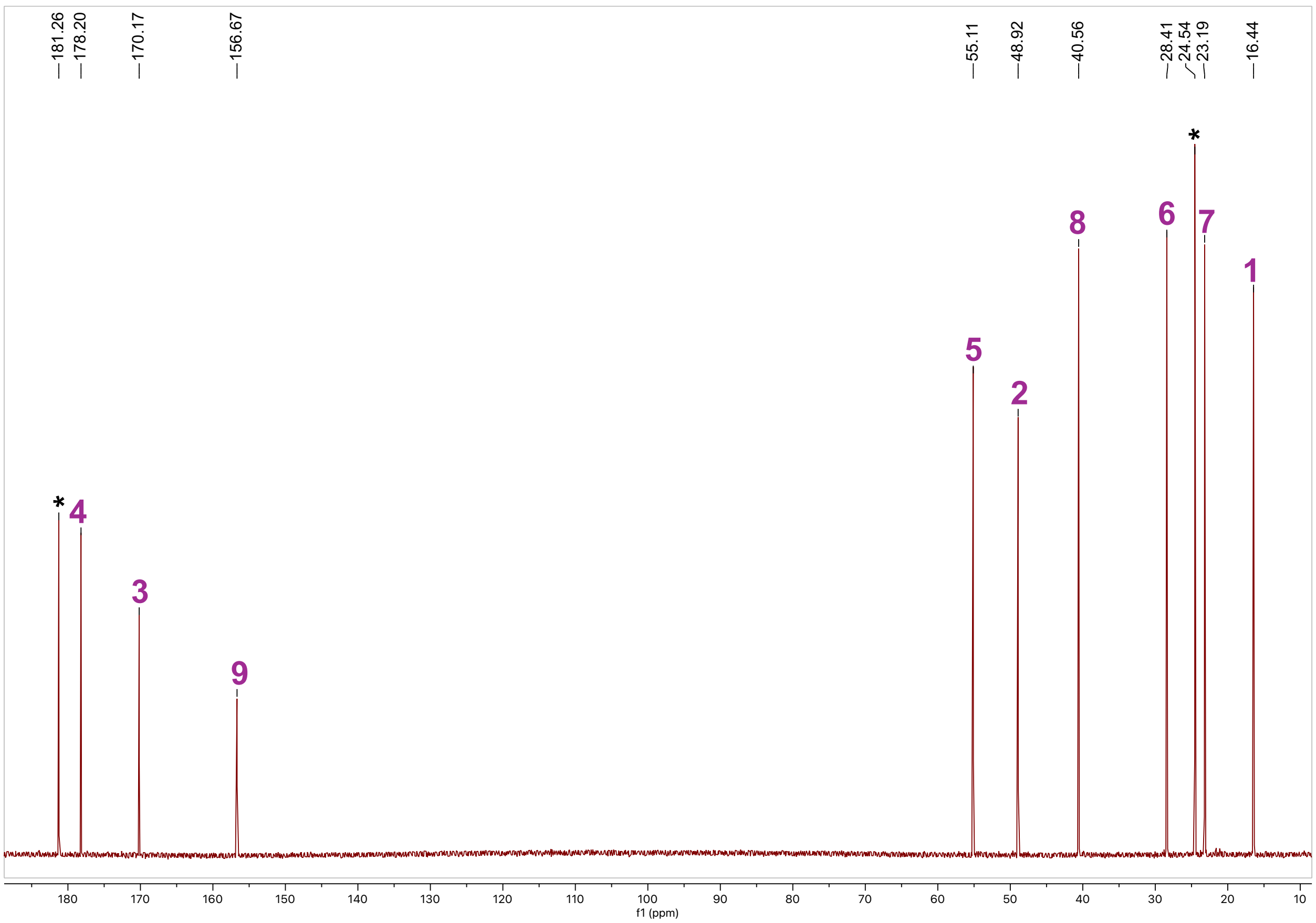

Figure S16. ^{13}C NMR spectrum (600 MHz) of Ala-Arg in D_2O. Asterisk (*) indicates acetate in the sample.

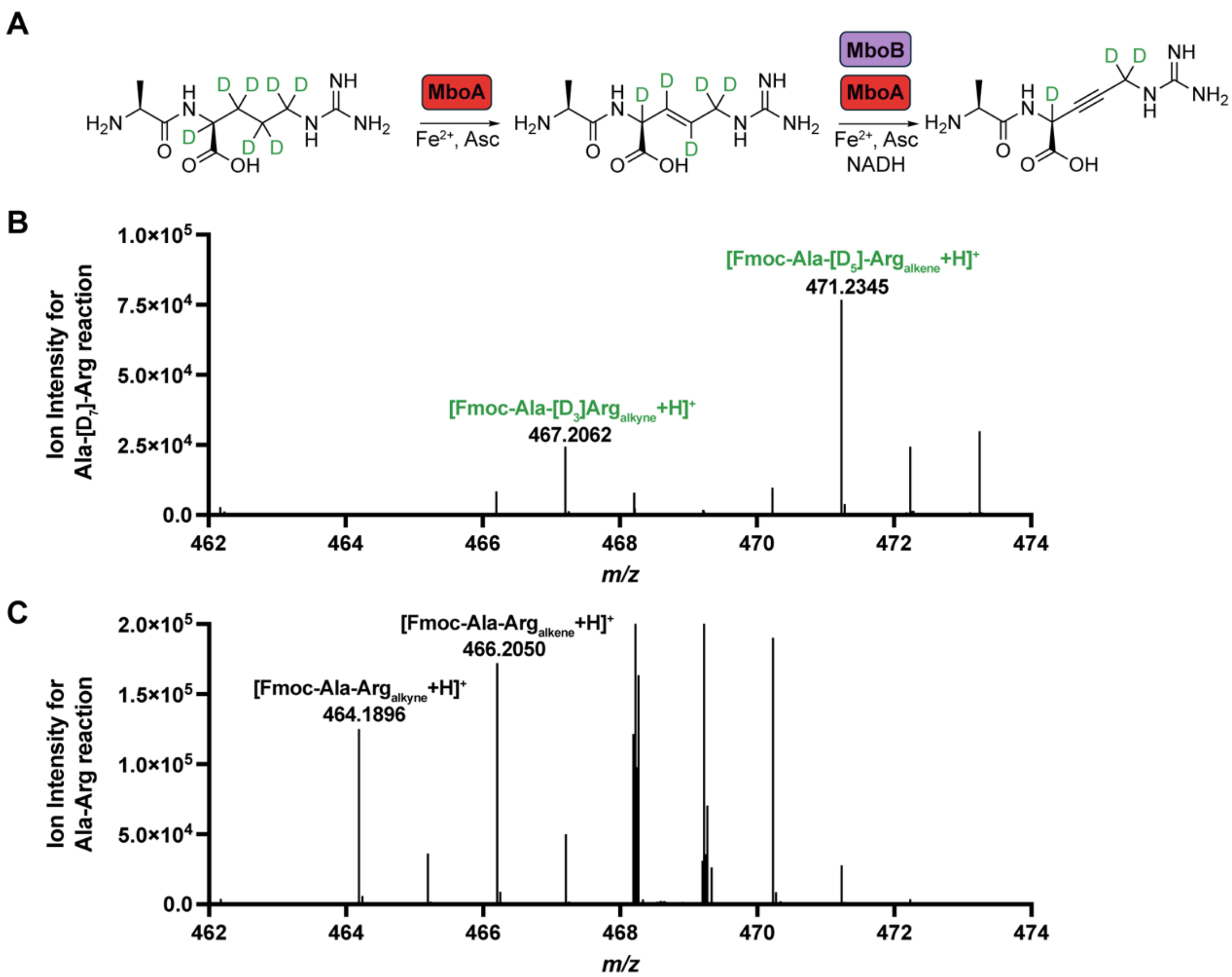

Figure S17. Oxidation of L-Ala-[D_7]-Arg by MboA and MboB resulted in the loss of two or four deuteriums that is consistent with alkene or alkyne products, respectively. (A) Reaction scheme for oxidation of Ala-[D_7]-Arg to the proposed Ala-[D_5]-Arg_{alkene} by MboA and oxidation to Ala-[D_3]-Arg_{alkyne} by MboA and MboB. (B) The mass spectrum of the Fmoc-derivatized MboABCD reaction containing [D_7]-Arg showing the product ions corresponding to Fmoc-Ala-[D_5]-Arg_{alkene} (calculated *m/z* 471.2399 $[M+H]^+$) and Fmoc-Ala-[D_3]-Arg_{alkyne} (calculated *m/z* 467.2117 $[M+H]^+$). (C) The mass spectrum of the Fmoc-derivatized MboABCD reaction containing Arg showing the product ions corresponding to Fmoc-Ala-Arg_{alkene} (calculated *m/z* 466.2085 $[M+H]^+$) and Fmoc-Ala-Arg_{alkyne} (calculated *m/z* 464.1928 $[M+H]^+$).

A

Enzyme type	Organism	z-score
Arginine deiminase (ADI)	*Mycoplasmopsis arginini*	30.3

Enzyme type	Organism	z-score
Dimethylarginine dimethylaminohydrolase (DDAH)	*Pseudomonas aeruginosa*	24.9

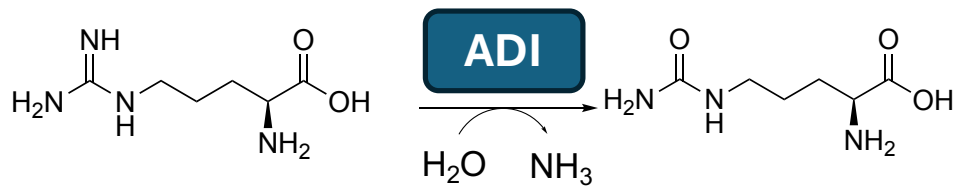

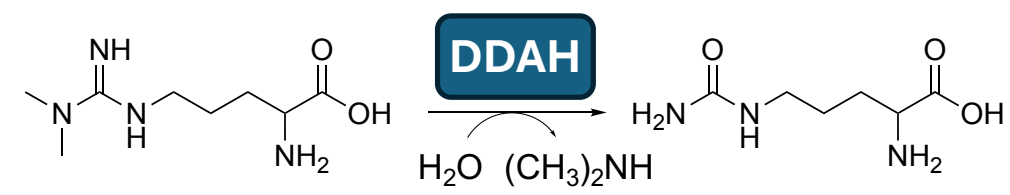

Enzyme type	Organism	z-score
Succinylarginine dihydrolase (SADH)	*Escherichia coli*	23.0

Enzyme type	Organism	z-score
Amidinotransferase (AT)	*Microseira wollei*	21.1

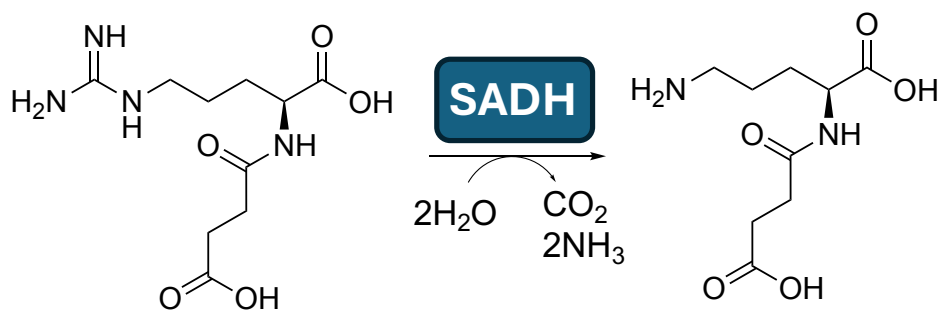

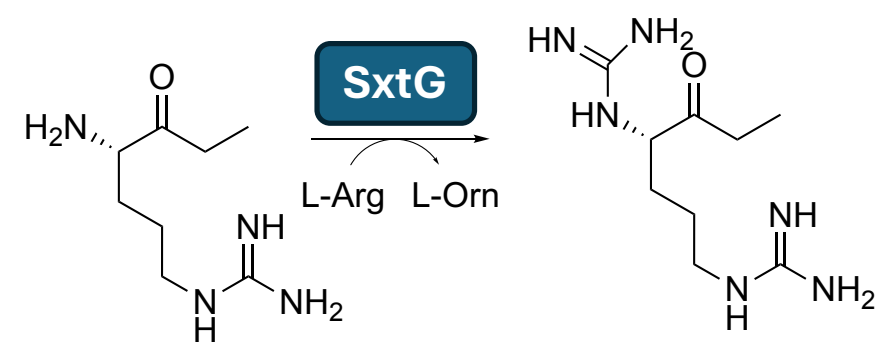

B

	D146 *	E199 *	H243 *	D245 *	C360 *
**MboE**	P R D	E G G D	H L D	C A T	
ADI	T R D	E G G D	H L D	C M S	
SADH	V A N	E G A A	H N D	C L R	
DDAH	V E D	E A G D	H L K	C M S	
AT	P R D	D A A D	H I D	C A T	

Figure S18. Dali search for structural homologs of MboE and sequence alignment. (A) Top hits of structural homologs of MboE from Dali search included arginine deiminase (ADI) (PDB 1lxy), dimethylarginine dimethylamino hydrolase (DDAH) (PDB 1h70), succinylarginine dihydrolase (SADH) (PDB 1ynh), and amidinotransferase (AT) (PDB 6u1r). (B) Sequence alignment of MboE with its structural homologs. Red asterisks indicate active site residues, and blue asterisks indicate substrate-binding residues. Putative active site residues in MboE are numbered. The MboE active site is most similar to arginine deiminase and amidinotransferase.

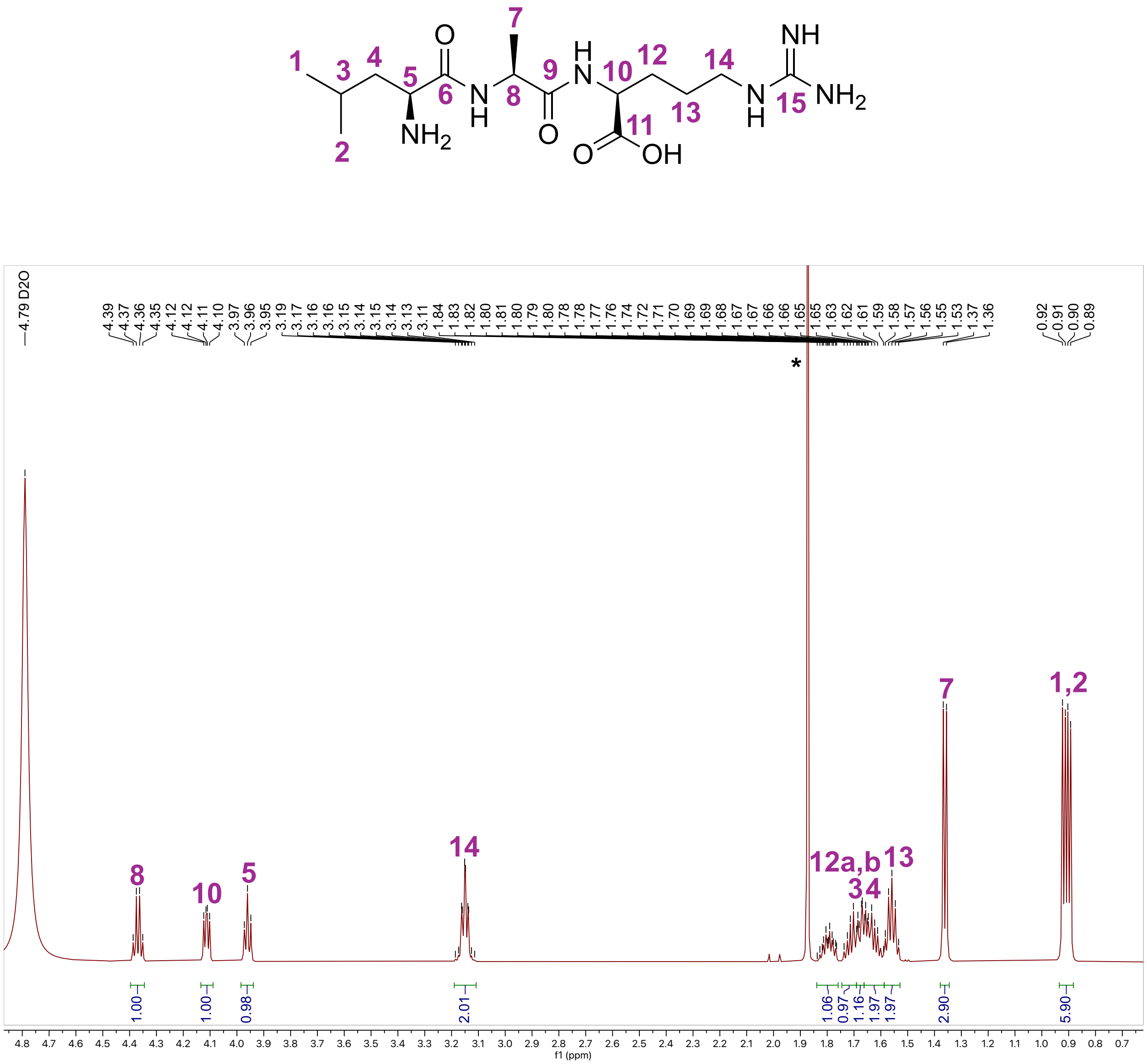

Figure S19. ^{1}H NMR spectrum (600 MHz) of Leu-Ala-Arg in D_2O. Asterisk (*) indicates acetate in the sample.

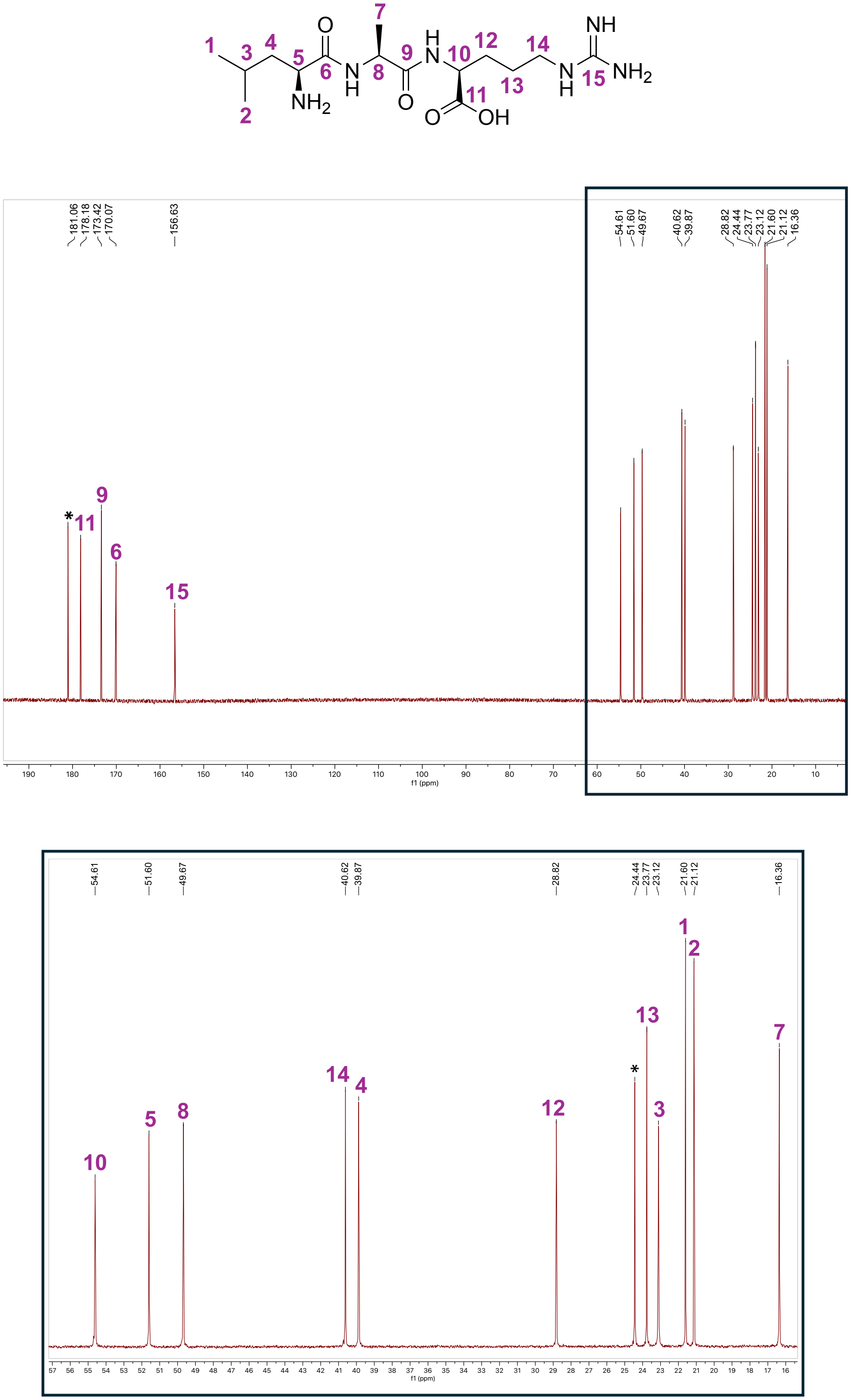

Figure S20. ^{13}C NMR spectrum (600 MHz) of Leu-Ala-Arg in D_2O. Asterisk (*) indicates acetate.

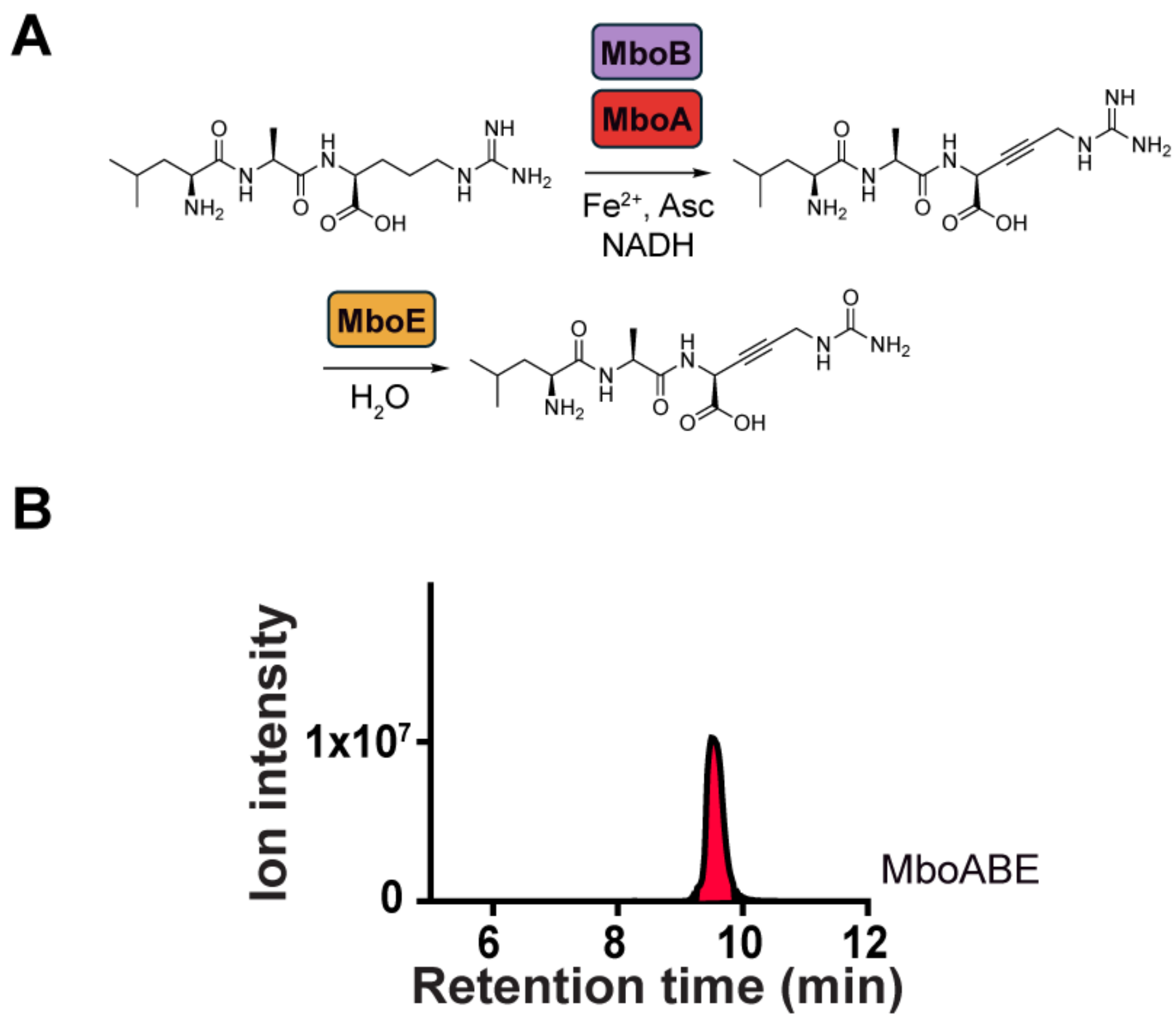

Figure S21. The synthetic Leu-Ala-Arg tripeptide is also a substrate of MboA, MboB, and MboE. (A) Scheme for conversion of Leu-Ala-Arg to alkyne-containing citrulline tripeptide *in vitro* catalyzed by MboA, MboB, and MboE. MboA and MboB can catalyze the conversion of Leu-Ala-Arg to Leu-Ala-Arg_{alkyne}, which can be converted to Leu-Ala-Cit_{alkyne} by MboE. (B) Extracted ion chromatogram of Leu-Ala-Cit_{alkyne} (calculated *m/z* 578.2609 $[Fmoc\text{-}M+H]^+$) from LC-HRMS analysis of the *in vitro* reaction containing synthetic Leu-Ala-Arg and MboABE.

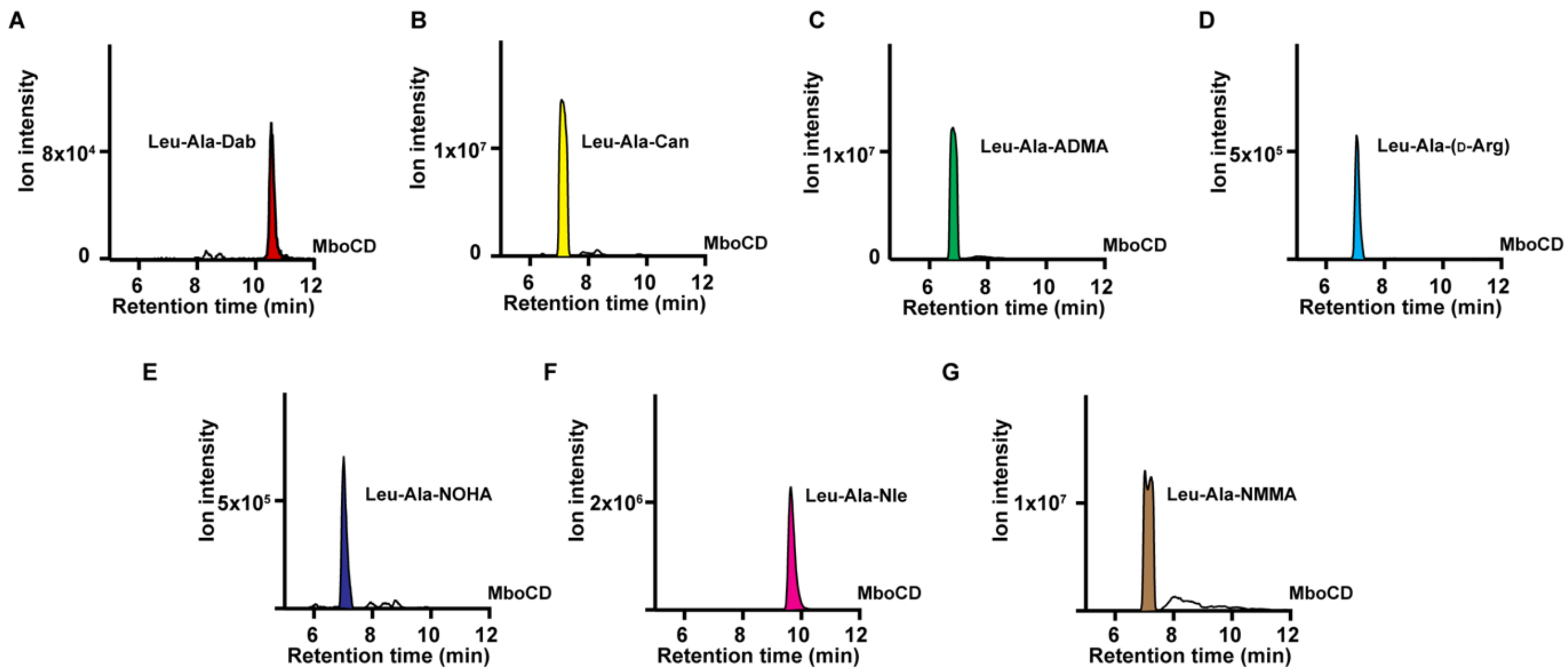

Figure S22. MboC and MboD catalyzed the formation of different Leu-Ala-containing tripeptides. From LC-HRMS analysis of MboCD reactions containing Leu, Ala, and a varying amino acid, EICs are shown for the tripeptide products, including (A) Leu-Ala-Dab (*m/z* 747.3388 [(bis-Fmoc)-M+H]$^+$), (B) Leu-Ala-Can (*m/z* 583.2875 [Fmoc-M+H]$^+$), (C) Leu-Ala-ADMA (*m/z* 609.3395 [Fmoc-M+H]$^+$), (D) Leu-Ala-(D-Arg) (*m/z* 581.3082 [Fmoc-M+H]$^+$), (E) Leu-Ala-NOHA (*m/z* 597.3031 [(Fmoc-M+H]$^+$), (F) Leu-Ala-Nle (*m/z* 538.2912 [Fmoc-M+H]$^+$), and (G) Leu-Ala-NMMA (*m/z* 595.3239 [Fmoc-M+H]$^+$). All reactions were derivatized using Fmoc-Cl before LC-HRMS analysis. Dab, L-2,4-diaminobutyric acid; Can, L-canavanine; ADMA, N^G, N^G-dimethyl-L-arginine; D-Arg, D-arginine; NOHA, N^G-hydroxy-L-arginine; Nle, L-norleucine; NMMA, N^G-monomethyl-L-arginine. The Dab-containing peptide with single Fmoc derivatization was also detected but not shown. Formation of the Leu-Ala-Xaa tripeptides was observed for all the Xaa substrates tested in MboC and MboD reaction. Reactions and analysis were repeated on a different day and similar results were observed.

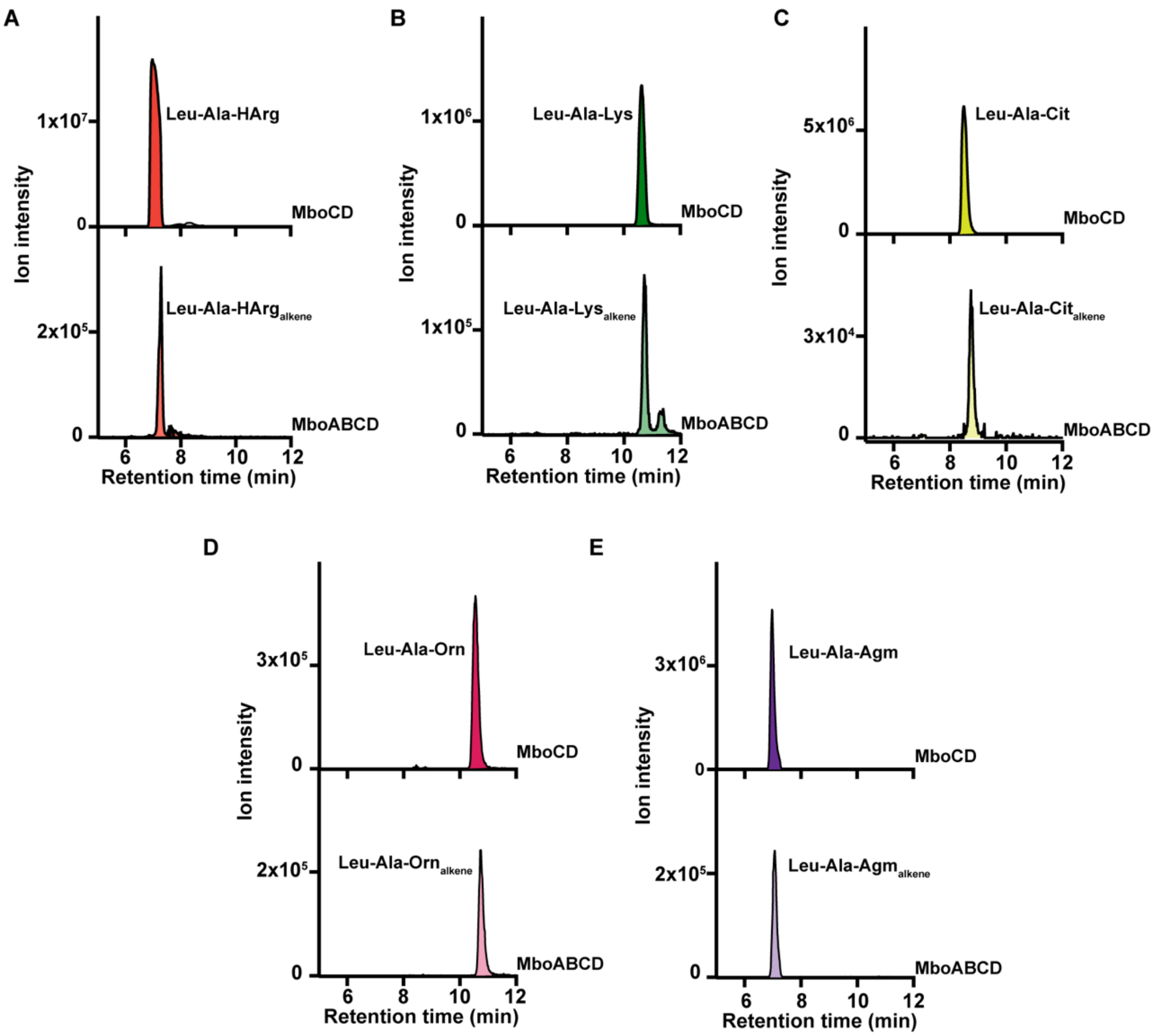

Figure S23. Two-electron oxidation products were detected in the MboA and MboB reactions containing different tripeptide substrates. From LC-HRMS analysis of MboCD and MboABCD sequential one-pot reactions containing different substrates, EICs are shown for tripeptides and their respective two-electron oxidized products, including (A) Leu-Ala-HArg (*m/z* 595.3239 $[Fmoc\text{-}M+H]^+$, top) and Leu-Ala-$HArg_{alkene}$ (*m/z* 593.3082 $[Fmoc\text{-}M+H]^+$, bottom), (B) Leu-Ala-Lys (*m/z* 775.3701 $[(bis\text{-}Fmoc)\text{-}M+H]^+$, top) and Leu-Ala-$Lys_{alkene}$ (*m/z* 773.3545 $[(bis\text{-}Fmoc)\text{-}M+H]^+$, bottom), (C) Leu-Ala-Cit (*m/z* 582.2922 $[Fmoc\text{-}M+H]^+$, top) and Leu-Ala-Cit_{alkene} (*m/z* 580.2766 $[Fmoc\text{-}M+H]^+$, bottom), (D) Leu-Ala-Orn (*m/z* 761.3545 $[(bis\text{-}Fmoc)\text{-}M+H]^+$, top) and Leu-Ala-$Orn_{alkene}$ (*m/z* 759.3388 $[(bis\text{-}Fmoc)\text{-}M+H]^+$, bottom), and (E) Leu-Ala-Agm (*m/z* 537.3184 $[Fmoc\text{-}M+H]^+$, top) and Leu-Ala-Agm_{alkene} (*m/z* 535.3027 $[Fmoc\text{-}M+H]^+$, bottom). All reactions were derivatized using Fmoc-Cl before LC-HRMS analysis. HArg, L-homoarginine; Lys, L-lysine; Cit, L-citrulline; Orn, L-ornithine; Agm, agmatine. Lys and Orn-containing peptides with single Fmoc derivatization were also detected but not shown. Reactions and analysis were repeated on a different day and similar results were observed.

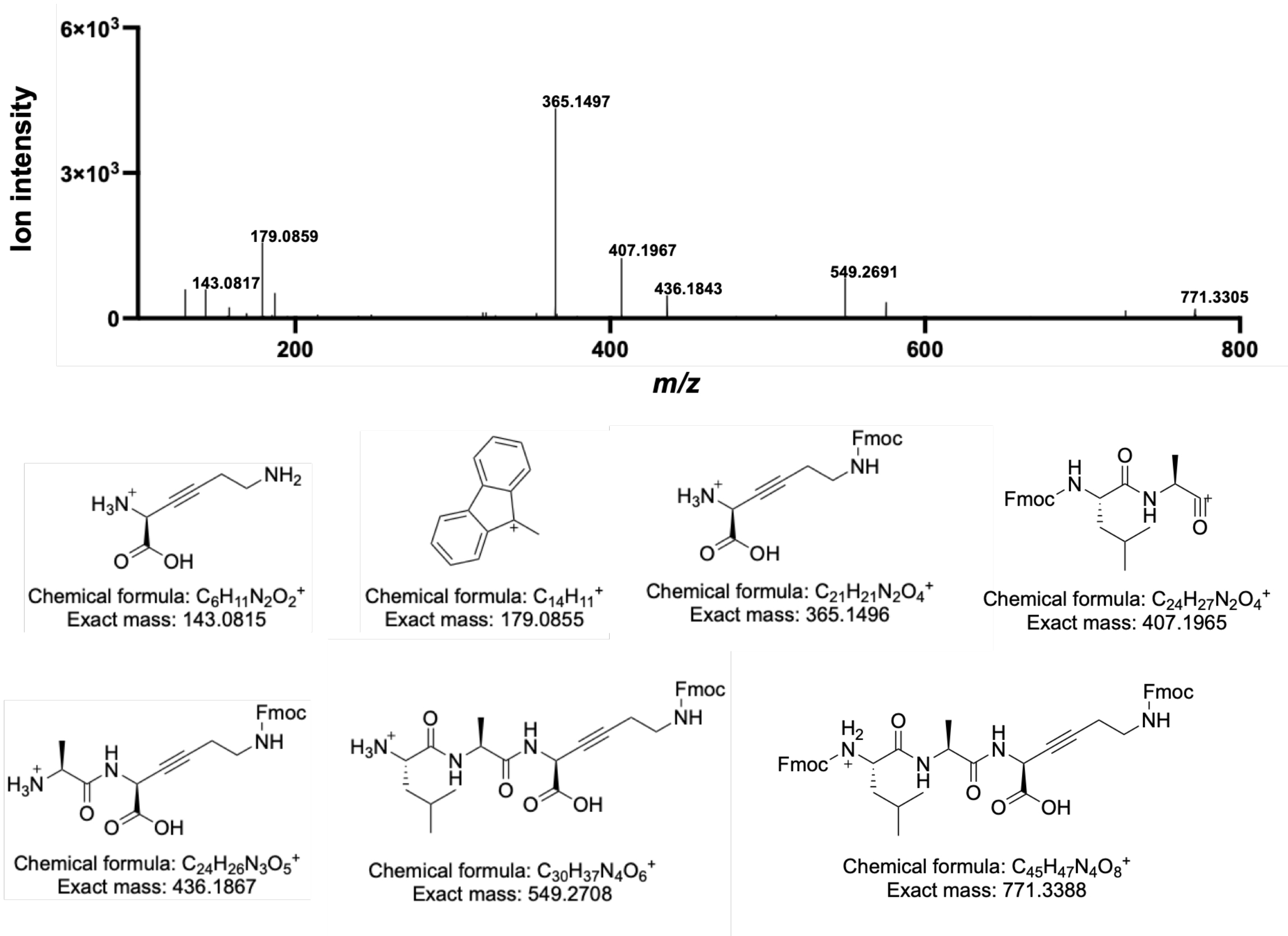

Figure S24. Tandem mass spectrum of the proposed Fmoc-Leu-Ala-Lys$_{alkyne}$ product. Fragmentation was performed at 20 eV collision energy. Parent ion: (bis-Fmoc)-Leu-Ala-Lys$_{alkyne}$ (calculated *m/z* 771.3388 $[M+H]^+$).

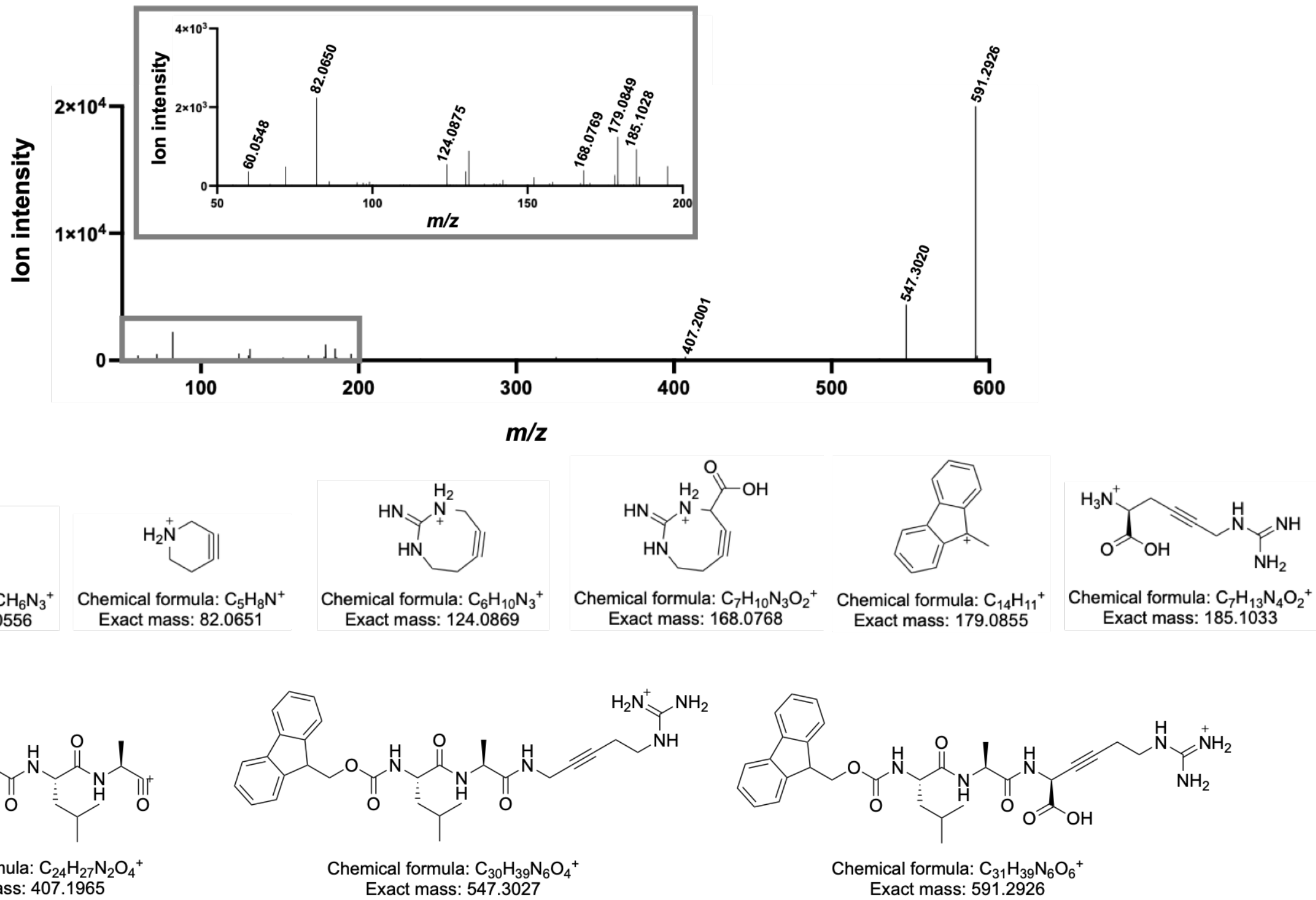

Figure S25. Tandem mass spectrum of the proposed Fmoc-Leu-Ala-HArg$_{alkyne}$ product. Fragmentation was performed at 20 eV collision energy. Parent ion: Fmoc-Leu-Ala-HArg$_{alkyne}$ (calculated *m/z* 591.2926 $[M+H]^+$).

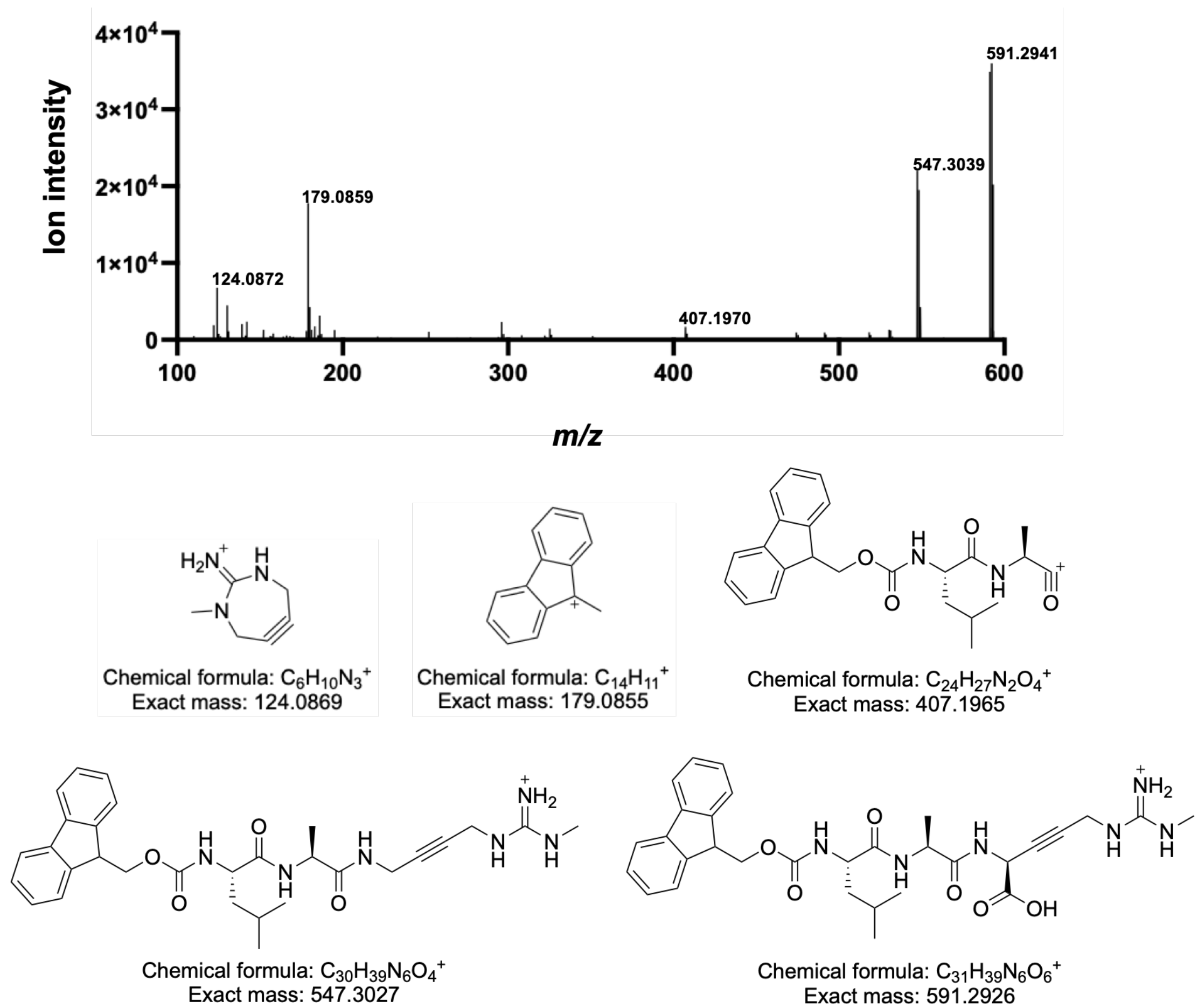

Figure S26. Tandem mass spectrum of the proposed Fmoc-Leu-Ala-NMMA$_{alkyne}$ product. Fragmentation was performed at 20 eV collision energy. Parent ion: Fmoc-Leu-Ala-NMMA$_{alkyne}$ (calculated *m/z* 591.2926 $[M+H]^+$).

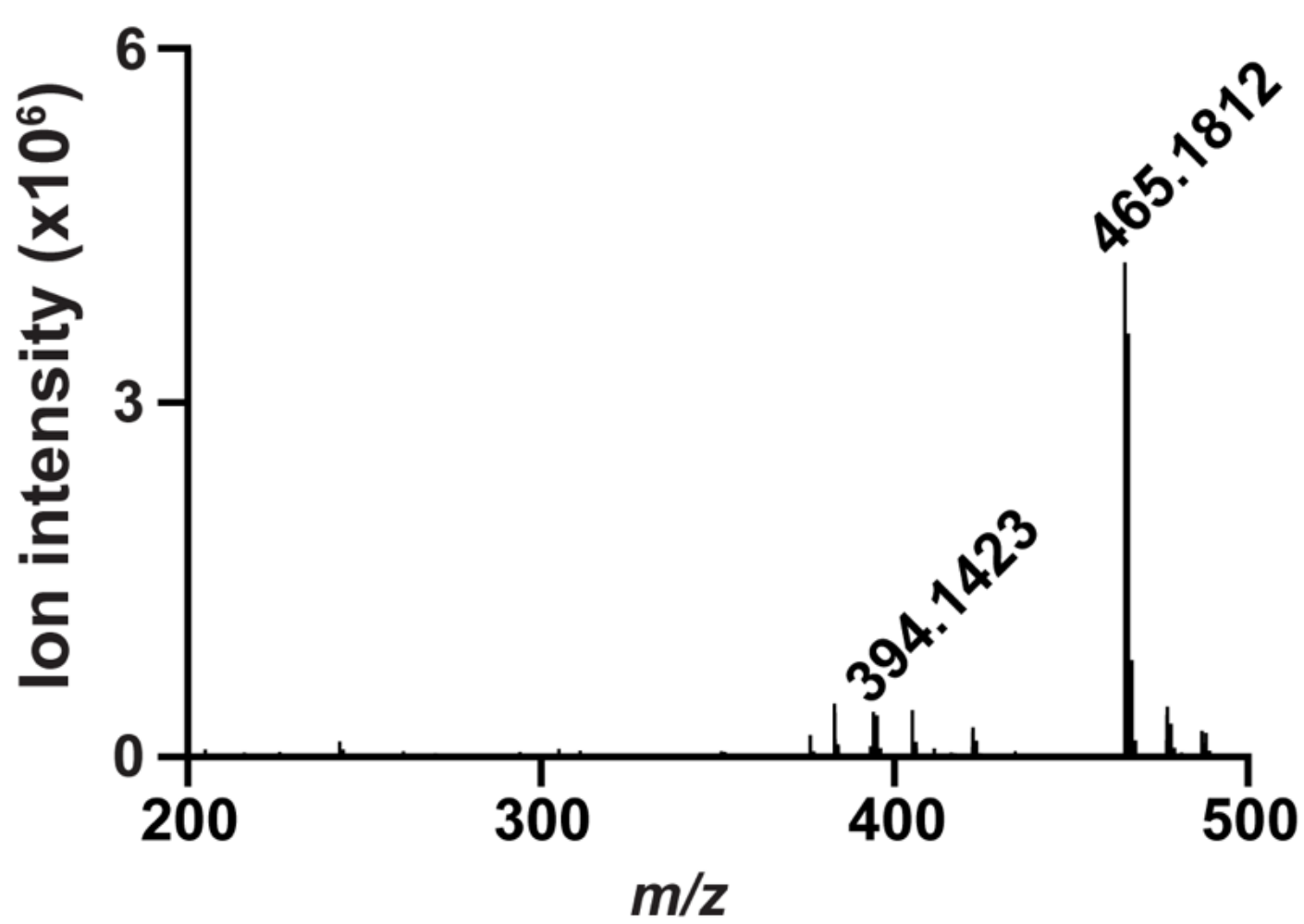

Figure S27. Mass spectrum of purified Ala-Cit$_{alkyne}$ after Fmoc derivatization from LC-MS analysis. The dominant species is Fmoc-Ala-Cit$_{alkyne}$ (calculated *m/z* 465.1769 $[M+H]^+$). A small amount of Fmoc-Cit$_{alkyne}$ (calculated *m/z* 394.1397 ($[M+H]^+$) was also detected.

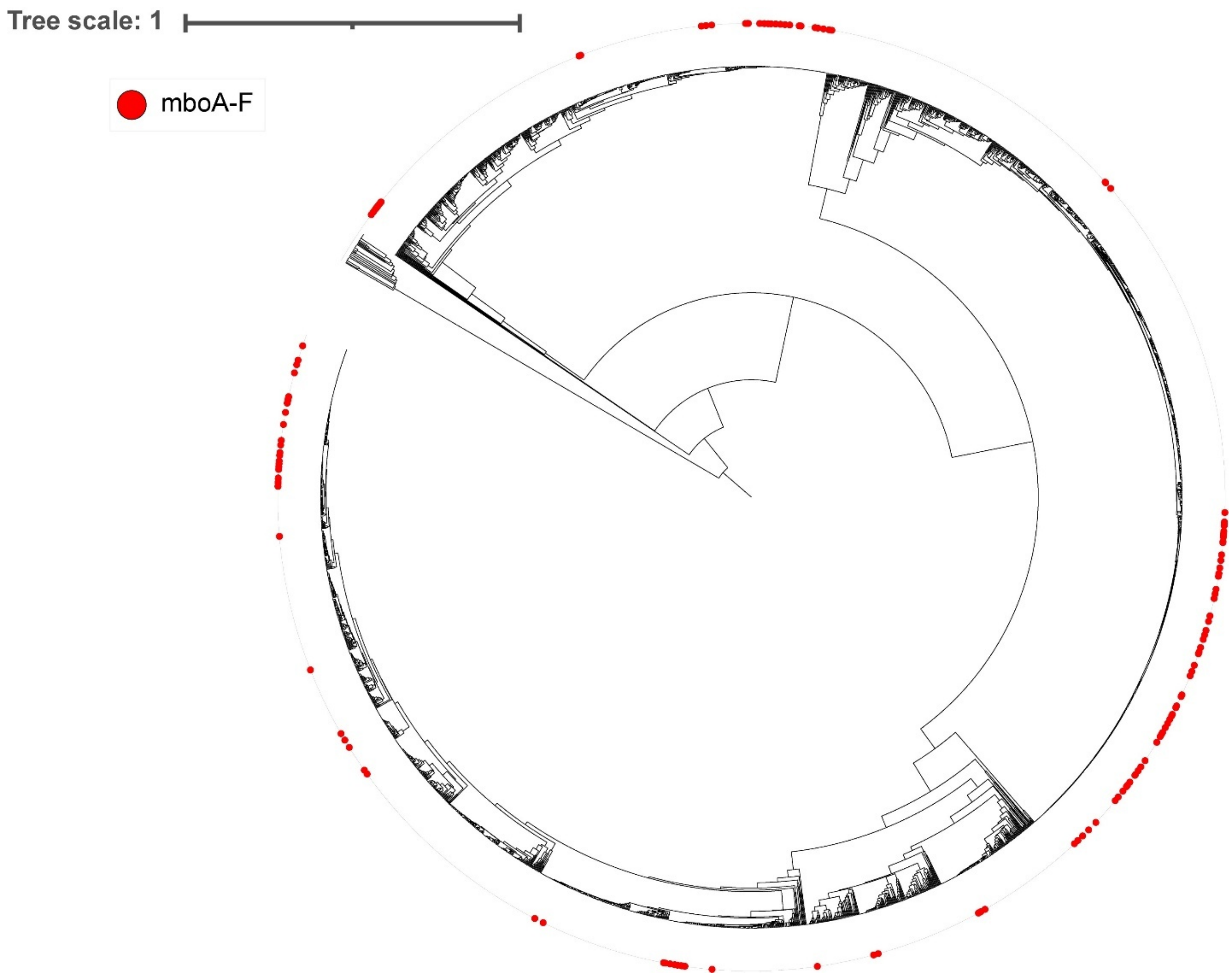

Figure S28. The distribution of the *mangotoxin biosynthetic operon* (*mbo*) in 3071 organisms of *Pseudomonas syringae* group (taxon ID: 136849). Red circles indicate the 335 organisms that contain *mboA-F*. The scale represents the number of amino acid substitutions per site.

Supporting References:

(1) Guo, Q.; Vitro, C. N.; Crawford, D. M.; Li, B. A Diazeniumdiolate Signal in *Pseudomonas syringae* Upregulates Virulence Factors and Promotes Survival in Plants. *Mol. Plant-Microbe Interact.* **2024**, *37* (11), 776–783.
(2) Gasson, M. J. Indicator Technique For Antimetabolic Toxin Production By Phytopathogenic Species Of *Pseudomonas*. *Appl. Environ. Microbiol.* **1980**, *39* (1), 25–29.
(3) Smith, C. A.; Want, E. J.; O'Maille, G.; Abagyan, R.; Siuzdak, G. XCMS: Processing Mass Spectrometry Data For Metabolite Profiling Using Nonlinear Peak Alignment, Matching, And Identification. *Anal. Chem.* **2006**, *78* (3), 779–787.
(4) Dieckman, L.; Gu, M.; Stols, L.; Donnelly, M. I.; Collart, F. R. High Throughput Methods For Gene Cloning And Expression. *Protein Expr. Purif.* **2002**, *25* (1), 1–7.
(5) Madeira, F.; Madhusoodanan, N.; Lee, J.; Eusebi, A.; Niewielska, A.; Tivey, A. R. N.; Lopez, R.; Butcher, S. The EMBL-EBI Job Dispatcher Sequence Analysis Tools Framework In 2024. *Nucleic Acids Res.* **2024**, *52* (W1), W521–W525.
(6) Waterhouse, A. M.; Procter, J. B.; Martin, D. M. A.; Clamp, M.; Barton, G. J. Jalview Version 2—A Multiple Sequence Alignment Editor And Analysis Workbench. *Bioinformatics* **2009**, *25* (9), 1189–1191.
(7) Abramson, J.; Adler, J.; Dunger, J.; Evans, R.; Green, T.; Pritzel, A.; Ronneberger, O.; Willmore, L.; Ballard, A. J.; Bambrick, J.; et al. Accurate Structure Prediction Of Biomolecular Interactions With AlphaFold 3. *Nature* **2024**, *630* (8016), 493–500.
(8) Katoh, K.; Rozewicki, J.; Yamada, K. D. MAFFT Online Service: Multiple Sequence Alignment, Interactive Sequence Choice and Visualization. *Briefings Bioinf.* **2019**, *20* (4), 1160–1166.
(9) Badding, E. D.; Kissman, E. N.; Velculescu, S. V.; Chang, M. C. Y. Discovery Of A Structurally Distinct Acetylenase In The Biosynthesis Of Mangotoxin. *J. Am. Chem. Soc.* **2026**, *148* (18), 18724–18732.
